# Testing the limits: serial crystallography using unpatterned fixed targets

**DOI:** 10.1107/S2052252525008371

**Published:** 2025-10-13

**Authors:** Alexander Gorel, Robert L. Shoeman, Elisabeth Hartmann, Stanislaw Nizinski, Martin V. Appleby, Emma V. Beale, Florian Dworkowski, Guillaume C. Gotthard, John H. Beale, James Holton, R. Bruce Doak, Thomas R. M. Barends, Ilme Schlichting

**Affiliations:** a Max Planck Institute for Medical Research Jahnstrasse 29 69120Heidelberg Germany; b Swiss Light Source Paul Scherrer Institute Forschungsstrasse 111 5232Villigen Switzerland; c Molecular Biophysics and Integrated Bioimaging Division Lawrence Berkeley National Laboratory Berkeley California USA; Uppsala University, Sweden

**Keywords:** serial crystallography, SSX, SFX, radiation damage, metalloproteins, fixed target, SOS chip, dose rate, room temperature

## Abstract

Sheet-on-sheet (SOS) fixed-target chips are arguably the most versatile, cheapest and simplest sample-delivery method for ambient-temperature data acquisition using serial crystallography approaches at synchrotrons and X-ray free-electron lasers (XFELs). Their defining feature, the absence of any hard-patterned restrictions around crystals, is their strength as it removes limitations on crystal sizes or environments. However, it is also their weakness when it comes to limiting undesired effects on yet-to-be-irradiated crystals due to diffusing heat, radicals or gas originating from previous exposures. We explored whether SOS chips can be used for damage-free serial data collection on the new ID29 beamline at the ESRF-EBS, a fourth-generation synchrotron light source, as well as at the new Cristallina-MX station at SwissFEL. We collected serial data sets from microcrystals of the hemoprotein DtpAa, which was reported to have a highly radiation-sensitive iron–water bond length. The data sets differ in step size between exposures within and between lines of a serpentine-like data-acquisition scan. We observe no significant changes in the distance of the water ligand of the heme in the structures obtained from the ID29 SSX data. However, when compared with those collected at Cristallina-MX, the diffraction intensities collected at ID29 suggest global damage akin to Bragg termination occurring during the 90 µs exposure at ID29. Moreover, differences in the heme geometry and the proximal histidine–iron bond length point to local damage in all ID29 data sets regardless of the X-ray spacing. SFX data collected at Cristallina-MX show a phase transition of the DtpAa crystal lattice for X-ray step sizes of ≤20 µm. This phase transition might be caused by heating and/or hydrogen-gas-induced crystal dehydration. Vigilance remains required to safeguard against radiation damage at fourth-generation synchrotrons and XFELs.

## Abstract 2

The influence of the X-ray step size on radiation damage occurring in serial data collection from a metalloprotein using sheet-on-sheet fixed-target chips was investigated at fourth-generation synchrotron and XFEL beamlines.

## 1. Introduction

Radiation damage is a nemesis of protein crystallography (Garman & Weik, 2017 ▸; Holton, 2009 ▸), often affecting both the resolution of the diffraction data (global damage) and the chemical properties of the sample investigated (local damage). The underlying reason is photoionization, followed by cascades of secondary ionization events and radical chemistry. Many of the sample-modifying (*i.e.* damaging) events can be slowed significantly by keeping the crystals at cryogenic temperature during the X-ray exposure, as this slows the diffusion of radiation-induced radicals through the crystal. Alternatively, the X-ray dose absorbed during data collection can be distributed over many crystals. This latter approach is taken to the extreme in serial data-collection approaches, which typically acquire single diffraction snapshots from many thousands of microcrystals.

**Figure 1:**
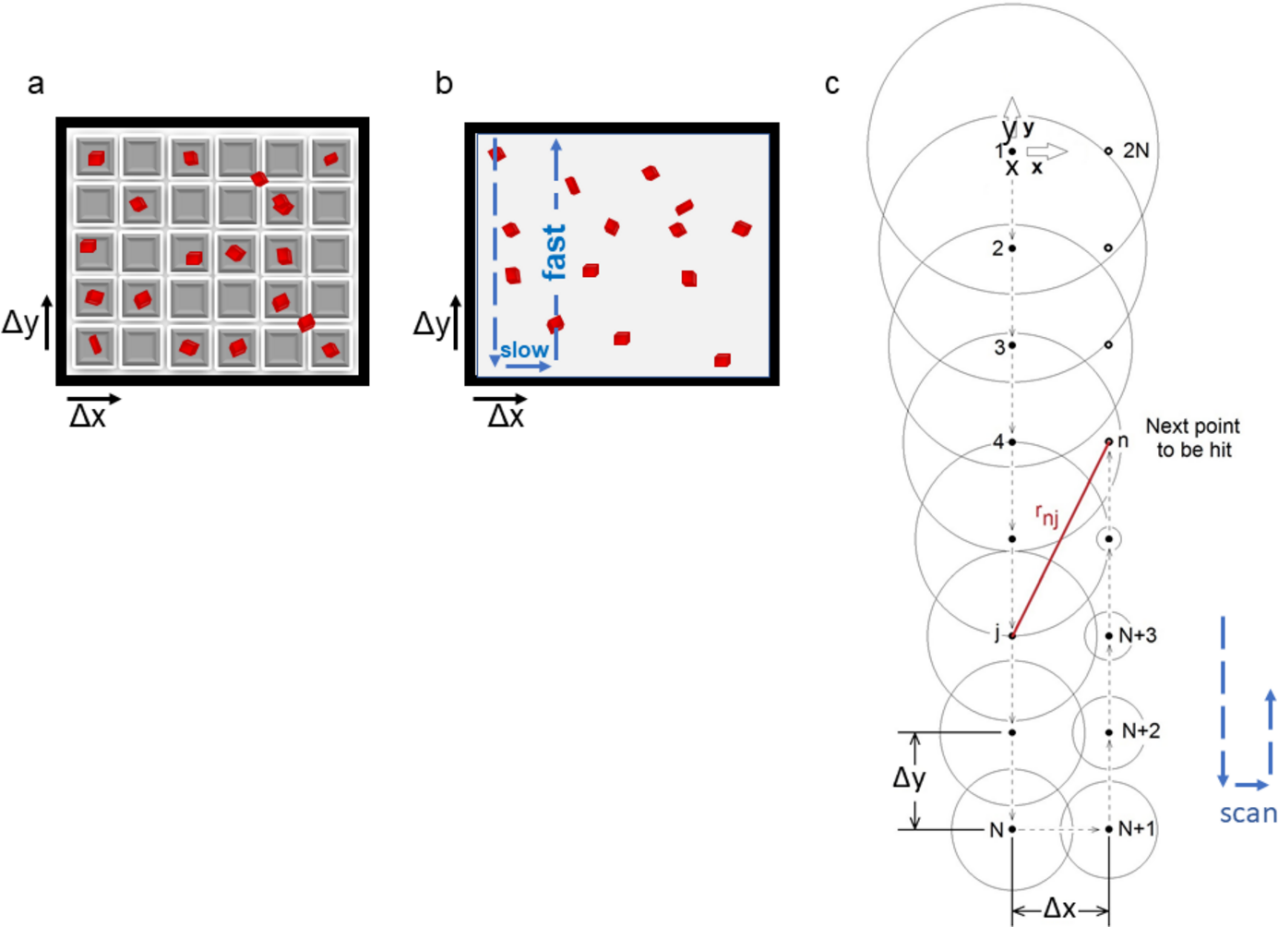
(*a*) Patterned (apertured) chips contain a regular array of blind recesses or cavitied apertures where crystals can locate. These wells fix the raster-scan step sizes (Δ*y* and Δ*x*) of the well array. The separating walls between the localization wells tend to shield unexposed crystals from shockwaves, heat, radicals and gas generated produced by previous X-ray exposures. (*b*) In SOS chips the crystals are randomly distributed within a thin film sealed between two stretched polymer foils. Since crystals need not funnel into structured features, there are no limits on crystal size and only minor considerations regarding media; this makes SOS chips uniquely suited for investigations using true nanocrystals or membrane-protein crystals grown in LCP. Moreover, scan-step sizes within and between individual scan lines can be chosen freely. Since the raster-scan directions (up–down versus side-to-side) vary from one facility to the next, it is useful to denote them as inter-line or fast (here Δ*y*) and intra-line or slow (here Δ*x*). (*c*) Schematic illustrating radiation damage spreading in a step-by-step periodic exposure of a SOS chip (Doak *et al.*, 2024 ▸). Shown are one full downwards scan of exposures 1 to *N* in steps of Δ*y*, followed by a horizontal step of Δ*y* = Δ*x* and the few first exposures of the upwards return scan from *N* + 1 to 2*N* + 1 (blue arrows). Here, damage is represented simply as spreading at constant radial speed with the increment Δ*y* and the time between exposures are chosen such that damage spreads by only Δ*y*/10 in the time between exposures. If actual diffusion constants are available, quite accurate calculations can easily be carried out (see, for example, the Supporting Information for X-ray-induced heating/gas diffusion). The motion diagrams are for the beamtimes at Cristallina-MX; the fast and slow axes are switched for ID29.

For serial data-collection approaches, the rapid delivery and replenishment of fresh microcrystals into the X-ray beam is essential. A number of delivery approaches exist that differ in complexity, sample efficiency and potential applications (Grünbein & Kovacs, 2019 ▸; Martiel *et al.*, 2019 ▸). Arguably, the sheet-on-sheet (SOS) fixed target (Doak *et al.*, 2018 ▸, 2024 ▸) is not only one of the cheapest and most straightforward approaches, but also one of the most versatile from a sample perspective, since it places no limits on crystal size, homogeneity or crystal-growth medium [liquid or lipidic cubic phase (LCP)]. This is because the chip consists simply of two layers of polymer foil with the sample sandwiched in between; it contains no hard-patterned array of crystal-localization wells [Figs. 1 ▸(*a*) and 1 ▸(*b*)] that can form physical barriers between crystals. Like all fixed targets, the SOS chip relies on a two-dimensional *x*, *y* scan of the crystal-containing area, possibly with small adjustments in position along the X-ray beam direction (*z* direction) to keep the crystal in the X-ray focus, and thereby also maintain a fixed sample-to-detector distance. The finer the raster scan – that is, the more closely spaced the X-ray exposures – the higher the number of crystal hits that can be obtained from the chip, increasing the efficiency of sample usage. However, a tighter raster scan may increase the likelihood of radiation damage. An obvious concern is that with large crystals and small steps, crystals can be exposed more than once. More subtle difficulties can also arise. Since protein crystals contain a large fraction of water, the electrons generated during X-ray exposure result, among other things, in water radiolysis. This in turn generates radicals that can infuse and damage neighboring, yet-to-be-probed crystals (or parts thereof), particularly in SOS chips given the absence of a physical barrier between crystals. Since this effect is time- and distance-dependent, the spacing between scan lines can be critical [Fig. 1 ▸(*c*)] (Doak *et al.*, 2024 ▸). To investigate this issue, we used SOS chips for microcrystal delivery into the X-ray beam to collect room-temperature (i) serial synchrotron crystallography (SSX) data on the new ID29 beamline at the ESRF and (ii) serial femtosecond crystallography (SFX) data within the commissioning of the new Cristallina-MX end­station at SwissFEL.

Many enzymes use redox-sensitive cofactors such as flavins or hemes to catalyze reactions. Heme-protein crystals are exquisitely sensitive to X-ray photoreduction; a dose of ∼40 kGy already yields 50% ferrous iron (Pfanzagl *et al.*, 2020 ▸). They are thus suitable systems to investigate radiation damage. We studied a well established model system, the extracellular dye-type heme peroxidase DtpAa (Ebrahim *et al.*, 2019 ▸), and monitored the bond length between a water molecule and the heme iron, as well as the geometry of the heme, as a function of the X-ray raster-scan step size in SSX and SFX data collections. We identify a minimum translation step size of 25 µm for serial crystallography with SOS chips in the free-electron laser measurements, below which radiation damage is observed in DtpAa. While no step-size-dependent damage is observed for SSX data collected at ID29, global and local radiation damage does appear to occur at all step sizes tested during the 90 µs exposure.

## 2. Materials and methods

### 2.1. Sample preparation

DtpAa was expressed and purified as described previously (Ebrahim *et al.*, 2019 ▸). Microcrystals of DtpAa were grown in batch, mixing 1 ml 20–23% PEG 6000, 200 m*M* Na–HEPES pH 7.0 and 1 ml 3–4 mg ml^−1^ DtpAa (Ebrahim *et al.*, 2019 ▸). The crystals showed a broad size distribution (see Supplementary Fig. S1), with an average size of about 20 µm. Gravity-pelleted microcrystals were mixed with artificial mother liquor (75 m*M* Na–HEPES pH 7.0, 10% PEG 6000) enriched with hydroxyethyl cellulose (HEC) using coupled gas-tight Hamilton syringes. HEC was added to prevent microcrystals settling in the vertically mounted SOS chips during data collection and to improve the wetting of the SOS membranes. Two different crystal batches were used (see Table 1 ▸ and Supplementary Fig. S1): Batch I was used for data collection at Cristallina-MX (June 2023, 2.0% HEC), whereas Batch II was used at ID29 (February 2024, 2.5% HEC) and at Cristallina-MX (September 2023, 2.5% HEC) for follow-up data collection. We used the most recent version of the SOS chips (Doak *et al.*, 2024 ▸) with ∼2 × 2 cm windows, consisting of two 2.5 µm thick Etnom foils (Chemplex). 10–15 µl of microcrystal slurry was applied in small droplets onto the lower foil, the upper foil was added and the microcrystalline slurry was pressed into a thin and uniform layer using the SOS sample press (Doak *et al.*, 2024 ▸).

### 2.2. Data collection at ID29

Diffraction data were collected on beamline ID29 at the ESRF (https://www.esrf.fr/id29; Orlans *et al.*, 2025 ▸) using a photon energy of 11.56 keV and an X-ray focus of 2 × 4 µm (vertical × horizontal). For a ring current of 200 mA, the flux of the focused beam (after cleaning the tails with a pinhole aperture) was 1.6 × 10^15^ photons s^−1^ at the sample position (Orlans *et al.*, 2025 ▸). The ring current was 68 mA in February 2024 (100% transmission). Immediately after loading, the SOS chips were mounted on the goniometer and SSX data were collected. To this end, the SOS chips were rastered from top to bottom in a serpentine-like pattern (Fig. 1 ▸) in horizontal lines (intra-line step size Δ*x*, referred to as the ‘fast’ scan direction) followed by a vertical step (inter-line step size Δ*y*, the ‘slow’ scan direction) before the next horizontal leg. The exposure time was 90 µs and both the X-ray and JUNGFRAU 4M detector sampling rates were 231.25 Hz. The (Δ*x*, Δ*y*) spacings between exposures were 100 × 100, 75 × 75, 50 × 50, 25 × 25, 25 × 100, 25 × 75, 25 × 50 and 15 × 100 µm. The average diffraction-weighted dose (Zeldin *et al.*, 2013 ▸; Dickerson *et al.*, 2020 ▸, 2024 ▸) was ∼0.88 MGy (see Table 1 ▸).

### 2.3. Data collection at Cristallina-MX

We collected analogous data sets at the Cristallina-MX instrument (https://www.psi.ch/en/swissfel/cristallina/cristallina-mx-0) at SwissFEL, again using the SOS chips. The mean photon energy was 12.04 keV (measured via a scan of the Bernina monochromator), the X-ray focus was approximately 5 × 5 and 6 × 6 µm (FWHM) and the pulse energy of the attenuated XFEL beam at the sample position was ∼105 and 120 µJ in the June and September experiments, respectively. The X-ray pulse duration for the June experiment was estimated to be between 25 and 45 fs (FWHM) based on the pulse energy and was measured to be 25 fs (FWHM) in September 2023 using the SwissFEL passive streaker (Dijkstal *et al.*, 2022 ▸). In contrast to ID29, at Cristallina-MX the scan lines were rastered vertically with intra-line spacing Δ*y* (‘fast’ scan direction), with inter-line spacing Δ*x* (‘slow’ scan direction). In the experiment performed in June 2023 the spacings (Δ*x*, Δ*y*) between recorded patterns were 100 × 100, 75 × 75, 50 × 50, 25 × 25, 50 × 25, 75 × 50, 75 × 25 and 50 × 25 µm. In the quest to observe the onset of radiation damage, we performed a follow-up experiment with shorter spacings in September 2023 with spacings of 50 × 50, 25 × 25, 20 × 20, 20 × 15, 20 × 10, 15 × 15, 10 × 20, 10 × 10 and 5 × 5 µm, respectively (see Table 1 ▸). In both Cristallina-MX experiments the loaded SOS chips were carried to the Cristallina-MX endstation, mounted on the diffractometer and diffraction images were acquired at 100 Hz using a JUNGFRAU 8M pixel detector. In rare cases of problems with the XFEL beam or other technical glitches the chips were stored in a humidity chamber as described previously (Gotthard *et al.*, 2024 ▸).

### 2.4. Data processing and analysis

All diffraction data were analyzed using *CrystFEL* 0.10.0, with *peakfinder*8 for peak finding and *XGANDALF* and *MOSFLM* for indexing (White *et al.*, 2012 ▸). Phasing was performed by molecular replacement using the structure with PDB code 6i43 as a search model. The structures were rebuilt and analyzed using *Coot* (Emsley & Cowtan, 2004 ▸; Emsley *et al.*, 2010 ▸) and refined with *REFMAC*5 (Murshudov *et al.*, 2011 ▸). To estimate errors for atomic coordinates and derived structural parameters such as torsion angles and interatomic distances, bootstrap resampling was performed as described in Gorel *et al.* (2021 ▸) and Grünbein *et al.* (2021 ▸). Briefly, for each data set, 100 statistically resampled versions were produced by applying a ‘drawing with replacement’ strategy to the set of images in the data set. The resampled sets of images were then independently processed and the resulting 100 MTZ files were used to refine 100 independent copies of each of the structures. These, in turn, were compared to determine standard deviations of the atomic coordinates. Before each of the 100 refinements the coordinates were ‘scrambled’ by stochastic displacement of the coordinates with a standard deviation of 0.3 Å to escape local minima.

Image drawing with replacement was implemented in a Python command-line tool that has been uploaded to GitHub (https://github.com/AlexanderGorel/bootstrap). Refinement was implemented by a custom processing pipeline using Bash and Python scripts and the command-line interfaces to *CCP*4 tools, and was parallelized for the SUN Grid Engine.

Since two different crystal batches were used to collect the Cristallina-MX (June 2023) and ID29 (February 2024) data, with slightly different unit-cell parameters, we worried about the significance of the overall differences in heme-coordination parameters. To assess this, we assumed, for simplicity, that for each parameter such as a bond length *etc.* the data points from each separate beam time are samples drawn from the same distribution (*i.e.* that there is no dependence of each parameter on translation step size). We then evaluated the probability that this distribution differs between the two beam times (ID29/Cristallina-MX). In order to do so, we needed to estimate the parameters of these overall distributions from the structural parameters and their standard deviations as estimated by bootstrapping. This was performed numerically by summing Gaussians with the respective mean values and variances of the various data sets from a beamtime and evaluating the standard deviation of the resulting overall distribution. (The custom Python script that performed this has been uploaded to GitHub with the bootstrapping scripts.) For each structural parameter, we then compared the distribution obtained from the ID29 data with that obtained from the Cristallina-MX data, and evaluated the significance of any differences by calculating a *p*-value. To take differences in the variances of the distributions into account, this was performed using Welch’s *t*-test (as implemented at https://www.graphpad.com/quickcalcs/ttest1/) rather than the independent *t*-test. The latter gives slightly smaller *p*-values.

Data and refinement statistics are shown in Supplementary Tables S1 and S2. The structures and data have been deposited in the PDB and at Zenodo (https://zenodo.org/records/15148393).

## 3. Results and discussion

The underlying cause of radiation damage is the inelastic interaction of X-ray photons with the sample, resulting in photoionization (and Compton scattering). The energy deposited in the sample relaxes via a cascade of ionization events and heating. Subsequent radiolytic reactions can result in bond breakage and other chemical reactions. When studying radiation damage, it is important to distinguish between global and local damage. Global damage concerns the entire diffracting volume and affects data quality by compromising the crystalline lattice. Thus, this type of damage typically manifests itself in increased *B* factors and/or reduced resolution. One type of global damage, Bragg termination, is particularly relevant to serial crystallography (Lomb *et al.*, 2011 ▸; Barty *et al.*, 2012 ▸). In Bragg termination, lattice disorder increases during a single exposure, leading to a gradual loss of diffraction intensity, starting at high resolution, as the diffraction takes place. This results in a change of the ratio of high- to low-resolution Bragg intensities and an increase in *B* factor during the exposure.

Local damage can be more difficult to detect and therefore arguably more insidious. Here, particular sites in the structure are affected, typically chemically reactive sites such as metal and other cofactors, typically through photoreduction. As these are often the focus of research, this can irretrievably compromise interpretations. A typical example is the photoreduction and concomitant spin change in heme proteins, which alter the coordination of the heme iron, often even at very low X-ray doses, as described, for example, in Schlichting *et al.* (2000 ▸), Berglund *et al.* (2002 ▸), Beitlich *et al.* (2007 ▸), Kühnel *et al.* (2007 ▸) and Pfanzagl *et al.* (2020 ▸). Mitigation of both types of radiation damage can be attempted by employing serial crystallography. However, radiation damage cannot be excluded in serial crystallography, even at XFELs (Nass *et al.*, 2015 ▸), and thus vigilance remains necessary to ensure data quality and avoid erroneous structural interpretations.

In serial crystallography (and rotation crystallography if the crystals are larger than the X-ray beam), radiation damage can occur either ‘directly’ during the exposure or ‘indirectly’ via ‘products’ of the absorbed energy. The ‘products’ can be heat (see, for example, Warren *et al.*, 2019 ▸), shockwaves (Grünbein *et al.*, 2021 ▸), radicals, reactive oxygen species and hydrogen created by radiation chemistry (Meents *et al.*, 2010 ▸) that may affect crystals (or parts thereof) that will be exposed at a later time during data collection. The damaging effects that these irradiation ‘products’ can cause are time- and distance-dependent. While the solid well boundaries of patterned fixed targets can isolate crystals from irradiation ‘products’, this is obviously not the case for unpatterned fixed targets such as the SOS chip. Due to the typical serpentine-like raster scans employed with fixed targets (see Fig. 1 ▸), the time between exposures within a line differs significantly from those in adjacent lines. The shortest time separation is that between adjacent exposures within a given line and the largest is that between the first exposure of one line and the last exposure of the subsequent line [Fig. 1 ▸(*c*)]. Clearly, both the intra-line and inter-line spacings influence whether or not radiation damage can occur ‘indirectly’.

**Figure 2:**
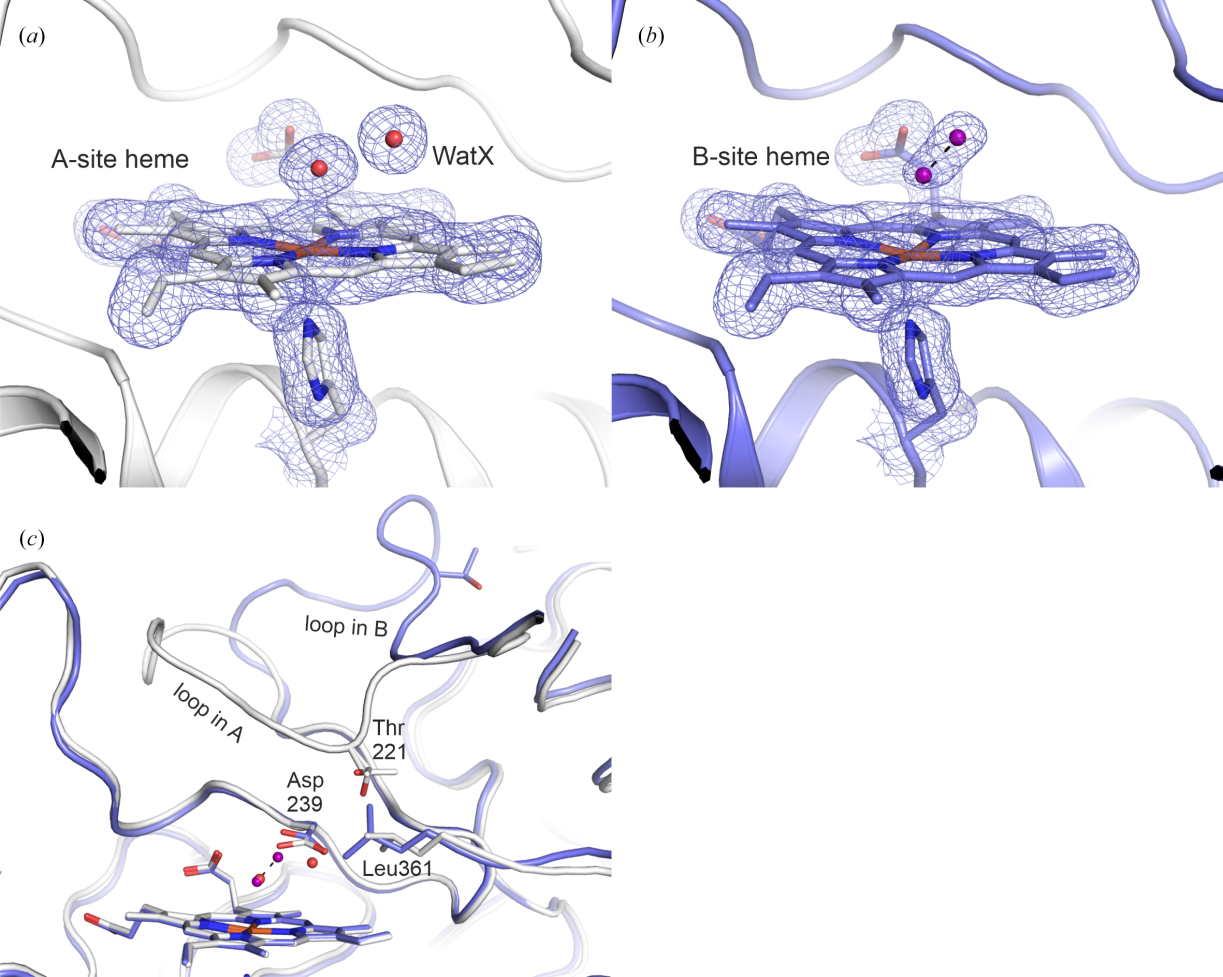
The active sites in the A and B molecules of the DtpAa dimer in the asymmetric unit differ. The A molecule contains a bound water molecule (*a*), while the B molecule contains either two very closely spaced (∼1.6–1.8 Å distance depending on the refinement procedure; see Supporting Information) water molecules (*b*) or a diatomic ligand (for example a hydroperoxo ligand). (*c*) The reason for this difference in heme coordination is the orientation of the amino-acid stretch 217–230 that either points towards the active site, affecting the orientation of the catalytic Asp239 and Leu361 (A molecule, gray), or towards the outside (B molecule, purple). (*a*) and (*b*) show 2*mF*_o_ − *DF*_c_ electron-density maps (blue mesh), contoured at 1σ, for the heme and heme-ligating molecules in the A and B molecules, respectively, calculated from the ID29 100 × 100 µm data. The maps are superimposed onto the final, refined structure.

Dye-decolourizing peroxidases (DyPs) are oxidative heme-containing enzymes that can oxidize organic substrates by first reacting with hydrogen peroxide to yield a catalytically active ferryl heme intermediate that results in electron transfer from substrates. DyPs differ from other nonmammalian peroxidases not only in their overall fold but also in their active-site structure. DyPs lack the distal heme-pocket histidine residue that is part of a highly conserved His–Arg couple in other peroxidases. In DyPs the role of the histidine as an acid–base catalyst in the proton-assisted heterolysis of the O—O bond of the bound peroxide substrate is taken over by an acidic residue, most commonly an aspartate (Sugano *et al.*, 2007 ▸). The A-type DyP DtpAa from *Streptomyces lividans* has become a well characterized model system to investigate the mechanism of DyPs using a variety of techniques. This enzyme displays a typical DyP active site [Figs. 2 ▸(*a*) and 2 ▸(*b*)], with a heme coordinated on the proximal side by a histidine, whereas a solvent molecule is bound at the distal side. Studies of the DtpAa mechanism have included X-ray crystallographic structure determination of defined redox states of the enzyme (Ebrahim *et al.*, 2019 ▸; Lučić *et al.*, 2020 ▸). This is challenging, because photoreduction during X-ray data collection can change the spin and redox state of the heme and thereby various associated structural parameters such as the position of the iron with respect to the heme plane. Moreover, it can affect the bond lengths from the heme iron to the proximal histidine and to the solvent molecule on the distal side. Complicating description and interpretation, the asymmetric unit of DtpAa crystals contains two molecules that show considerable differences in structure. The ‘A’ molecule appears to contain a small solvent molecule that is typically modeled as a water, but the density in the ‘B’ molecule is elongated to such an extent that a single water (or hydroxide) does not adequately explain it. For this reason, the active site of the B molecule has been largely ignored so far (see below).

The ligation of the heme iron in DtpAa is very sensitive to radiation damage. Indeed, it was reported previously that the bond between the distal water and the heme iron in DtpAa is significantly longer in structures determined by room-temperature SSX on beamline I24 at Diamond Light Source than in a structure obtained by SFX at SACLA, indicating radiation damage in the synchrotron data (Ebrahim *et al.*, 2019 ▸). (The authors only describe the active site in the A molecule.) Moreover, it was reported that the bond length increases linearly with (sequentially) absorbed dose in the SSX structures (Ebrahim *et al.*, 2019 ▸) and, consequently, this parameter was presented as a useful tool for the study of radiation damage. For both the SSX and SFX data collections in the study by Ebrahim and coworkers, patterned silicon chips were used. A comparison with SOS chips was accordingly of considerable interest. Our data from ID29 and Cristallina-MX were analyzed and refined using the same approach, determining the geometries of the active sites of the different structures to be examined in comparison with the published XFEL-derived structure (Ebrahim *et al.*, 2019 ▸).

To obtain an error estimate of the coordinates, including the XFEL-derived structures, we used bootstrapping (Gorel *et al.*, 2021 ▸; Grünbein *et al.*, 2021 ▸). This sets our study apart from most, if not all, other radiation-damage investigations, which typically do not evaluate the statistical significance of their findings. For the published XFEL data (Ebrahim *et al.*, 2019 ▸), error estimation involved returning to the original *CrystFEL* stream files (provided by R. Owen) to generate the resampled data sets. In the course of this we realized that a very large fraction of the diffraction intensities collected at SACLA are saturated [Supplementary Fig. S2(*a*)], resulting in a ‘clipping’ of the intensity values and thus a strong reduction of the modulation of the intensity distribution and the information content. This might be the reason for the unusual Wilson plot [Supplementary Fig. S2(*b*)] of these data. Our reprocessing of the SACLA stream files from Ebrahim and coworkers yielded results that differ from those derived from those deposited in the PDB [PDB entry 6i34; Supplementary Fig. S2(*b*)]. The reason for this discrepancy is unclear. Detailed information on data processing that might have clarified the issue is not provided in the publication (Ebrahim *et al.*, 2019 ▸). We collected XFEL data at SwissFEL, again employing the SOS chip, to allow a XFEL/synchrotron structural comparison.

### 3.1. Serial synchrotron data: the heme active site

It has been reported that the bond length between the heme iron and the bound water molecule is highly prone to radiation damage (Ebrahim *et al.*, 2019 ▸). It was thus interesting to find that for all step sizes within (Δ*x*) and between (Δ*y*) data-collection lines investigated at ID29 (see Table 1 ▸), the distance between the heme iron and the coordinating water molecule refined to the same value of ∼2.45 Å for the A molecule of the DtpAa dimer in the asymmetric unit [Figs. 2 ▸ and 3 ▸(*a*)]. This is very close to the value determined previously from the SACLA XFEL data (Ebrahim *et al.*, 2019 ▸), suggesting that no damage is present in the active site. Similarly, in the B molecule of the DtpAa dimer in the asymmetric unit (which had not been investigated previously) the electron-density features of the distal heme ligand appear to be independent of the data-collection step size [Figs. 2 ▸ and 3 ▸(*b*)]. Unexpectedly, while the electron density of the active site of the A molecule is clearly consistent with a water-coordinated heme [Fig. 2 ▸(*a*)], the electron density of the B molecule is in line with either a diatomic ligand or, far less likely, two water molecules, separated by ∼1.6–1.8 Å depending on the refinement parameters [Figs. 2 ▸(*b*) and 3 ▸(*c*); see Supplementary Note S1]. The bond angles obtained upon refinement further support the presence of a diatomic ligand. The Fe—O—O angle in the B site refines to ∼160°; if a water molecule hydrogen-bonded to another water were bound to the B-site heme, one would expect an angle much closer to 109.5° (tetrahedral coordination). Indeed, the Fe–Wat–WatX angle in the A site refines to ∼117° (WatX is the water molecule closest to the heme-bound water molecule).

**Figure 3:**
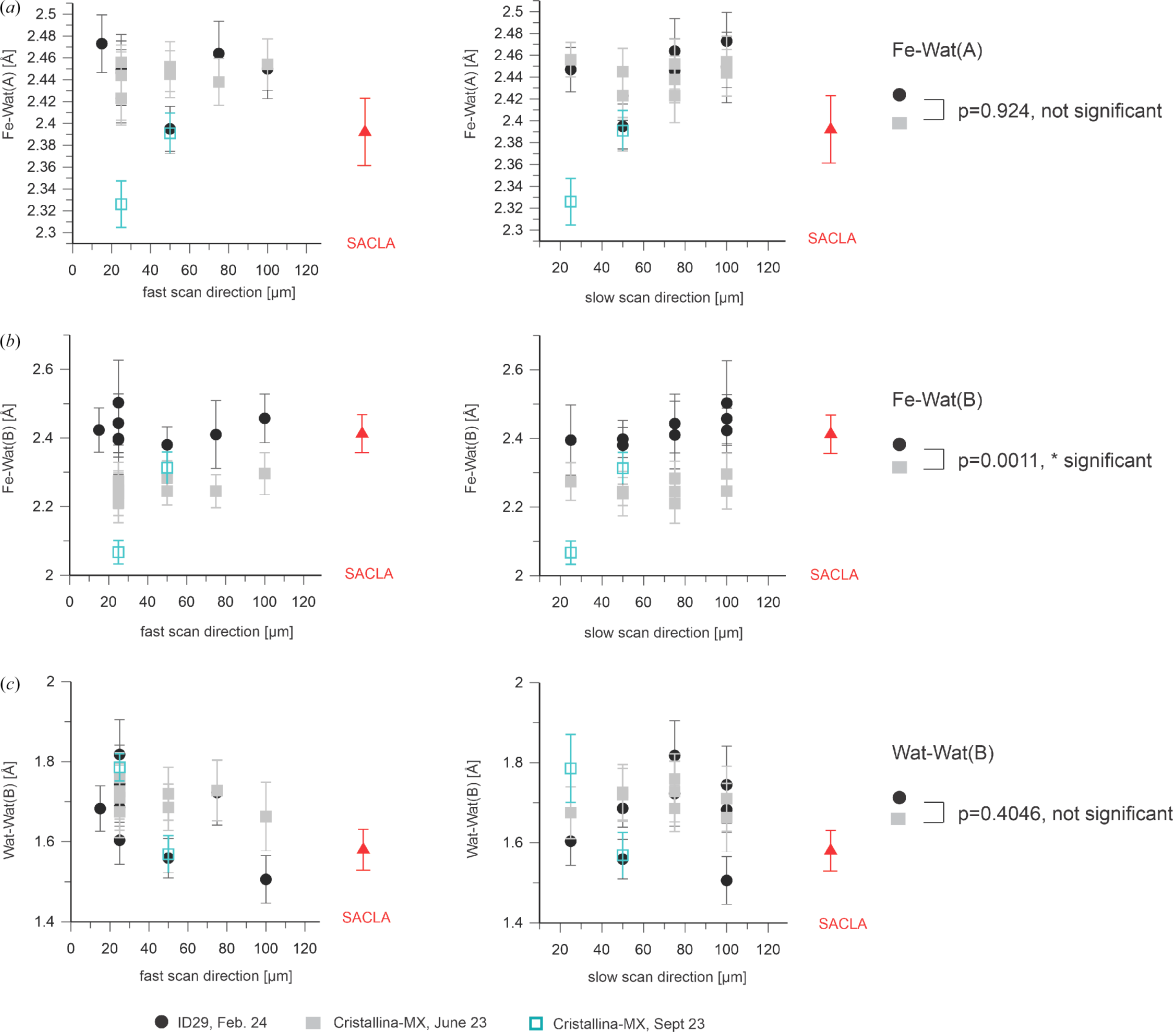
Distances of the heme water ligand [A and B molecules are shown in (*a*) and (*b*), respectively] and of the two O atoms in the distal heme ligand of the B molecule (*c*) as a function of the step size within a scan line (left) and between scan lines (right). ID29 data are shown in black (February 2024); Cristallina-MX data are depicted in gray (June 2023) and blue (September 2023). The SACLA data (red) show the results of the data collected by Ebrahim *et al.* (2019 ▸) after being reprocessed in this study. Error bars were derived by bootstrapping. The plotted values are listed in Supplementary Table S3. The Fe–Wat bond length derived from the February 2024 ID29 data for the B molecule is longer than that obtained from the Cristallina-MX-derived structures.

Strikingly, neither the iron–ligand bond length nor the distance between the two hypothetical water molecules (B molecule) changes significantly with the X-ray spacing (Fig. 3 ▸). In view of the ∼1.6–1.8 Å distance of the two atoms, we propose the ligand to be either a ferric hydroperoxo intermediate (Fe–OOH^−^, also referred to as Compound 0), in line with previous investigations on chloroperoxidase (Kühnel *et al.*, 2007 ▸), or a peroxo ligand (Nilsson *et al.*, 2004 ▸). Importantly, the structures of the A and B molecules in the DtpAa dimer in the asymmetric unit differ in the conformation of the amino-acid stretch made up of residues 217–230 [Fig. 2 ▸(*c*)], which either lines up with the central β-sheet covering the distal side of the heme in the A molecule or is a loop bulging out of the active site (B molecule). This latter conformation results in a missing van der Waals interaction of the A-Thr221 carbonyl with the catalytically essential A-Asp239. Thus, in the B conformation, Asp239 is not ‘pushed down’ but is slightly rotated (for mechanistic implications, see Yoshida *et al.*, 2011 ▸), thereby making room for a diatomic ligand and forming a hydrogen bond (2.5 Å) to the distal O atom of the presumed diatomic ligand. By contrast, in the A molecule A-Asp239 reaches further into the active site, where it forms a hydrogen bond (2.8 Å) to the water bound to the heme that would clash with a hydroperoxo ligand (2.1 Å to the distal O atom as observed in the B molecule). Moreover, in the absence of the 217–230 region in the active site the steric effect of A-Thr221 on A-Leu361 is missing, so that the latter moves into the active site. This prevents the binding of a water molecule in the B subunit that is observed in the A subunit and might correspond to the binding site of the water molecule generated upon cleavage of a bound hydroperoxo intermediate to yield a ferryl species. The conformation of the 217–230 region in the B molecule not only accommodates binding of a diatomic ligand but also generates a second tunnel reaching into the active site (Supplementary Fig. S3), potentially coordinating access of the organic substrate with hydrogen peroxide binding. In conclusion, since the B conformation is likely to resemble an active enzyme form whereas the A conformation corresponds to an inactive form, it is highly suggestive that the catalytic reaction involves large-scale conformational changes that open and close the active site. To the best of our knowledge, an induced-fit-like mechanism had not been suggested before.

**Figure 4:**
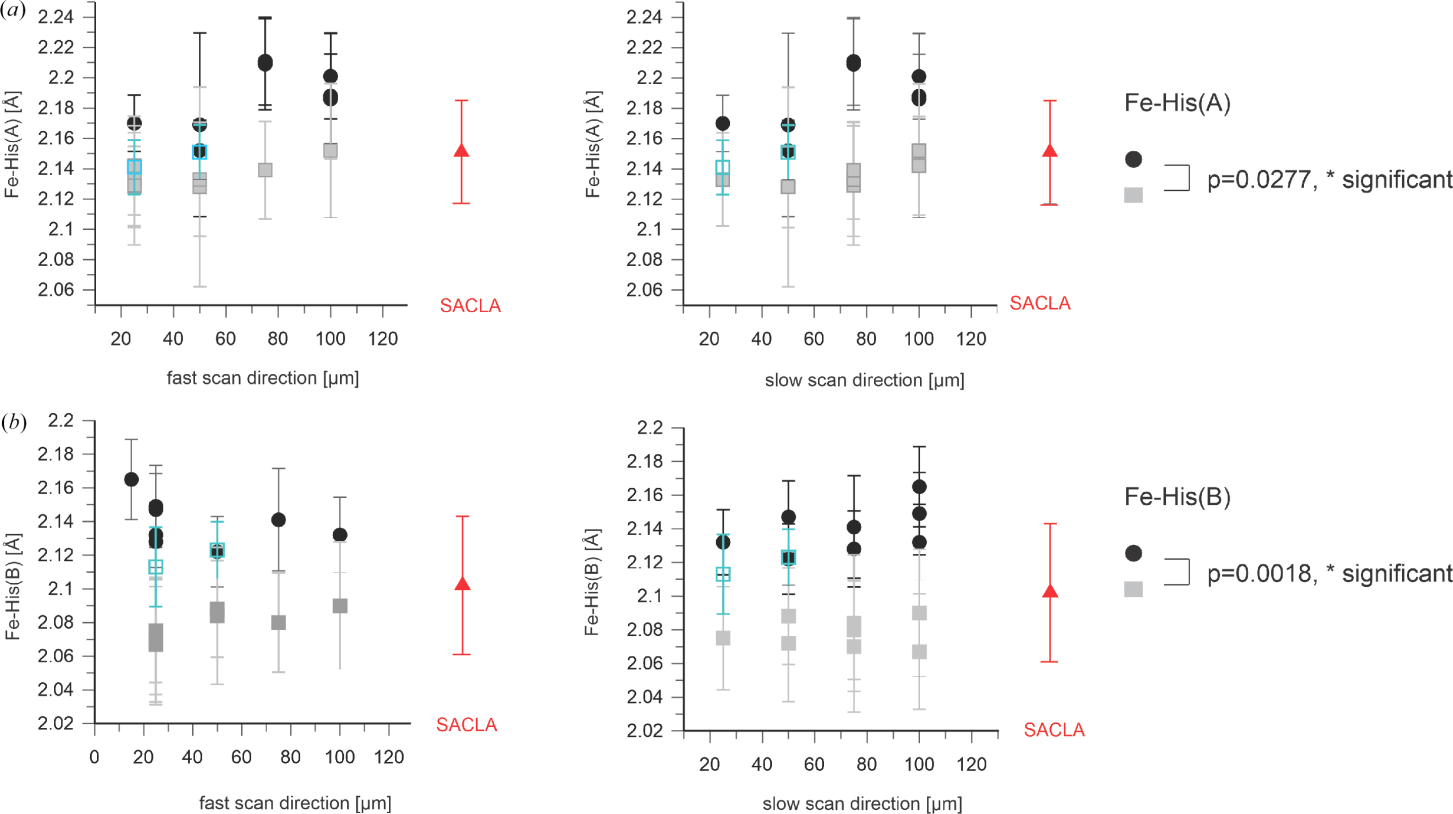
Length of the heme Fe–His bond [A and B molecules are shown in (*a*) and (*b*), respectively] as a function of the step size within a scan line (left) and between scan lines (right). ID29 data (February 2024) are shown as black circles; Cristallina-MX data are depicted in gray (June 2023) and blue (September 2023). The SACLA data (red) show the results of the data collected by Ebrahim *et al.* (2019 ▸) after being reprocessed in this study. Error bars were derived by bootstrapping. The plotted values are listed in Supplementary Table S3. The Fe–His bond length derived from the February 2024 ID29 data is longer than that obtained from the Cristallina-MX-derived structures. The effect is larger for the B molecule.

We next analyzed other geometric parameters of the heme as a function of the X-ray step size. As already mentioned, taking into account the scattering of the data, the Fe–Wat distance in the A and B monomers [Figs. 3 ▸(*a*) and 3 ▸(*b*)] seems to be independent of the scanning step size, as is the distance between the two O atoms in the diatomic ligand of the B monomer [Fig. 3 ▸(*c*)]. Similarly, the Fe–His bond length and the iron out-of-heme plane (FeOOP) displacement do not seem to change as a function of X-ray step size (Figs. 4 ▸ and 5 ▸).

### 3.2. Serial femtosecond crystallography data: global changes, heme active site

Similar to the ID29 data, we collected the SFX data during two beamtimes at Cristallina-MX (see Table 1 ▸). The crystals diffracted to significantly higher resolution: 1.54 Å (geometry-limited) compared with 1.67 Å at ID29 (not geometry-limited). During the first beamtime (June 2023), SFX data were collected using X-ray step sizes from 100 × 100 to 25 × 25 µm (see Table 1 ▸). The overall structural features of the two monomers of DtpAa in the asymmetric unit are essentially the same as described in Section 3.1 for the ID29 data concerning the distal heme ligand and the arrangement of the amino-acid region comprising residues 217–230. Importantly, no significant changes in hit rate, resolution or heme coordination parameters (Fe–His, Fe–Wat, FeOOP) were observed as a function of the X-ray step sizes Δ*x* and Δ*y* (slow and fast, respectively) for these step sizes of ≥25 µm. To explore whether damage occurs for distances of <25 µm between X-ray exposures, we collected further SFX data during a second beamtime in September 2023, using X-ray step sizes from 50 × 50 µm down to 5 × 5 µm (see Table 1 ▸) and a different crystal batch. The 50 × 50 and 25 × 25 µm data serve as common reference points between the two beamtimes.

In contrast to the measurement series performed at ID29 and in June 2023 at Cristallina-MX that display non-isomorphism between but not within data series (see Supplementary Tables S1 and S2), in the September 2023 data there were large clustered distributions of unit-cell constants (apart from the data collected with 50 × 50 and 5 × 5 µm X-ray spacings; see Supplementary Fig. S4) that enable the tracing of a transformation from a small ‘normal’ unit cell (*a* = 73.4, *b* = 68.8, *c* = 75.9 Å, β = 105.7°) into a larger one (*a* = 75.0, *b* = 68.4, *c* = 77.6 Å, β = 107.6°) (see Supplementary Fig. S4). The resolution of the diffraction patterns with the larger unit cell is significantly lower than those with smaller unit cells [Fig. 6 ▸(*a*)], an observation that appears independent of how ‘wet’ the mounted sample was. With decreasing X-ray spacing the fraction of patterns with the larger unit cell increases, while the resolution [Fig. 6 ▸(*b*)] and hit rate decrease (not shown). Interestingly, when closing the X-ray shutter intermittently during the raster scan of the SOS chip the small unit-cell crystals, and with them the initial higher resolution diffraction, returned (Supplementary Fig. S5), suggestive of a radiation-generated, likely diffusible ‘agent’ that affects the diffraction properties of crystals in the adjacent yet-to-be-exposed scan lines.

The short adjacent exposure distances not only affected global crystal parameters such as lattice constants and order (resolution), but also the structure of the crystallized molecules themselves. Interestingly, in the large unit-cell crystals the structure of the DtpAa B molecule has undergone a major rearrangement compared with the small unit-cell crystals: the amino-acid stretch of residues 217–230 is no longer located outside the heme-binding pocket but lines it, essentially taking up the same conformation as in the A molecule [see Fig. 2 ▸(*c*)]. Strikingly, the distal heme ligand also changed, from an apparently diatomic ligand to two clearly separated water molecules like in the A molecule [see Fig. 7 ▸(*c*)]. This finding supports our hypothesis that the ‘loop-out’ conformation corresponds to an active configuration, whereas the ‘loop-in’ structure represents an inactive configuration. Compared with the small unit-cell crystals, in the large unit-cell crystals the Fe–Wat bond length decreases very strongly in the A molecule to essentially the value found in the B molecule [but without a close-by second ligand atom; Fig. 7 ▸(*a*)]. In the B molecule, the Fe–Wat bond length decreases strongly for step sizes of ≤25 µm to a value that is only slightly larger than for the large unit-cell crystals [Fig. 7 ▸(*b*)]. For all cases we modeled a water molecule as the distal heme ligand since we do not know the chemical nature of the ligand in the structures determined from data collected with step sizes of ≤25 µm.

The iron–histidine bond distance seems rather independent of the X-ray exposure distance in the A molecule, but elongates slightly in the B molecule for shorter spacings in the small unit-cell crystals [Fig. 8 ▸(*a*)]. In line with a change of the chemical nature of the distal ligand in the B molecule in the large unit-cell crystals, a strong increase of the Fe–His bond lengths is also observed [Fig. 8 ▸(*b*)]. By contrast, only a small increase is apparent in the A molecule [Fig. 8 ▸(*a*)]. The FeOOP distance decreases slightly in the A molecule with decreasing X-ray spacing: significantly in the B molecule and much more in both cases in the large unit-cell crystals (Fig. 9 ▸).

### 3.3. Comparison of the SSX and SFX data

#### 3.3.1. The distal heme iron-water bond

It has been reported that the Fe–water bond in the A molecule in DtpAa microcrystals is highly radiation-sensitive (Ebrahim *et al.*, 2019 ▸). It was therefore somewhat unexpected to observe no changes in this bond length in any of our data sets. In principle, this could simply be due to the absence of damage or, alternatively, due to the large difference in exposure time, tens of milliseconds at I24 (Ebrahim *et al.*, 2019 ▸) versus 90 µs at ID29 (current work). The latter possibility prompted us to think about slow radiation-induced chemical processes. Obvious candidates are reactions involving diffusion of reactants, for example radicals, or fast processes followed by energetically unfavorable, and thus slow, structural changes. Photoreduction of the heme iron is fast and changes in water coordination should follow photoreduction rapidly. However, in DtpAa a radiation-induced movement of the heme water is likely to be slow since it forms hydrogen bonds to Arg342 (2.7 Å), Asp239 (2.9 Å) and WatX (2.7 Å), which in turn is held in place by a strong interaction with Asp239 (2.6 Å). Ebrahim and coworkers write that ‘the Fe–Wat bond length is shown to vary rapidly as a function of absorbed dose, with all room-temperature synchrotron structures exhibiting linear deformation of the active site compared with the XFEL structure’. Not mentioned is that the distance between the heme water and WatX decreases concomitantly from 2.7 Å in the SFX structure (PDB entry 6i43) to 2.0 Å in the 131 kGy SSX structure (PDB entry 6i8j). The latter distance is unreasonably short. Moreover, chemical bond lengths neither vary linearly nor continuously. In a sufficiently fast time-resolved experiment one might observe the change in position of the water molecule upon a change in heme redox state, but in static structures such as those presented by Ebrahim and coworkers one cannot. It is far more likely that what Ebrahim and coworkers observed is a mixture of two or more states (for a similar issue, see Chu *et al.*, 2000 ▸), the proportions of which change with X-ray dose: upon photoreduction the heme water moves to a new, more distant position. This, however, requires that the tightly bound WatX leaves, which takes time: apparently longer than the 90 µs exposure time at ID29. Indeed, downloading the electron-density maps from Ebrahim and coworkers from the Electron Density Server shows clear difference density for an alternative position for the heme-coordinating water molecule. We therefore re-refined the structures from Ebrahim and coworkers with the heme water molecule in two alternative locations, one coordinating the heme and one bound further away but still hydrogen-bonded to Arg342, while refining the occupancies of these two states [Fig. 10 ▸(*b*)]. This showed a clear decrease in the refined occupancy of the closely bound conformer with X-ray dose [Fig. 10 ▸(*a*)] as well as an increase in the occupancy of the more distantly bound water molecule, with the occupancy of WatX decreasing concomitantly. This provides a much more plausible model of how the active-site chemistry is affected by photoreduction.

#### 3.3.2. Radiation damage within an exposure

In the framework of experiments performed (i) on a new beamline at a fourth-generation synchrotron with a double-multilayer monochromator to boost flux and (ii) at a new XFEL endstation, we collected two sets of serial still diffraction data from two batches of crystals using JUNGFRAU detectors. Importantly, in all cases comparable numbers of photons were used per X-ray exposure, *i.e.* well within an order of magnitude (see Table 1 ▸). However, despite these similarities, the resolution of the ID29 data (1.67 Å, set by S/N = 1.0) was much lower than that of the Cristallina-MX data (1.54 Å, geometry-limited, S/N = 4 at the detector edge). In principle, this could be caused by differences in beamline and data-collection parameters such as the wavelength bandwidth Δλ/λ (1% at ID29 versus 0.3–0.5% at Cristallina-MX) and beam divergence [1.9 × 0.7 mrad (horizontal × vertical) at ID29 versus 156.7 × 97.7 µrad (horizontal × vertical) at Cristallina-MX] affecting spot broadening, the background noise and/or differences in exposure time (90 µs at ID29 versus ∼35 fs at Cristallina-MX). The differences in beamline parameters (Δλ/λ, divergence) affect the shape of the Bragg reflections, with the ID29 spots being broader and ‘streakier’. The degradation of resolution due to spot broadening depends on the background level. Together, this may explain the lower resolution of the ID29 data; however, radiation damage is also a possible cause.

The energy deposited into the sample during X-ray exposure causes ionization and heating. For femtosecond pulses the exposed sample rapidly turns into a plasma, resulting in destruction (Chapman *et al.*, 2014 ▸), which is visible as tiny holes in the microcrystals and the SOS sheets (Fig. 11 ▸). By contrast, according to the ‘basic model’ described by Warren *et al.* (2019 ▸), the calculated equilibrium temperature increase of a DtpA crystal exposed at ID29 exceeds 1500 K. Due to the limitations of the ‘basic model’ this increase is highly overestimated. Nevertheless, in view of the high absorbed dose (Table 1 ▸; Orlans *et al.*, 2025 ▸) a significant temperature increase is expected during X-ray exposure at ID29, likely affecting crystalline order and complicating the interpretation of time-resolved experiments.

Post-exposure examination of the ID29 chips by high-resolution optical microscopy revealed no obvious alterations in crystal shapes, sizes and distributions within the SOS layer. Disordering on submicrometre scales, however, is invisible to optical microscopy. It is conceivable that during the X-ray exposure of a crystal its temperature rises, inducing motion and thus disorder of the crystallized molecules, resulting in features akin to Bragg termination (Lomb *et al.*, 2011 ▸; Barty *et al.*, 2012 ▸). The effect on the Bragg intensities is a redistribution of high/low-resolution intensities and an increase in the Wilson *B* factor. While the former can be difficult to detect, the latter is not. A comparison of the ∼50 × ∼50 µm data collected at ID29 and at Cristallina-MX during two beamtimes from two different crystal batches shows that the Wilson *B* factors are the same for the respective beamline irrespective of crystal batch and experiment date (Supplementary Fig. S6), but differ strongly between the SSX and SFX data [25 Å^2^ (ID29) versus 14 Å^2^ (Cristallina-MX), see Fig. 12 ▸(*a*) and Supplementary Fig. S6]. However, using Wilson plots to judge the falloff of Bragg diffraction intensities with resolution may be complicated by the fact that Wilson plots based on observed intensities often show large nonlinear regions, caused by, for example, background effects that have nothing to do with Bragg diffraction. Moreover, comparing such plots requires that they are on the same scale. Wilson plots calculated from the calculated intensities after the final stage of refinement are not affected by these complications; they solely reflect the falloff of Bragg diffraction with resolution (as the atomic model only accounts for this and not for other effects), and modern refinement programs calculate *I*_calc_ on an absolute scale. *I*_calc_-based Wilson plots for the ID29 100 × 100 µm and the Cristallina-MX 100 × 100 µm data are shown in Fig. 12 ▸(*b*); a clear difference in the falloff of Bragg intensity with resolution is immediately apparent. For instance, at 1/*d*^2^ = 0.3, or 1.8 Å resolution, there is a difference in Bragg diffraction intensity of about an order of magnitude. This difference between the two data sets is also reflected in the atomic *B* factors after refinement (see Supplementary Table S2). These observations imply differences arising in the crystals during exposure, suggesting the occurrence of Bragg termination at ID29. This conclusion is further supported by the fact that the resolution-dependence of the intensity distribution of the 32.8 kGy SSX data collected at I24 (Ebrahim *et al.*, 2019 ▸) is highly similar to the Cristallina-MX data but differs significantly from the ID29 data [Fig. 12 ▸(*a*)].

**Figure 5:**
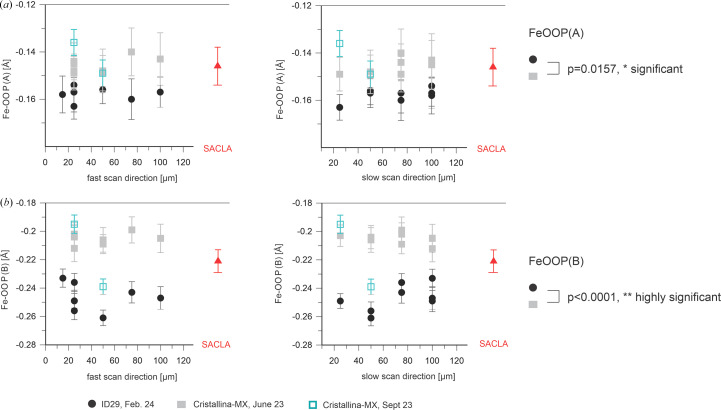
Iron-out-of-plane distance [A and B molecules are shown in (*a*) and (*b*), respectively] as a function of the step size within a scan line (left) and between scan lines (right). ID29 data are shown in black (February 2024); Cristallina-MX data are depicted in gray (June 2023) and blue (September 2023). The SACLA data (red) show the results of the data collected by Ebrahim *et al.* (2019 ▸) after being reprocessed in this study. Error bars were derived by bootstrapping. The plotted values are listed in Supplementary Table S3. The FeOOP distance is larger for the ID29-derived structures (black filled circles) than for those obtained from the Cristallina-MX data (gray filled squares, blue squares). The effect is larger for the B molecule.

In addition to these signs of global damage during exposure at ID29 apparently affecting the crystal order, there also appears to be local damage that changes structural features of the ligated heme (Figs. 3 ▸, 4 ▸ and 5 ▸). As depicted in Fig. 4 ▸, the Fe–His326 bond length is consistently longer in the ID29-derived structures than in the XFEL data-derived structures; this effect seems to be stronger for the B molecule. Similarly, in the B molecule the Fe–Wat distance seems to be longer in the ID29-derived structures than in the XFEL data-derived structures [Fig. 3 ▸(*b*)]. To check that these are not spurious effects due to a difference in unit-cell parameters, we refined the 100 × 100 µm ID29 data using the 100 × 100 µm Cristallina-MX cell and found a change of only 0.03 Å in either parameter, much smaller than the effects seen with the actual data sets. Thus, the observed differences in the structures are not due to differences in unit-cell parameters but are due to radiation damage. Moreover, we observe an increase in the FeOOP displacement for the ID29-derived structures compared with those from the XFEL data, which is also much larger for the B molecule than for the A molecule (Fig. 5 ▸). The different behavior of the A and B molecules is in line with the above-described observation of different distal ligands and supports the hypothesis that the B molecule does not contain a bound water molecule but a diatomic ligand. The significant increase in the values of the Fe–His bond length (Fig. 4 ▸) and decrease of the FeOOP distance (Fig. 5 ▸) between the XFEL- and the ID29-derived data suggests photoreduction and/or X-ray-induced chemical changes of the distal heme ligand (Nilsson *et al.*, 2004 ▸) during the 90 µs exposure time at ID29 [average diffraction weighted dose ∼0.88 MGy (see Table 1 ▸), dose rate ∼9.8 GGy s^−1^]. This is not unexpected in view of the reported half-dose of 40 kGy for iron(III) reduction in heme proteins (Pfanzagl *et al.*, 2020 ▸).

Interestingly, while the heme geometry differs between the ID29- and Cristallina-MX-derived structures, the coordination of the distal ligand in the A molecule is hardly altered. As pointed out above, we question the interpretation of Ebrahim *et al.* (2019 ▸), who report a chemically impossible continuous elongation of the Fe–Wat bond length (in the A molecule) with X-ray dose in the structures derived from data collected at I24 (Diamond Light Source) using multiple exposure times of 10 ms (dose ∼33 kGy, 3.3 MGy s^−1^; PDB entry 6i7z). Instead, we propose that upon photoreduction the distal heme water takes up a more distant position, which is linked to and strongly restricted by the movement of another tightly bound water molecule WatX. We conclude that the water movement is much slower than 90 µs, the exposure time at ID29, in contrast to heme iron reduction.

Besides changes in redox-active cofactors, modifications of disulfinde bridges are frequently cited radiation-damage effects. We observed no changes in the disulfide bridges in thaumatin structures (see Supplementary Fig. S7 and Doak *et al.*, 2024 ▸) derived from data collected in SOS chips at ID29 (average diffraction-weighted dose ∼1.47 MGy, dose rate ∼16.3 GGy s^−1^, 90 µs exposure) using, for example, Δ*x*, Δ*y* step sizes of 14 × 19 µm (Doak *et al.*, 2024 ▸). This is in line with findings by the ID29 beamline scientists on proteinase K (Orlans *et al.*, 2025 ▸), but differs from the observations on lysozyme described by de la Mora *et al.* (2020 ▸). Based on multiple room-temperature SSX data sets collected with dose rates of 2.4 and 40.3 MGy s^−1^ and 2.01 ms exposure time, the authors concluded that the half dose of disulfide damage is 0.08 MGy. They also suggested a half-dose limit of 0.38 MGy (the dose at which the diffracted intensity decreased to half of its initial value; *D*_1/2_) for a RT SSX data collection. While it is difficult to judge the extent to which the diffracted intensity may have decreased during the exposure at ID29, it is clear that our findings differ from the insights obtained from the first data set of dose-resolved SSX investigations (de la Mora *et al.*, 2020 ▸; Ebrahim *et al.*, 2019 ▸) using much lower doses and dose rates.

Previous investigations have come to differing conclusions on the importance of dose rate on radiation damage in room-temperature data collection (de la Mora *et al.*, 2020 ▸; Warkentin *et al.*, 2012 ▸, 2017 ▸; Southworth-Davies *et al.*, 2007 ▸). Nevertheless, it is clear that dose rate matters, as SFX at XFELs shows, albeit in the admittedly extreme case of diffraction before destruction at dose rates of 10^12^–10^13^ MGy s^−1^. Dose and dose rate determine the extent of ionization and heating, and the damage mechanisms. Radiation-damage-induced structural changes occurring on the femtosecond time scale at XFELs are governed by Coulomb forces (see, for example, Nass *et al.*, 2015 ▸, 2020 ▸). At synchrotron beamlines, data collection occurs on much longer time scales and consequently radiation damage also involves radiation chemistry and diffusive events. Thus, the specific manifestations of radiation damage can differ between data collections (and thus publications) since they not only depend on dose and dose rate, placing boundary conditions on relevant damage reaction times, but also the characteristics of the system investigated. Therefore, the concept of a generic ‘dose limit’ for all protein samples and experiment types to collect pristine (room-temperature) diffraction data at synchrotron sources (de la Mora *et al.*, 2020 ▸) is, in our opinion, an illusion.

#### 3.3.3. Radiation damage between exposures

**Figure 6:**
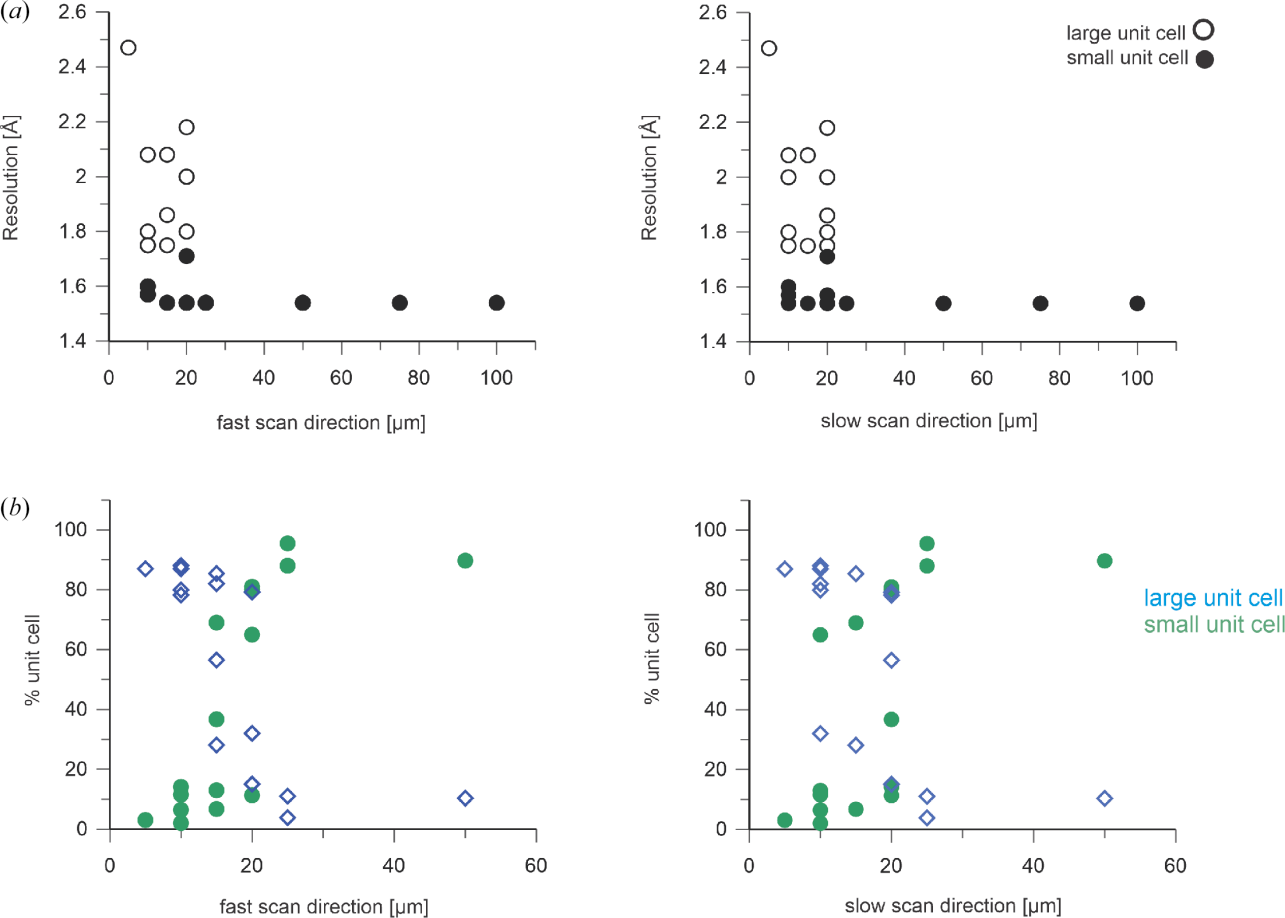
The resolution (*a*) and unit-cell (*b*) distribution of the DtpAa SFX diffraction data collected at Cristallina-MX in June and September 2023 depends on the step size between X-ray exposures of the SOS chips. For step sizes of ≥25 µm the data are dominated by crystals with the small, ‘normal’ unit cell diffracting to high resolution; for step sizes of <25 µm the contribution of crystals that have a larger unit cell and diffract to much lower resolution increases strongly. Data from the small unit-cell crystals (*a* = 73.4, *b* = 68.8, *c* = 75.9 Å, β = 105.7°) are depicted by filled symbols (black and green circles and the large unit-cell data (*a* = 75.0, *b* = 68.4, *c* = 77.6 Å, β = 107.6°) by open symbols (black circles and blue diamonds).

**Figure 7:**
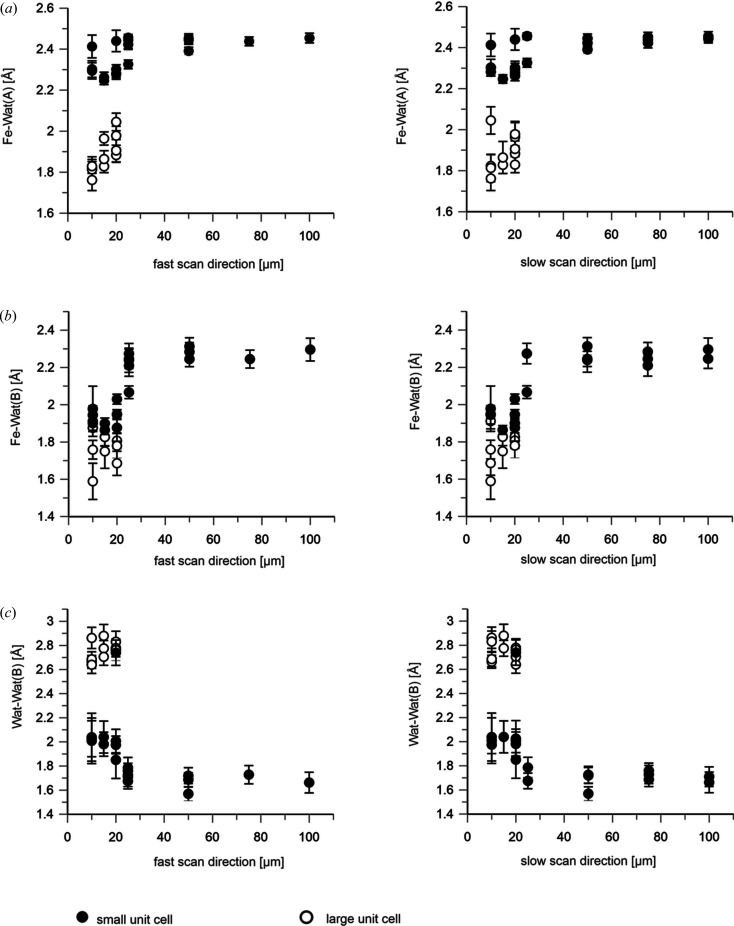
Distances of the heme water ligand [A and B molecules are shown in (*a*) and (*b*), respectively] and of the two O atoms in the distal heme ligand of the B molecule (*c*) derived from the DtpAa SFX diffraction data collected at Cristallina-MX in June and September 2023 as a function of the step size within (left) and between (right) scan lines. The plotted values are listed in Supplementary Table S3. Data from the small unit-cell crystals (*a* = 73.4, *b* = 68.8, *c* = 75.9 Å, β = 105.7°) are depicted by filled symbols (black circles) and the large unit-cell data (*a* = 75.0, *b* = 68.4, *c* = 77.6 Å, β = 107.6°) by open symbols (black circles). Error bars were derived by bootstrapping.

**Figure 8:**
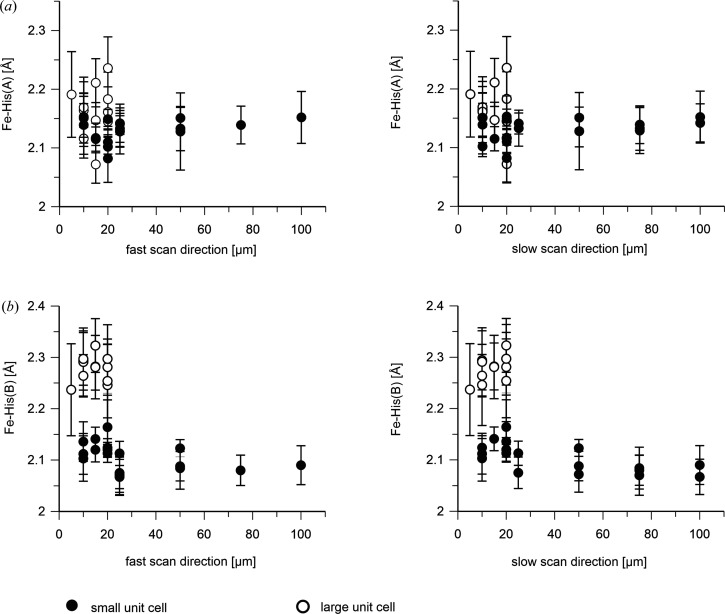
Bond length of the heme-proximal histidine ligand [A and B molecules are shown in (*a*) and (*b*), respectively] derived from the DtpAa SFX diffraction data collected at Cristallina-MX in June and September 2023 as a function of the step size within (left) and between (right) scan lines. The plotted values are listed in Supplementary Table S3. Data from the small unit-cell crystals (*a* = 73.4, *b* = 68.8, *c* = 75.9 Å, β = 105.7°) are depicted by filled symbols (black circles) and the large unit-cell data (*a* = 75.0, *b* = 68.4, *c* = 77.6 Å, β = 107.6°) by open symbols (black circles). Error bars were derived by bootstrapping.

**Figure 9:**
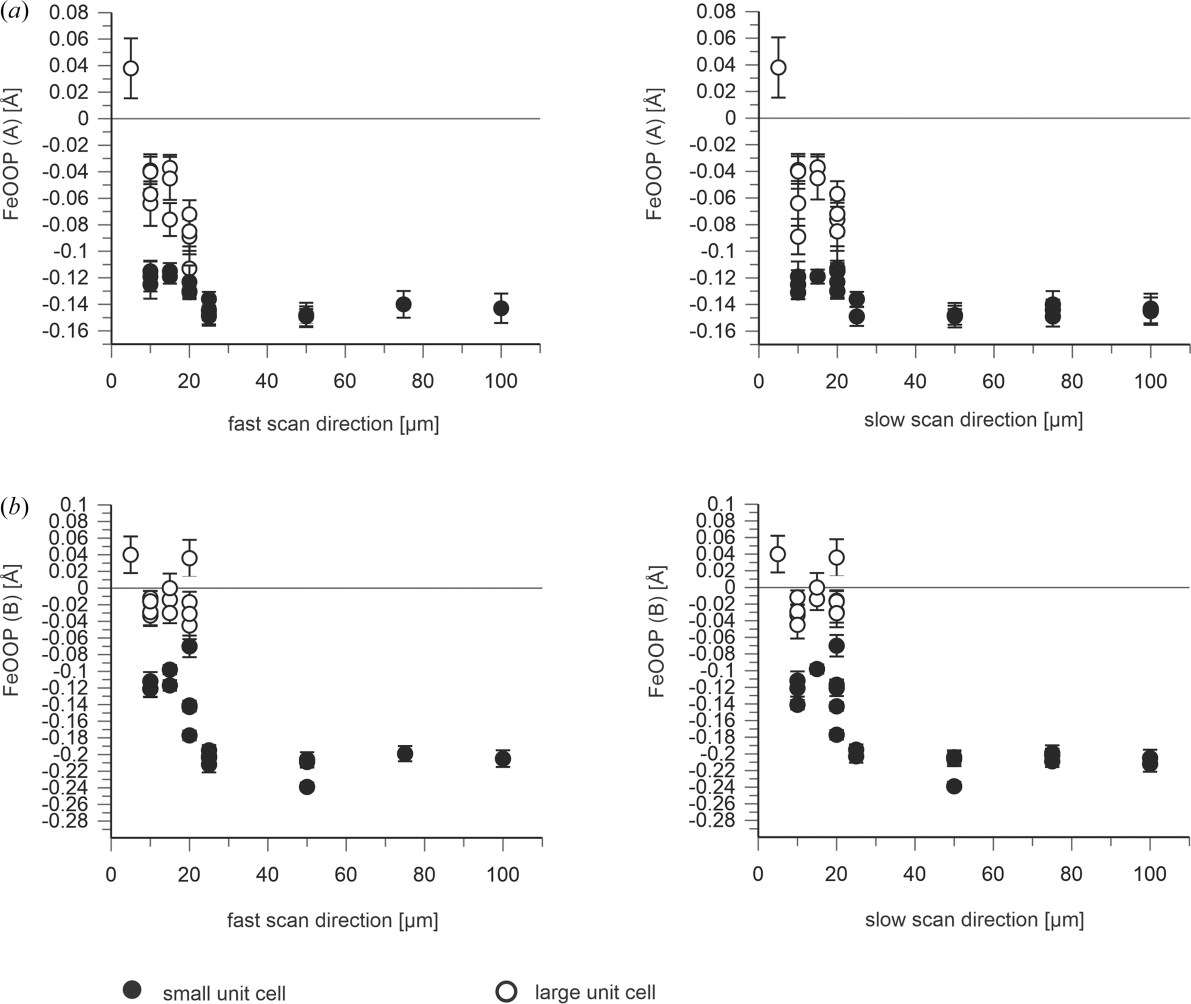
Iron out-of-plane distance [A and B molecules are shown in (*a*) and (*b*), respectively] derived from the DtpAa SFX diffraction data collected at Cristallina-MX in June and September 2023 as a function of the step size within (left) and between (right) scan lines. The plotted values are listed in Supplementary Table S3. Data from the small unit-cell crystals (*a* = 73.4, *b* = 68.8, *c* = 75.9 Å, β = 105.7°) are depicted by filled symbols (black circles) and the large unit-cell data (*a* = 75.0, *b* = 68.4, *c* = 77.6 Å, β = 107.6°) by open symbols (black circles). Error bars were derived by bootstrapping.

**Figure 10:**
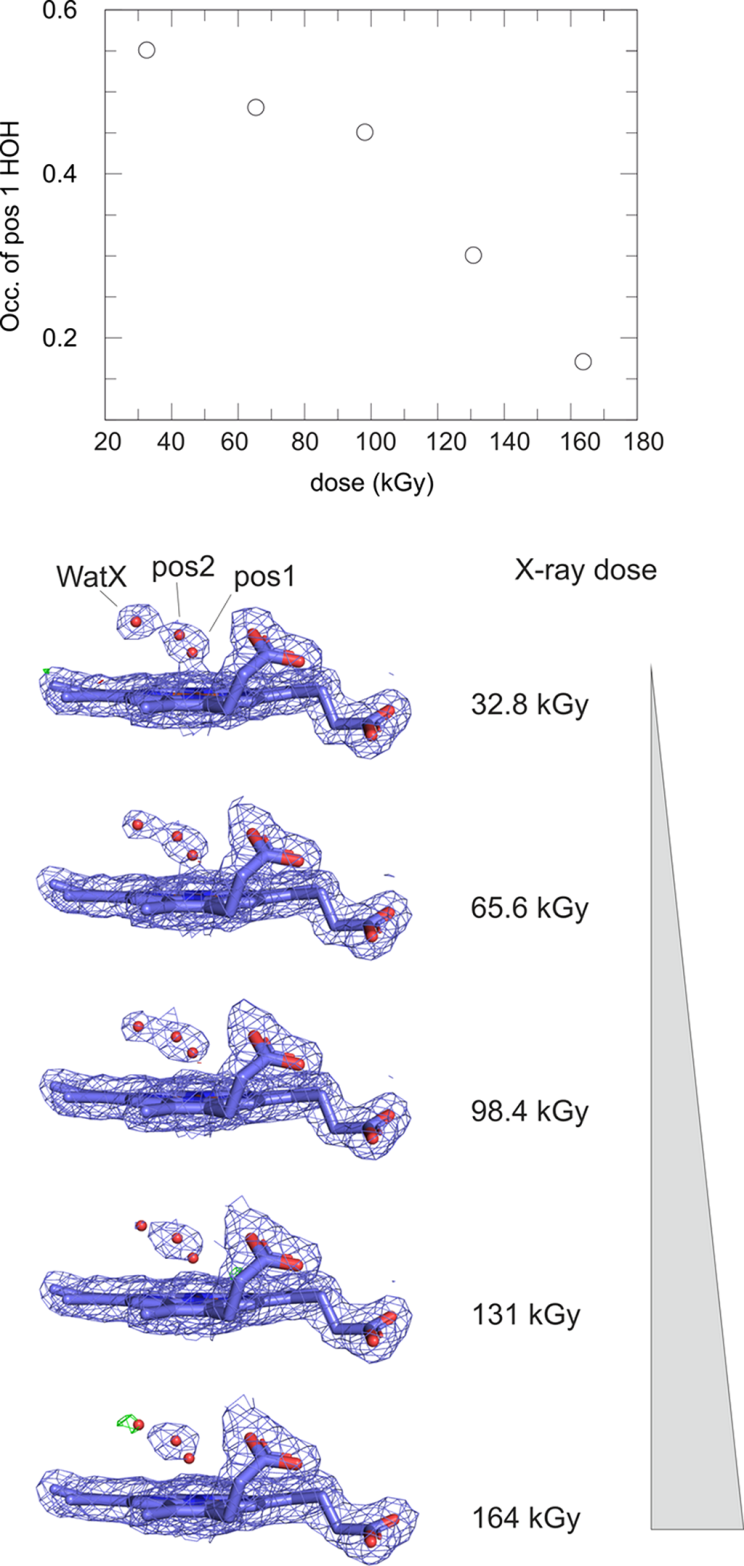
Reanalysis of the multiple low-dose SSX data sets (MSS1 series) published by Ebrahim *et al.* (2019 ▸). The authors described a linear dose-dependent migration of the heme-bound water molecule away from the heme Fe. An alternative, chemically more sensible interpretation is a relative occupancy change of two distinct water positions, the original one (pos1) that is decreasing with increasing dose and a new one further away from the iron (pos2) that increases with dose. The latter’s occupancy is linked inversely to the occupancy of WatX, which would clash sterically with the pos2 water molecule. Shown are 2*mF*_o_ − *DF*_c_ electron-density maps (blue mesh), contoured at 1σ, calculated from the data deposited in the PDB (Ebrahim *et al.*, 2019 ▸). Residual positive difference electron density is shown in green, contoured at 3σ. The maps are superimposed onto the final, refined structures.

**Figure 11:**
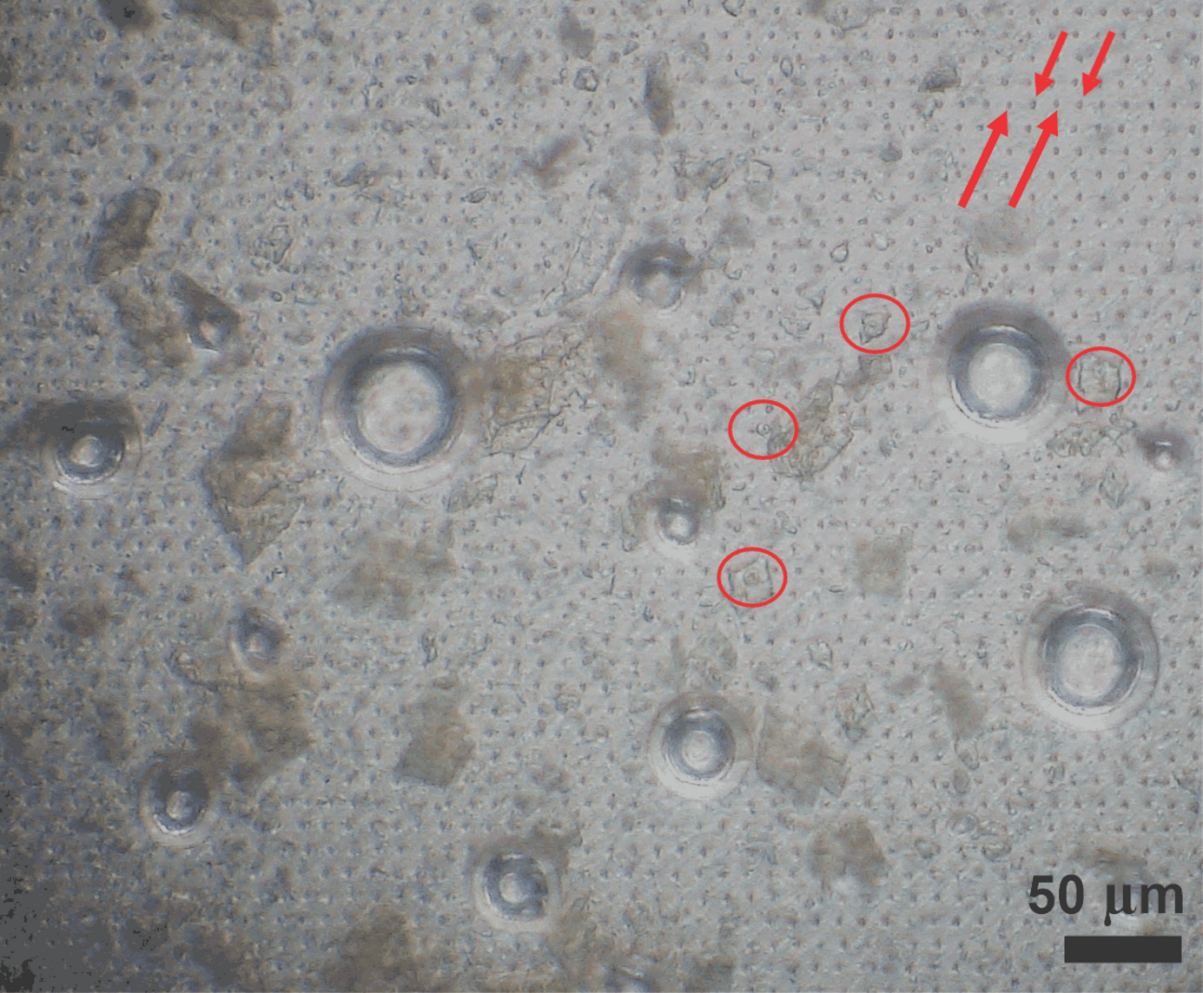
Micrograph of an SOS chip loaded with DtpAa microcrystals taken shortly after data collection at Cristallina-MX. The X-ray rastering step sizes were 10 × 10 µm within and between line scans, respectively. The regular array of small comma-shaped features (pointed out by red arrows) visible over the entire micrograph was created by the individual XFEL shots. Due to imperfect registry during the commissioning experiment of the scan translation stages (which in fact was identified in this way), these craters do not form a regular Cartesian array. This is of no major concern for SOS measurements (for a structured solid chip it would be dreadful). In addition to the regular array of craters there are some much larger circular features of varying sizes, possibly created by desiccation around certain anomalous holes punched by the XFEL beam or by the nucleation and growth of bubbles of gases generated by XFEL exposure. Moreover, several DtpAa crystals show a bulging torus (marked by red circles). We have often seen what appear to be ‘ejecta blankets’ where the XFEL beam has hit a crystal as opposed to liquid, and have associated this with a more violent interaction of the X-ray beam with the crystal matter than with the buffer liquid. The torus is next to but not around a neighboring small crater, possibly due to crystal or foil movement after X-ray data collection. The camera was focused on the upper SOS foil.

**Figure 12:**
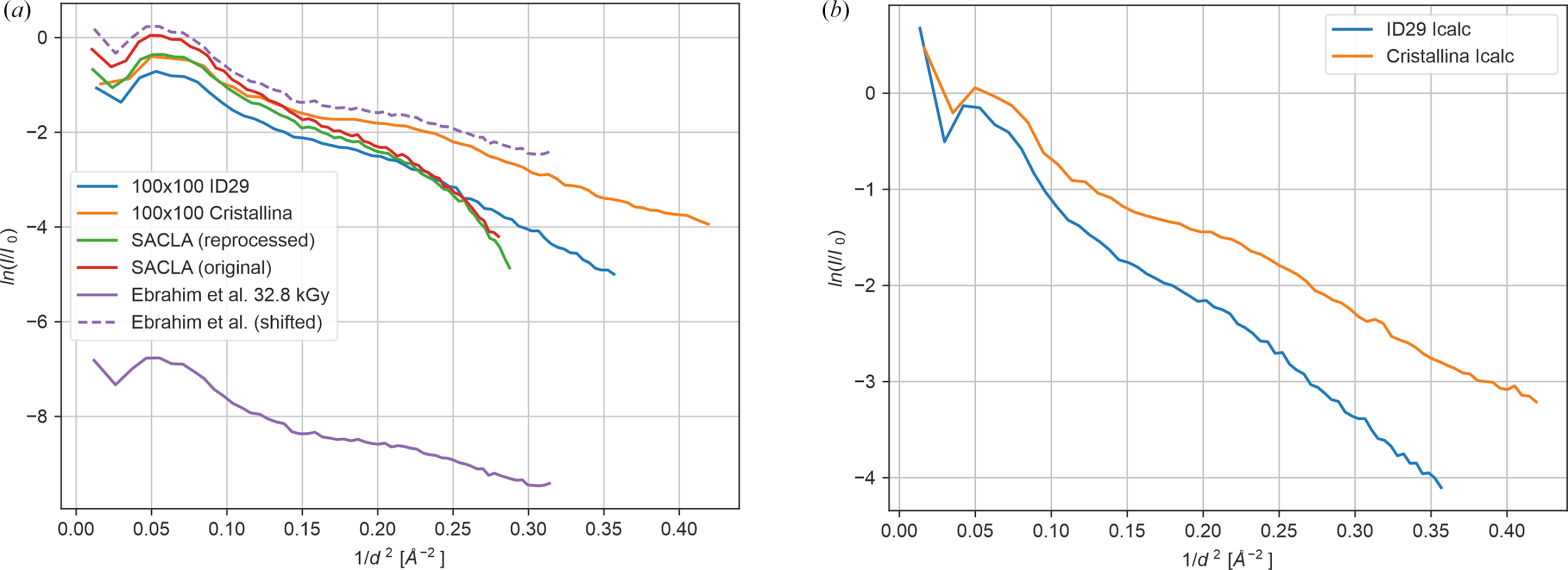
(*a*) Wilson plots of serial diffraction data from DtpAa microcrystals collected at SACLA by Ebrahim *et al.* (2019 ▸) using a patterned silicon chip as deposited (PDB entry 6i34, red line) and reprocessed by us (green line) and at SwissFEL (orange line) and ID29 (blue line) using an SOS chip and 100 × 100 µm (Δ*x*, Δ*y*) spacing between X-ray exposures. For comparison, the lowest dose SSX data published by Ebrahim *et al.* (2019 ▸) are shown in purple. They closely follow the Cristallina-MX data. By contrast, the high-resolution part of the 100 × 100 µm ID29 data falls off much more steeply, indicating differing diffraction properties. The underlying reason is not saturation of detector pixels (see Supplementary Fig. S6), but is most likely a radiation-damage process akin to Bragg termination. The slopes of the high-resolution data are essentially identical for 50 × 50 µm step size [see Supplementary Fig. S6(*b*)]. *I*_calc_-based Wilson plots are shown for the ID29 100 × 100 µm and the Cristallina-MX 100 × 100 µm step-size data (see text).

The energy deposited into the sample by the X-ray beam during exposure causes ionization and heating of the exposed material. In addition to changes in the sample occurring directly during the X-ray exposure, the X-ray-generated electrons, heat and subsequently formed free radicals, reactive oxygen species and hydrogen gas can propagate diffusively to affect portions of the sample well removed from the irradiation site. This is a matter of specific concern in SOS measurements. The mathematics and physics of such diffusion are well understood, allowing diffuse spreading to be accurately calculated provided that the relevant diffusion constants are known. They are not, in general, for the heterogeneous samples investigated in macromolecular crystallography. Nonetheless, calculations based on the physical properties of closely related materials deliver useful insight. Accordingly, calculations were undertaken to assess X-ray-induced thermal and gaseous diffusion under the conditions and geometry of these SOS measurements (Supporting Information). For the X-ray-induced temperature increase expected upon exposure at ID29 the calculations indicate that diffusive X-ray-induced thermal heating results in a temperature increase of a few degrees in neighboring crystals. This is possibly the origin of the observed small increase in Wilson *B* factors with decreasing step sizes between X-ray exposures (see Supplementary Fig. S8 and Table S1). The situation differs for the data obtained at Cristallina-MX. The power density of the focused XFEL beam is high enough to turn the exposed sample into a plasma, as shown by the physical holes burned through the crystals and SOS polymer films (Fig. 11 ▸). The calculations described in the Supporting Information allow an estimation of how fast and to what extent heat and hydrogen gas (Meents *et al.*, 2010 ▸) diffuse from an X-ray-irradiated spot to an adjacent, to be irradiated, spot. The temperature of the immediate area around the X-ray-exposed spot is unknown, but it seems unlikely that significant heating occurs on a 20 µm scale. It is unclear whether hydrogen gas bubbles form (Supplementary Fig. S5) and induce the phase transformation of the crystal lattice [small to large unit cell; see Fig. 6 ▸(*b*)] upon exposure of the SOS chip with X-ray step sizes of ≤20 µm (see the discussion in Supplementary Fig. S5). For very short spacings between X-ray exposures, an analysis of the orientation matrices of crystal hits showed that a significant fraction of the crystals were exposed more than once (*i.e.* different locations on the same crystal) for data collections with the very short spacings explored during the September 2023 (Cristallina-MX) beamtime (data not shown).

We consider it unlikely that the phase transition is caused per se by exposing crystals more than once, but instead is casued by dehydration of the crystal, most likely enhanced by water diffusion through the XFEL-punched through-holes (Fig. 11 ▸). However, multiple exposure of crystals may facilitate the diffusion of radical products to close by yet-to-be-exposed parts and may be the reason for the changes observed in heme parameters (Fe–His, Fe–Wat, FeOOP) for X-ray step sizes of ≤20 µm for the small unit-cell crystals.

Interestingly, the serendipitous small-to-large unit-cell phase transition of the DtpAa crystal lattice is associated with a large structural change in the B monomer in the asymmetric unit: the amino-acid stretch comprising residues 217–230 switches from the loop-out to the loop-in conformation (observed in the A monomer), affecting the orientation of the catalytic Aps239 and Leu361, and concomitantly transforming the diatomic ligand to a monomeric ligand. This observation supports the conclusions concerning the enzymatic mechanism of DtpAa described in Section 3.1.

## 4. Conclusions and outlook

For our radiation-damage study exploring the limits of SOS chips, we chose to use a well defined model system with a mechanistically relevant radiation-sensitive cofactor. Heme-containing peroxidases, with their high-valent oxygen intermediates, have been studied intensely, yet even obtaining a structure of the ferric resting state can be challenging due to X-ray-induced photoreduction. DtpAa thus seemed a perfect choice; moreover, it had been reported that the Fe–Wat bond length is highly radiation-sensitive (Ebrahim *et al.*, 2019 ▸). In the course of our investigations, it turned out that DtpAa is more complex than anticipated. We assigned the two structurally distinct monomers (A and B) in the asymmetric crystallographic unit as an inactive form (A molecule, displaying a water molecule bound to the heme iron) and an active conformation (B molecule, binding a hypothetical diatomic ligand, possibly a hydroperoxo species), in agreement with previous descriptions of the catalytic mechanism (Yoshida *et al.*, 2011 ▸). Importantly, a serendipitous phase transition of the crystals induced by dehydration shows that the B conformer transforms into the A conformer, with a concomitant change of the heme ligands (hypothetical diatomic ligand to water molecules). This observation implies that DtpAa uses an induced-fit mechanism.

In line with the functional difference between the A and B monomers, including the heme ligation, the two monomers react quite differently to X-ray exposure. Unexpectedly, however, neither shows the published elongation of the Fe–Wat bond. Revisiting the published structures (Ebrahim *et al.*, 2019 ▸), we reinterpreted the observations in a different, chemically plausible way involving changing ratios of two structurally distinct heme–water networks. Accordingly, the structural changes are slower than the 90 µs exposure time at ID29 and thus are not observable there. Thus, inadvertently but fortuitously, choosing DtpAa as the model system for our study of experimental limits in the use of SOS chips has allowed us to gain insights that clarify the radiation-damage mechanism as well as the enzymatic mechanism of DtpAa.

SOS chips are highly efficient tools for room-temperature SSX and SFX data collection, including the acquisition of serial diffraction data from extremely small crystals and from membrane-protein crystals grown in LCP, neither of which can be investigated efficiently using patterned chips. However, structured solid crystal-localization sites can have the advantage of shielding unexposed crystals from radicals or heat diffusing from previous exposure sites. By investigating the influence of the step size between X-ray exposures within and between scan lines, respectively, on the active-site geometry of a radiation-sensitive heme peroxidase, we show that step sizes of ≥25 µm do not yield manifestations of radiation damage in SFX-derived structures. We cannot exclude that the changes observed for smaller step sizes are caused by exposing crystals more than once.

The tens of microsecond-long, intense pink X-ray pulses at ID29 open a new regime of high-dose single-shot exposure data collection with dose rates on the order of 10^3^–10^4^ MGy s^−1^ (Orlans *et al.*, 2025 ▸) that remains as yet rather uncharacterized. By comparing SFX and SSX data and the derived respective DtpAa structures, we see indications that local and global damage occurs at ID29 during the 90 µs exposure. Follow-up experiments are needed to confirm this, systematically changing the X-ray pulse length and fluence. Among other things, this will allow the influence of the approximately fourfold higher dose absorbed during the ID29 exposure compared with that at Cristallina-MX on the integrated spot intensities and structural heme parameters to be established. The occurrence of global damage akin to Bragg termination can be tested by tracking the intensities of individual integrated Bragg reflections as well as of the overall integrated spot intensity distributions (Wilson plot) as a function of pulse length, similar to previous investigations at XFELs where single-shot ‘damage summation’ – the continuous addition of diffraction from increasingly damaged molecules – was first reported (Lomb *et al.*, 2011 ▸). Photoreduction, a specific form of radiation damage relevant to several enzymes classes likely to be studied at ID29 by TR-SSX, is often investigated by UV–Vis spectroscopy. This is, however, highly difficult for few micrometre-sized X-ray beams and crystals. Suitable model systems allowing the study of local radiation damage of biochemically relevant groups such as metal centers, clusters, flavins and other redox-sensitive cofactors will have to be identified and studied by systematically analyzing the effect of dose and dose rate on radiation-damage-sensitive features. This will provide much desired information about the timescales of the various damage processes.

The planned upgrade from an in-vacuum undulator (IVU21) to a cryogenic permanent magnet undulator (CPMU16), resulting in a decrease of the X-ray pulse length from 90 to 10 µs while keeping similar pulse energies, will open further possibilities for the exploration of dose-rate regimes at fourth-generation synchrotron sources that allow more radiation effects to be outrun than is possible at third-generation sources.

## 5. Related literature

The following references are cited in the supporting information for this article: Lučić *et al.* (2021 ▸) and Wang *et al.* (2023 ▸).

## Supplementary Material

PDB reference: DtpAa, 9gt0

PDB reference: 9gt1

PDB reference: 9gt2

PDB reference: 9gt3

PDB reference: 9gt4

PDB reference: 9gt5

PDB reference: 9gt6

PDB reference: 9gt7

PDB reference: 9gt8

PDB reference: 9gt9

PDB reference: 9gta

Supporting information. DOI: 10.1107/S2052252525008371/zf5027sup1.pdf

Excel file mentioned in the supporting information. DOI: 10.1107/S2052252525008371/zf5027sup2.xlsx

## Figures and Tables

**Table 1:** Synopsis of the different experiments

Beamline	Date	DtpAa sample	Sample medium HEC† (%)	Chip translation within/between lines, fast/slow‡ scan direction (µm)	Average No. of photons per pulse	Average diffraction-weighted dose§ (MGy)
Cristallina-MX	June 2023	Batch I	2.5	100 × 100, 75 × 75, 50 × 50, 25 × 25, 25 × 100, 50 × 75, 25 × 75, 25 × 50	5.44 × 10^10^	0.20¶
Cristallina-MX	September 2023	Batch II	2.0	50 × 50, 25 × 25, 20 × 20, 15 × 20, 10 × 20, 15 × 15, 20 × 10, 10 × 10, 5 × 5	6.22 × 10^10^	0.18
ID29	February 2024	Batch II	2.5	100 × 100, 75 × 75, 50 × 50, 25 × 25, 25 × 100, 25 × 75, 25 × 50, 15 × 100	4.90 × 10^10^	0.88

† HEC, hydroxyethyl cellulose.

‡ The scan axes are referred to as ‘fast’ for the principal translation direction (Δ*x* for ID29, Δ*y* for Cristallina-MX) and ‘slow’ for the secondary direction between scan lines (Δ*y* for ID29, Δ*x* for Cristallina-MX).

§ Dickerson *et al.* (2024 ▸). For the Cristallina-MX beamtime in June 2023 the XFEL pulse length was assumed to be 30 fs and it was known to be 25 fs in September 2023. The exposure time at ID29 was 90 µs.

¶ The average diffraction-weighted dose was ∼0.067 MGy for the 100 × 100 µm data set due to stronger attenuation of the X-ray beam.
