# Supplementary material for: Testing the limits: serial crystallography using unpatterned fixed targets
Source: IUCrJ. 2025 Oct 13;12(Pt 6):692–709. doi: 10.1107/S2052252525008371 (PMC12573926; doi:10.1107/S2052252525008371)
Supplement: Supplementary file 1 [file m-12-00692-sup1.pdf]

**IUCrJ**

**Volume 12 (2025)**

**Supporting information for article:**

# Testing the limits: serial crystallography using unpatterned fixed targets

**Alexander Gorel, Robert L. Shoeman, Elisabeth Hartmann, Stanislaw Nizinski, Martin V. Appleby, Emma V. Beale, Florian Dworkowski, Guillaume C. Gotthard, John H. Beale, James Holton, R. Bruce Doak, Thomas R. M. Barends and Ilme Schlichting**

**Table S1** Data collection and processing

ESRF Data; Values for the outer shell are given in parentheses.

| Data set | ID29, 100×100 | ID29, 75×75 | ID29, 50×50 | ID29, 25×100 | ID29, 25×75 | ID29, 25×50 | ID29, 25×25 | ID29, 15×100 | SwissFEL, 100×100 | SACLA* | SACLA reproc.** |
|---|---|---|---|---|---|---|---|---|---|---|---|
| pdb code | 9GT0 | 9GT1 | 9GT2 | 9GT3 | 9GT4 | 9GT5 | 9GT6 | 9GT7 | 9GT8 | 9GT9 | 9GTA |
| Diffraction source | ---- ESRF ID19 ---- | | | | | | | | Cristallina | SACLA | SACLA |
| Wavelength (Å) | ---- 1.073 ---- | | | | | | | | 1.030 | 1.24 | 1.24 |
| Temperature (K) | ---- 293 ---- | | | | | | | | 293 | 301 | 301 |
| Detector | ---- JUNGFRAU 4M ---- | | | | | | | | JUNGFRAU 8M | MPCCD | MPCCD |
| No. lattices | 105747 | 99311 | 220831 | 116706 | 216557 | 437285 | 1014316 | 258616 | 93572 | 73281 | 73281 |
| Space group | $P2_1$ | $P2_1$ | $P2_1$ | $P2_1$ | $P2_1$ | $P2_1$ | $P2_1$ | $P2_1$ | $P2_1$ | $P2_1$ | $P2_1$ |
| *a*, *b*, *c* (Å) | 73.6, 68.8, 75.5 | 73.6, 68.8, 75.5 | 73.6, 68.8, 75.5 | 73.6, 68.8, 75.5 | 73.6, 68.8, 75.5 | 73.6, 68.8, 75.5 | 73.6, 68.8, 75.6 | 73.6, 68.9, 75.6 | 72.6, 68.2, 73.9 | 72.7,68.2, 74.6 | 72.7,68.2, 74.6 |
| α, β, γ (°) | 90,105.8, 90 | 90,105.8, 90 | 90,105.8, 90 | 90,105.8, 90 | 90,105.8, 90 | 90,105.8, 90 | 90,105.8, 90 | 90,105.8, 90 | 90, 105.5, 90 | 90,105.6,90 | 90,105.6,90 |
| Resolution range (Å) | 40-1.67 (1.71-1.67) | 40-1.67 (1.71-1.67) | 40-1.60 (1.64-1.60) | 40-1.67 (1.71-1.67) | 40-1.67 (1.71-1.67) | 40-1.63 (1.67-1.63) | 40-1.57 (1.61-1.57) | 40-1.67 (1.71-1.67) | 40-1.54 (1.58-1.54) | 41-1.86 (1.91-1.88) | 40-1.86 (1.91-1.86) |
| Total No. of reflections | 58940541 | 54836305 | 145808605 | 61866083 | 108050720 | 248790476 | 631102166 | 128367709 | 65610029 | not indicated | 23197898 |
| No. of unique reflections | 84225 | 84225 | 95797 | 84347 | 84225 | 90551 | 101383 | 84472 | 102828 | 57351 | 59164 |
| Completeness (%) | 100 (100) | 100 (100) | 100 (100) | 100 (100) | 100 (100) | 100 (100) | 100 (100) | 100 (100) | 100 (100) | 100 (100) | 100 (100) |
| Redundancy | 700 (458) | 651 (426) | 1522 (819) | 734 (479) | 1283 (839) | 2748 (1733) | 6225 (2667) | 1520 (992) | 638 (412) | 381 (213) | 392 (250) |
| $\langle I/\sigma(I)\rangle$ | 7.0 (1.2) | 6.5 (1.1) | 11.3 (1.5) | 6.8 (1.1) | 8.2 (1.1) | 12.8 (1.6) | 18.3 (1.3) | 9.1 (1.2) | 5.6 (3.5) | 9.6 (1.7) | 7.8 (2.2) |
| $R_{split.}$ | 0.122 (1.140) | 0.138 (1.325) | 0.069 (0.865) | 0.124 (1.290) | 0.108 (1.370) | 0.065 (0.879) | 0.040 (1.112) | 0.077 (1.006) | 0.178 (0.287) | 0.072 (0.583) | 0.105 (0.416) |
| *CC1/2* | 0.977 (0.298) | 0.966 (0.261) | 0.992 (0.482) | 0.975 (0.265) | 0.985 (0.273) | 0.994 (0.426) | 0.998 (0.399) | 0.993 (0.441) | 0.952 (0.882) | 0.993 (0.722) | 0.983 (0.795) |
| *CC** | 0.994 (0.677) | 0.991 (0.644) | 0.998 (0.806) | 0.994 (0.648) | 0.996 (0.654) | 0.998 (0.773) | 1.0 (0.755) | 0.998 (0.782) | 0.988 (0.968) | 0.998 *** (0.916) | 0.996 (0.941) |

| | | | | | | | | | | | |
|---|---|---|---|---|---|---|---|---|---|---|---|
| Wilson B (Å$^2$) | 26.4 | 26.7 | 26.7 | 26.9 | 27.7 | 27.7 | 28.1 | 28.2 | 18.6 | not indicated | 32.0 |

* Re-refinement against data from 6i43 (Ebrahim *et al.*, 2019), re-upload of the original data from 6i43, note that statistics are reproduced from reference (Ebrahim *et al.*, 2019).

**Re-processing of original stream files from 6i43 (Ebrahim *et al.*, 2019), refinement against reprocessed data.

***Originally not indicated; calculated from reported *CC1/2* value.

SwissFEL June 2023 data; Values for the outer shell are given in parentheses.

| Data set | Cristallina 25x25 0.3T | Cristallina 50x25 | Cristallina 75x25 | Cristallina 100x25 | Cristallina 50x50 0.3T | Cristallina 75x50 | Cristallina 75x75 0.3T | Cristallina 100x100 |
|---|---|---|---|---|---|---|---|---|
| Diffraction source | ----Cristallina---- | | | | | | | |
| Wavelength (Å) | ----1.030---- | | | | | | | |
| Temperature (K) | ----293---- | | | | | | | |
| Detector | ----JUNGFRAU 8M---- | | | | | | | |
| No. lattices | 75545 | 204875 | 83479 | 70472 | 68602 | 87708 | 99648 | 93601 |
| Space group | $P2_1$ | $P2_1$ | $P2_1$ | $P2_1$ | $P2_1$ | $P2_1$ | $P2_1$ | $P2_1$ |
| *a*, *b*, *c* (Å) | 72.8, 68.2, 73.9 | 72.7, 68.3, 73.9 | 72.6, 68.3, 73.9 | 72.7, 68.3, 74.0 | 72.6, 68.2, 73.9 | 72.7, 68.3, 73.9 | 72.7, 68.3, 73.9 | 72.6,68.2, 73.9 |
| α, β, γ (°) | 90, 105.6, 90 | 90, 105.6, 90 | 90, 105.5, 90 | 90, 105.6, 90 | 90, 105.6, 90 | 90, 105.5, 90 | 90, 105.6, 90 | 90, 105.5, 90 |
| Resolution range (Å) | 40-1.54 (1.58-1.54) | 40-1.39 (1.43-1.39) | 40-1.44 (148-1.44) | 40-1.44 (1.48-1.44) | 40-1.54 (1.58-1.54) | 40-1.44 (1.48-1.44) | 40-1.54 (1.58-1.54) | 40-1.41 (1.45-1.41) |
| Total No. of reflections | 60907844 | 191785803 | 66841190 | 58666432 | 71696112 | 69790249 | 112667280 | 72533980 |
| No. of unique reflections | 102780 | 139942 | 125824 | 126080 | 102780 | 125997 | 103056 | 133807 |
| Completeness (%) | 100 (100) | 100 (100) | 100 (100) | 100 (100) | 100.0 (100.0) | 100.0 (100.0) | 100.0 (100.0) | 100.0 (100.0) |
| Redundancy | 592.6 (381.1) | 1370.5 (194.5) | 531.2 (154.0) | 465.3 (134.9) | 697.6 (449.8) | 553.9 (160.5) | 1093.3 (705.4) | 542.1 (105.2) |
| ⟨ $I/\sigma(I)$⟩ | 5.6 (3.8) | 7.4 (1.3) | 5.0 (1.5) | 4.7 (1.5) | 6.7 (4.8) | 4.9 (1.6) | 7.9 (5.3) | 4.7 (1.1) |
| $R_{split.}$ | 0.177 (0.248) | 0.102 (0.779) | 0.168 (0.655) | 0.181 (0.657) | 0.156 (0.196) | 0.172 (0.636) | 0.124 (0.181) | 0.181 (0.925) |
| *CC1/2* | 0.954 (0.905) | 0.986 (0.571) | 0.961 (0.649) | 0.954 (0.64) | 0.963 (0.929) | 0.96 (0.655) | 0.977 (0.945) | 0.953 (0.476) |
| *CC** | 0.988 (0.975) | 0.997 (0.852) | 0.99 (0.887) | 0.988 (0.884) | 0.99 (0.981) | 0.99 (0.89) | 0.994 (0.986) | 0.988 (0.803) |
| Wilson B (Å$^2$) | 18.7 | 18.6 | 18.6 | 18.8 | 18.8 | 18.7 | 18.9 | 18.4 |

SwissFEL Data continued, September 2023; Values for the outer shell are given in parentheses.

| Data set | Cristallina 10x10 small ds1 | Cristallina 10x10_small ds2 | Cristallina 20x10_small | Cristallina 15x15 small | Cristallina 20x15 small | Cristallina 10x20 small | Cristallina 20x20 small | Cristallina 20x20 small ds1 | Cristallina 20x20 small ds2 | Cristallina 25x25 small | Cristallina 50x50 small |
|---|---|---|---|---|---|---|---|---|---|---|---|
| Diffraction source | ----Cristallina---- | | | | | | | | | | |
| Wavelength (Å) | ----1.030---- | | | | | | | | | | |
| Temperature (K) | ----293---- | | | | | | | | | | |
| Detector | ----JUNGFRAU 8M---- | | | | | | | | | | |
| No. lattices | 7408 | 5898 | 7533 | 58974 | 30703 | 77159 | 4380 | 55412 | 43846 | 96571 | 57558 |
| Space group | $P2_1$ | $P2_1$ | $P2_1$ | $P2_1$ | $P2_1$ | $P2_1$ | $P2_1$ | $P2_1$ | $P2_1$ | $P2_1$ | $P2_1$ |
| *a*, *b*, *c* (Å) | 73.4, 68.8, 75.9 | 73.4, 68.8, 75.9 | 73.4, 68.7, 75.9 | 73.2, 68.8, 75.9 | 73.2, 68.7, 75.9 | 73.3, 68.8, 75.9 | 73.3 68.7 76.0 | 73.3, 68.8, 75.9 | 73.4, 68.8, 75.9 | 73.4, 68.8, 75.8 | 73.5, 68.8, 75.6 |
| α, β, γ (°) | 90, 105.7 90 | 90, 105.7, 90 | 90, 105.7, 90 | 90, 105.5, 90 | 90, 105.6, 90 | 90, 105.6, 90 | 90, 105.7, 90 | 90, 105.6, 90 | 90, 105.6, 90 | 90, 105.6, 90 | 90, 105.7, 90 |
| Resolution range (Å) | 40-1.57 (1.61-1.57) | 40-1.60 (1.64-1.60) | 40-1.57 (1.61-1.57) | 40-1.54 (1.58-1.54) | 40-1.54 (1.58-1.54) | 40-1.54 (1.58-1.54) | 40-1.71 (1.76-1.71) | 40-1.54 (1.58-1.54) | 40-1.54 (1.58-1.54) | 40-1.54 (1.58-1.54) | 40-1.54 (1.58-1.54) |
| Total No. of reflections | 16013753 | 12985978 | 16643535 | 93693754 | 62060741 | 142281289 | 9026427 | 95764435 | 63279371 | 132693342 | 37201874 |
| No. of unique reflections | 101584 | 95863 | 101433 | 107424 | 107361 | 107510 | 78525 | 107510 | 107658 | 107505 | 107338 |
| Completeness (%) | 100.0 (100.0) | 100.0 (100.0) | 100.0 (100.0) | 100.0 (100.0) | 100.0 (100.0) | 100.0 (100.0) | 99.9 (99.8) | 100.0 (100.0) | 100.0 (100.0) | 100.0 (100.0) | 100.0 (100.0) |
| Redundancy | 157.6 (91.7) | 135.5 (77.0) | 164.1 (95.5) | 872.2 (525.9) | 578.1 (347.4) | 1323.4 (796.6) | 114.9 (74.4) | 890.7 (536.1) | 587.8 (353.5) | 1234.3 (742.0) | 346.6 (208.4) |
| ⟨ $I/\sigma(I)$⟩ | 3.6 (1.2) | 3.3 (1.1) | 3.6 (1.2) | 8.6 (2.9) | 6.8 (2.2) | 10.0 (3.3) | 3.2 (1.4) | 8.1 (2.8) | 6.6 (2.6) | 9.3 (3.8) | 6.0 (3.9) |
| $R_{split.}$ | 0.298 (1.030) | 0.361 (1.100) | 0.299 (1.035) | 0.119 (0.394) | 0.144 (0.521) | 0.103 (0.347) | 0.413 (1.400) | 0.138 (0.431) | 0.164 (0.424) | 0.118 (0.293) | 0.211 (0.270 |
| *CC1/2* | 0.803 (0.359) | 0.7 (0.284) | 0.805 (0.322) | 0.969 (0.791) | 0.956 (0.687) | 0.976 (0.826) | 0.608 (0.322) | 0.957 (0.738) | 0.945 (0.745) | 0.97 (0.863) | 0.908 (0.872) |
| *CC** | 0.944 (0.727) | 0.908 (0.665) | 0.945 (0.698) | 0.992 (0.94) | 0.989 (0.903) | 0.994 (0.951) | 0.869 (0.698) | 0.989 (0.921) | 0.986 (0.924) | 0.992 (0.963) | 0.976 (0.965) |
| Wilson B (Å$^2$) | 21.6 | 21.8 | 21.6 | 21.2 | 21.3 | 21.3 | 22.3 | 20.8 | 20.3 | 20.0 | 18.4 |

SwissFEL Data – continued September 2023; Values for the outer shell are given in parentheses.

| Data set | Cristallina 5x5 big | Cristallina 10x10 big ds1 | Cristallina 10x10 big ds2 | Cristallina 10x10 big ds3 | Cristallina 20x10 big | Cristallina 15x15 big ds1 | Cristallina 15x15 big ds2 | Cristallina 20x15 big | Cristallina 10x20 big | Cristallina 20x20 big ds1 | Cristallina 20x20 big ds2 | Cristallina 20x20 big ds3 | Cristallina 25x25 big |
|---|---|---|---|---|---|---|---|---|---|---|---|---|---|
| Diffr. source | ----Cristallina---- | | | | | | | | | | | | |
| Wavelength (Å) | ----1.030---- | | | | | | | | | | | | |
| Temp. (K) | ----293---- | | | | | | | | | | | | |
| Detector | ----JUNGFRAU 8M---- | | | | | | | | | | | | |
| No. lattices | 8839 | 101598 | 45980 | 20875 | 49059 | 44688 | 24515 | 44164 | 31460 | 37324 | 30190 | 8273 | 3880 |
| Space group | $P2_1$ | $P2_1$ | $P2_1$ | $P2_1$ | $P2_1$ | $P2_1$ | $P2_1$ | $P2_1$ | $P2_1$ | $P2_1$ | $P2_1$ | $P2_1$ | $P2_1$ |
| *a*, *b*, *c* (Å) | 75.1, 68.5, 77.5 | 75.0, 68.4, 77.6 | 75.0, 68.4, 77.6 | 75.0, 68.5, 77.5 | 75.0, 68.4, 77.6 | 75.0, 68.4, 77.6 | 75.0, 68.4, 77.6 | 75.0, 68.3, 77.6 | 75.0, 68.4, 77.6 | 75.0, 68.3, 77.6 | 75.0, 68.5, 77.6 | 75.0, 68.4, 77.6 | 75.0, 68.4, 77.6 |
| α, β, γ (°) | 90, 107.6, 90 | 90, 107.7, 90 | 90, 107.7, 90 | 90, 107.6, 90 | 90, 107.7, 90 | 90, 107.7, 90 | 90, 107.6, 90 | 90, 107.7, 90 | 90, 107.7, 90 | 90, 107.7, 90 | 90, 107.7, 90 | 90, 107.7, 90 | 90, 107.7, 90 |
| Resolution range (Å) | 40-2.47 (2.54-2.47) | 40-1.75 (1.80-1.75) | 40-1.80 (1.85-1.80) | 40-2.08 (2.13-2.08) | 40-1.75 (1.80-1.75) | 40-1.75 (1.80-1.75) | 40-2.08 (2.13-2.08) | 40-1.86 (1.91-1.86) | 40-2.00 (2.05-2.00) | 40-1.80 (1.85-1.80) | 40-2.00 (2.05-2.00) | 40-2.18 (2.24-2.18) | 40-2.69 (2.76-2.69) |
| Total No. of reflections | 2062090 | 74622931 | 31296489 | 6725581 | 37150637 | 26052109 | 10250214 | 28176611 | 17708361 | 21899527 | 14827547 | 3232378 | 743388 |
| No. of unique reflections | 27138 | 75532 | 69447 | 45176 | 75532 | 75532 | 45183 | 62935 | 50756 | 69366 | 50756 | 39280 | 20999 |
| Completeness (%) | 100.0 (100.0) | 100.0 (100.0) | 100.0 (100.0) | 100.0 (100.0) | 100.0 (100.0) | 100.0 (100.0) | 100.0 (100.0) | 100.0 (100.0) | 100.0 (100.0) | 100.0 (100.0) | 100.0 (100.0) | 100.0 (100.0) | 99.9 (99.9) |
| Redundancy | 76.0 (50.7) | 988.0 (673.9) | 450.7 (318.1) | 148.9 (104.4) | 491.9 (335.4) | 344.9 (235.6) | 226.9 (159.8) | 447.7 (316.4) | 348.9 (244.1) | 315.7 (222.5) | 292.1 (204.2) | 82.3 (57.0) | 35.4 (21.6) |
| ⟨ $I/\sigma(I)$⟩ | 3.4 (1.4) | 8.4 (1.3) | 5.9 (1.2) | 3.7 (1.5) | 5.8 (0.9) | 5.3 (1.1) | 4.4 (2.0) | 5.4 (1.2) | 5.1 (1.2) | 4.8 (1.1) | 4.6 (1.3) | 2.9 (1.2) | 2.5 (1.2) |
| $R_{split.}$ | 0.259 (0.778) | 0.084 (0.931) | 0.131 (1.158) | 0.204 (0.735) | 0.129 (1.361) | 0.160 (1.210) | 0.192 (0.574) | 0.139 (0.979) | 0.160 (1.026) | 0.182 (1.184) | 18.8 (98.8) | 0.321 (0.954) | 0.447 (0.921) |
| *CC1/2* | 0.89 (0.633) | 0.988 (0.651) | 0.969 (0.445) | 0.944 (0.545) | 0.971 (0.477) | 0.953 (0.498) | 0.936 (0.613) | 0.968 (0.591) | 0.955 (0.528) | 0.933 (0.416) | 0.943 (0.535) | 0.814 (0.444) | 0.583 (0.382) |
| *CC** | 0.971 (0.881) | 0.997 (0.888) | 0.992 (0.785) | 0.985 (0.84) | 0.993 (0.803) | 0.988 (0.815) | 0.983 (0.872) | 0.992 (0.862) | 0.988 (0.831) | 0.983 (0.766) | 0.985 (0.835) | 0.947 (0.784) | 0.858 (0.743) |
| Wilson B (Å$^2$) | 54.4 | 35.7 | 36.0 | 40.2 | 35.4 | 34.6 | 38.1 | 34.9 | 37.8 | 34.5 | 36.2 | 39.6 | 58.3 |

**Table S2** Structure solution and refinement

ID29 model statistics; values for the outer shell are given in parentheses.

| Data set | ID29, 100×100 | ID29, 75×75 | ID29, 50×50 | ID29, 25×100 | ID29, 25×75 | ID29, 25×50 | ID29, 25×25 | ID29, 15×100 | SwissFEL, 100×100 | SACLA* | SACLA reproc.** |
|---|---|---|---|---|---|---|---|---|---|---|---|
| pdb code | 9GT0 | 9GT1 | 9GT2 | 9GT3 | 9GT4 | 9GT5 | 9GT6 | 9GT7 | 9GT8 | 9GT9 | 9GTA |
| Resolution range (Å) | 40-1.67 (1.71-1.67) | 40-1.67 (1.71-1.67) | 40-1.60 (1.64-1.60) | 40-1.67 (1.71-1.67) | 40-1.67 (1.71-1.67) | 40-1.63 (1.67-1.64) | 40-1.57 (1.61-1.57) | 40-1.67 (1.71-1.67) | 40-1.54 (1.58-1.54) | 40-1.88 (1.93-1.88) | 27-1.86 (1.93-1.86) |
| Compl. (%) | 100 (100) | 100 (100) | 100 (100) | 100 (100) | 100 (100) | 100 (100) | 100 (100) | 100 (100) | 100 (100) | 100 (100) | 100 (100) |
| No. of reflections, working set | 79872 | 79852 | 90829 | 79978 | 79832 | 85859 | 96135 | 80113 | 97505 | 54473 | 59125 |
| No. of reflections, test set | 4325 | 4325 | 4943 | 4329 | 4320 | 4666 | 5215 | 4333 | 5282 | 3931 | 3134 |
| Final $R_{cryst}$ | 0.146 (0.384) | 0.152 (0.406) | 0.136 (0.375) | 0.151 (0.404) | 0.153 (0.418) | 0.140 (0.378) | 0.145 (0.448) | 0.146 (0.399) | 0.168 (0.250) | 0.136 (0.422) | 0.148 (0.548) |
| Final $R_{free}$ | 0.172 (0.422) | 0.182 (0.418) | 0.159 (0.405) | 0.175 (0.404) | 0.179 (0.423) | 0.160 (0.379) | 0.168 (0.470) | 0.170 (0.414) | 0.193 (0.279) | 0.176 (0.473) | 0.173 (0.551) |
| No. of non-H atoms | 6337 | 6340 | 6359 | 6337 | 6331 | 6317 | 6350 | 6330 | 6365 | 6220 | 6194 |
| Protein | 5632 | 5632 | 5632 | 5632 | 5632 | 5632 | 5632 | 5632 | 5562 | 5598 | 5568 |
| Ligand | 86 | 86 | 86 | 86 | 86 | 86 | 86 | 86 | 86 | 86 | 86 |
| Water | 619 | 622 | 641 | 619 | 613 | 599 | 726 | 612 | 717 | 536 | 540 |
| Total | | | | | | | | | | | |
| R.m.s. deviations | | | | | | | | | | | |
| Bonds (Å) | 0.010 | 0.010 | 0.012 | 0.010 | 0.010 | 0.012 | 0.012 | 0.011 | 0.014 | 0.014 | 0.016 |
| Angles (°) | 1.682 | 1.647 | 1.835 | 1.692 | 1.645 | 1.798 | 1.813 | 1.735 | 1.832 | 1.880 | 1.75 |
| Average *B* factors (Å$^2$) | | | | | | | | | | | |
| Protein | 25.0 | 25.9 | 25.4 | 25.2 | 25.7 | 26.0 | 25.9 | 26.4 | 17.0 | 31.6 | 28.9 |
| Ligand | 17.5 | 17.4 | 18.2 | 17.9 | 18.3 | 18.8 | 18.8 | 18.8 | 11.5 | 23.8 | 21.0 |
| Water | 37.6 | 37.2 | 39.3 | 38.2 | 37.6 | 38.1 | 39.3 | 39.1 | 32.0 | 42.5 | 41.4 |

* Re-refinement against data from 6i43

**Re-processing of original stream files from 6i43, refinement against reprocessed data

Cristallina model statistics (June 2023 data); Values for the outer shell are given in parentheses.

| Data set<br>pdb code | Cristallina 25x25 0.3T | Cristallina 50x25 | Cristallina 75x25 | Cristallina 100x25 | Cristallina 50x50 0.3T | Cristallina 75x50 | Cristallina 75x75 0.3T | Cristallina 100x100 |
|---|---|---|---|---|---|---|---|---|
| Resolution range (Å) | 37.0-1.54 (1.58-1.54) | 37.0-1.54 (1.58-1.54) | 37.0-1.54 (1.58-1.54) | 37.0-1.54 (1.58-1.54) | 37.0-1.54 (1.58-1.54) | 37.0-1.54 (1.58-1.54) | 37.0-1.54 (1.58-1.54) | 37.0-1.54 (1.58-1.54) |
| Compl. (%) | 100 (97) | 100 (100) | 100 (100) | 100 (100) | 100 (97) | 100 (100) | 100 (100) | 100 (100) |
| No. of reflections, working set | 92179 | 92428 | 92353 | 92537 | 92177 | 92465 | 92427 | 92223 |
| No. of reflections, test set | 5280 | 5292 | 5286 | 5296 | 5280 | 5295 | 5292 | 5282 |
| Final $R_{cryst}$ | 0.177 (0.252) | 0.152 (0.207) | 0.167 (0.236) | 0.168 (0.239) | 0.153 (0.21) | 0.168 (0.241) | 0.146 (0.199) | 0.168 (0.248) |
| Final $R_{free}$ | 0.206 (0.288) | 0.176 (0.241) | 0.192 (0.277) | 0.194 (0.277) | 0.177 (0.258) | 0.196 (0.28) | 0.171 (0.235) | 0.192 (0.279) |
| No. of non-H atoms | | | | | | | | |
| Protein | 5562 | 5562 | 5562 | 5562 | 5562 | 5562 | 5562 | 5562 |
| Ligand | 86 | 86 | 86 | 86 | 86 | 86 | 86 | 86 |
| Water | 720 | 717 | 720 | 720 | 720 | 720 | 720 | 717 |
| Total | 6368 | 6365 | 6368 | 6368 | 6368 | 6368 | 6368 | 6365 |
| R.m.s. deviations | | | | | | | | |
| Bonds (Å) | 0.011 | 0.013 | 0.011 | 0.011 | 0.012 | 0.011 | 0.012 | 0.011 |
| Angles (°) | 1.826 | 2.015 | 1.886 | 1.867 | 1.927 | 1.872 | 1.982 | 1.852 |
| Average *B* factors ($Å^2$) | | | | | | | | |
| Protein | 17.0 | 17.9 | 17.7 | 17.7 | 17.4 | 17.6 | 17.6 | 17.0 |
| Ligand | 11.5 | 12.3 | 12.2 | 12.2 | 11.7 | 12.0 | 11.9 | 11.4 |
| Water | 32.2 | 33.2 | 32.7 | 32.9 | 32.7 | 32.8 | 32.7 | 32.0 |

Cristallina model statistics (continued) September 2023 data; Values for the outer shell are given in parentheses.

| Data set<br>pdb code | Cristallina 10x10 small ds1 | Cristallina 10x10_small ds2 | Cristallina 20x10_small | Cristallina 15x15 small | Cristallina 20x15 small | Cristallina 10x20 small | Cristallina 20x20 small ds1 | Cristallina 20x20 small ds2 | Cristallina 20x20 small ds3 | Cristallina 25x25 small | Cristallina 50x50 small |
|---|---|---|---|---|---|---|---|---|---|---|---|
| Resolution range (Å) | 38.0-1.57 (1.61-1.57) | 38.0-1.6 (1.64-1.6) | 38.0-1.57 (1.61-1.57) | 38.0-1.54 (1.58-1.54) | 38.0-1.54 (1.58-1.54) | 38.0-1.54 (1.58-1.54) | 38.0-1.71 (1.75-1.71) | 37.0-1.54 (1.58-1.54) | 38.0-1.54 (1.58-1.54) | 38.0-1.54 (1.58-1.54) | 38.0-1.54 (1.58-1.54) |
| Compl. (%) | 100 (100) | 100 (99) | 100 (100) | 100 (100) | 100 (100) | 100 (100) | 100 (99) | 100 (100) | 100 (100) | 100 (100) | 100 (100) |
| No. of reflections, working set | 91081 | 85827 | 90937 | 96354 | 96300 | 96415 | 70261 | 96423 | 96559 | 96444 | 96293 |
| No. of reflections, test set | 5224 | 4924 | 5218 | 5507 | 5504 | 5511 | 4024 | 5512 | 5521 | 5514 | 5502 |
| Final $R_{cryst}$ | 0.174 (0.307) | 0.197 (0.326) | 0.177 (0.32) | 0.143 (0.26) | 0.149 (0.281) | 0.142 (0.242) | 0.219 (0.29) | 0.149 (0.263) | 0.15 (0.248) | 0.14 (0.222) | 0.15 (0.198) |
| Final $R_{free}$ | 0.204 (0.318) | 0.227 (0.329) | 0.206 (0.34) | 0.165 (0.276) | 0.175 (0.285) | 0.165 (0.254) | 0.249 (0.318) | 0.176 (0.274) | 0.176 (0.268) | 0.166 (0.228) | 0.173 (0.218) |
| No. of non-H atoms | | | | | | | | | | | |
| Protein | 5630 | 5650 | 5650 | 5650 | 5650 | 5650 | 5496 | 5618 | 5623 | 5644 | 5644 |
| Ligand | 86 | 86 | 86 | 86 | 86 | 86 | 86 | 86 | 86 | 86 | 86 |
| Water | 680 | 676 | 676 | 676 | 676 | 677 | 249 | 829 | 829 | 707 | 706 |
| Total | 6396 | 6412 | 6412 | 6412 | 6412 | 6413 | 5831 | 6533 | 6538 | 6437 | 6436 |
| R.m.s. deviations | | | | | | | | | | | |
| Bonds (Å) | 0.008 | 0.007 | 0.008 | 0.012 | 0.011 | 0.012 | 0.008 | 0.012 | 0.011 | 0.012 | 0.011 |
| Angles (°) | 1.622 | 1.498 | 1.622 | 1.969 | 1.945 | 2.038 | 1.718 | 1.941 | 1.946 | 2.018 | 1.892 |
| Average *B* factors (Å$^2$) | | | | | | | | | | | |
| Protein | 22.5 | 21.9 | 22.5 | 22.0 | 22.3 | 22.2 | 23.2 | 21.5 | 20.8 | 20.5 | 17.7 |
| Ligand | 15.0 | 14.5 | 15.2 | 14.4 | 15.0 | 14.8 | 14.8 | 14.3 | 13.8 | 13.6 | 11.6 |
| Water | 37.8 | 36.6 | 37.4 | 37.4 | 37.6 | 37.5 | 29.5 | 42.0 | 41.0 | 36.6 | 34.3 |

Cristallina model statistics (continued) September 2023 data; Values for the outer shell are given in parentheses.

| Data set | Cristallina 5x5 big | Cristallina 10x10 big ds1 | Cristallina 10x10 big ds2 | Cristallina 10x10 big ds3 | Cristallina 20x10 big | Cristallina 15x15 big ds1 | Cristallina 15x15 big ds2 | Cristallina 20x15 big | Cristallina 10x20 big | Cristallina 20x20 big ds1 | Cristallina 20x20 big ds2 | Cristallina 20x20 big ds3 | data-13+DtpAa_1a_25x25_big_refmac100.pdb |
|---|---|---|---|---|---|---|---|---|---|---|---|---|---|
| Resolution range (Å) | 38.0-2.47 (2.53-2.47) | 38.0-1.75 (1.8-1.75) | 38.0-1.8 (1.85-1.8) | 38.0-2.08 (2.13-2.08) | 38.0-1.75 (1.8-1.75) | 38.0-1.75 (1.8-1.75) | 38.0-2.08 (2.13-2.08) | 38.0-1.86 (1.91-1.86) | 38.0-2.0 (2.05-2.0) | 38.0-1.8 (1.85-1.8) | 38.0-2.0 (2.05-2.0) | 38.0-2.18 (2.24-2.18) | 38.0-2.69 (2.76-2.69) |
| Compl. (%) | 100 (100) | 100 (100) | 100 (100) | 100 (100) | 100 (100) | 100 (100) | 100 (100) | 100 (100) | 100 (100) | 100 (100) | 100 (100) | 100 (100) | 100 (99) |
| No. of reflections, working set | 24265 | 67678 | 62152 | 40323 | 67673 | 67672 | 40331 | 56275 | 45234 | 62084 | 45240 | 35059 | 18787 |
| No. of reflections, test set | 1420 | 3902 | 3615 | 2400 | 3900 | 3901 | 2398 | 3306 | 2724 | 3612 | 2726 | 2080 | 1077 |
| Final $R_{cryst}$ | 0.163 (0.298) | 0.178 (0.345) | 0.185 (0.378) | 0.173 (0.37) | 0.181 (0.376) | 0.189 (0.364) | 0.174 (0.331) | 0.171 (0.337) | 0.176 (0.366) | 0.183 (0.362) | 0.179 (0.35) | 0.191 {0.328) | 0.183 {0.346) |
| Final $R_{free}$ | 0.233 (0.31) | 0.211 (0.369) | 0.218 (0.373) | 0.227 (0.397) | 0.214 (0.41) | 0.221 (0.388) | 0.223 (0.349) | 0.217 (0.341) | 0.222 (0.349) | 0.214 (0.362) | 0.229 (0.375) | 0.251 {0.349) | 0.271 {0.444) |
| No. of non-H atoms | | | | | | | | | | | | | |
| Protein | 5496 | 5501 | 5496 | 5496 | 5496 | 5496 | 5496 | 5594 | 5499 | 5496 | 5496 | 5496 | 5496 |
| Ligand | 86 | 86 | 86 | 86 | 86 | 86 | 86 | 86 | 86 | 86 | 86 | 86 | 86 |
| Water | 243 | 306 | 248 | 244 | 359 | 244 | 244 | 567 | 248 | 298 | 244 | 245 | 245 |
| Total | 5825 | 5893 | 5830 | 5826 | 5941 | 5826 | 5826 | 6247 | 5833 | 5880 | 5826 | 5827 | 5827 |
| R.m.s. deviations | | | | | | | | | | | | | |
| Bonds (Å) | 0.007 | 0.009 | 0.007 | 0.008 | 0.008 | 0.008 | 0.008 | 0.01 | 0.009 | 0.007 | 0.009 | 0.007 | 0.007 |
| Angles (°) | 1.713 | 1.644 | 1.58 | 1.701 | 1.586 | 1.582 | 1.796 | 1.825 | 1.809 | 1.525 | 1.786 | 1.56 | 1.82 |
| Average $B$ factors ($Å^2$) | | | | | | | | | | | | | |
| Protein | 51.7 | 42.7 | 43.1 | 47.6 | 42.4 | 40.7 | 43.2 | 42.7 | 44.5 | 40.9 | 42.0 | 42.2 | 42.9 |
| Ligand | 35.1 | 30.4 | 30.0 | 33.2 | 30.3 | 28.7 | 29.9 | 29.6 | 31.1 | 29.0 | 29.0 | 27.3 | 24.0 |
| Water | 48.4 | 48.3 | 44.7 | 47.5 | 50.7 | 42.8 | 43.8 | 55.4 | 44.8 | 45.4 | 42.3 | 41.3 | 34.7 |

**Table S3** a) Interatomic distances, given in Å, with their standard deviations, as a function of x and y step sizes in µm for the A and B molecules: ID29 SSX data

| Δx Fast | Δy Slow | Fe-W (A) | | Fe-His (A) | | FeOOP (A) | | Fe-W (B) | | Fe-His (B) | | FeOOP (B) | | W-W (B) | | Data set |
|---|---|---|---|---|---|---|---|---|---|---|---|---|---|---|---|---|
| n.a. | n.a. | 2.406 | n.a. | 2.155 | n.a. | -0.154 | n.a. | 2.381 | n.a. | 2.107 | n.a. | -0.205 | n.a. | 1.536 | n.a. | SACLA rerefined |
| n.a. | n.a. | 2.392 | ±0.031 | 2.151 | ±0.034 | -0.146 | ±0.008 | 2.412 | ±0.056 | 2.102 | ±0.041 | -0.221 | ±0.008 | 1.580 | ±0.051 | SACLA reprocessed |
| 15 | 100 | 2.473 | ±0.026 | 2.201 | ±0.028 | -0.158 | ±0.008 | 2.423 | ±0.065 | 2.165 | ±0.024 | -0.233 | ±0.006 | 1.683 | ±0.057 | ID29 15×100 |
| 25 | 25 | 2.447 | ±0.020 | 2.170 | ±0.019 | -0.163 | ±0.005 | 2.395 | ±0.102 | 2.132 | ±0.019 | -0.249 | ±0.005 | 1.604 | ±0.061 | ID29 25×25 |
| 25 | 50 | 2.423 | ±0.023 | 2.169 | ±0.061 | -0.157 | ±0.006 | 2.398 | ±0.054 | 2.147 | ±0.021 | -0.256 | ±0.006 | 1.686 | ±0.047 | ID29 25×50 |
| 25 | 75 | 2.446 | ±0.029 | 2.209 | ±0.030 | -0.157 | ±0.008 | 2.443 | ±0.086 | 2.128 | ±0.023 | -0.236 | ±0.006 | 1.818 | ±0.087 | ID29 25×75 |
| 25 | 100 | 2.449 | ±0.032 | 2.186 | ±0.030 | -0.154 | ±0.008 | 2.503 | ±0.123 | 2.149 | ±0.024 | -0.249 | ±0.007 | 1.745 | ±0.096 | ID29 25×100 |
| 50 | 50 | 2.395 | ±0.021 | 2.152 | ±0.020 | -0.156 | ±0.006 | 2.380 | ±0.052 | 2.122 | ±0.021 | -0.261 | ±0.006 | 1.559 | ±0.049 | ID29 50×50 |
| 75 | 75 | 2.464 | ±0.030 | 2.211 | ±0.029 | -0.160 | ±0.009 | 2.410 | ±0.099 | 2.141 | ±0.030 | -0.243 | ±0.007 | 1.723 | ±0.081 | ID29 75×75 |
| 100 | 100 | 2.450 | ±0.027 | 2.188 | ±0.042 | -0.157 | ±0.005 | 2.4570 | ±0.071 | 2.132 | ±0.022 | -0.247 | ±0.008 | 1.506 | ±0.060 | ID29 100×100 |

**Table S3** b) Interatomic distances, given in Å, with their standard deviations, as a function of x and y step sizes in µm for the A and B molecules: Cristallina-MX SFX data, June 2023 and September 2023, small unit cell

| Δy Fast | Δx Slow | Fe-W (A) | | Fe-His (A) | | FeOOP (A) | | Fe-W (B) | | Fe-His (B) | | FeOOP (B) | | W-W (B) | | Data set |
|---|---|---|---|---|---|---|---|---|---|---|---|---|---|---|---|---|
| 25 | 25 | 2.456 | ±0.016 | 2.133 | ±0.031 | -0.149 | ±0.007 | 2.274 | ±0.055 | 2.075 | ±0.031 | -0.203 | ±0.008 | 1.675 | ±0.065 | Cristallina 25x25 0.3T |
| 25 | 50 | 2.423 | ±0.020 | 2.128 | ±0.027 | -0.148 | ±0.007 | 2.239 | ±0.065 | 2.072 | ±0.035 | -0.204 | ±0.008 | 1.726 | ±0.070 | Cristallina 50x25 |
| 25 | 75 | 2.423 | ±0.025 | 2.129 | ±0.039 | -0.144 | ±0.008 | 2.210 | ±0.057 | 2.070 | ±0.039 | -0.202 | ±0.008 | 1.760 | ±0.062 | Cristallina 75x25 |
| 25 | 100 | 2.444 | ±0.021 | 2.142 | ±0.032 | -0.145 | ±0.010 | 2.246 | ±0.052 | 2.067 | ±0.034 | -0.212 | ±0.009 | 1.710 | ±0.081 | Cristallina 100x25 |
| 50 | 50 | 2.445 | ±0.021 | 2.128 | ±0.066 | -0.148 | ±0.009 | 2.245 | ±0.041 | 2.088 | ±0.029 | -0.206 | ±0.009 | 1.720 | ±0.066 | Cristallina 50x50 0.3T |
| 50 | 75 | 2.452 | ±0.023 | 2.133 | ±0.038 | -0.149 | ±0.007 | 2.284 | ±0.049 | 2.084 | ±0.041 | -0.209 | ±0.007 | 1.686 | ±0.058 | Cristallina 75x50 |
| 75 | 75 | 2.438 | ±0.021 | 2.139 | ±0.032 | -0.140 | ±0.010 | 2.245 | ±0.048 | 2.080 | ±0.029 | -0.199 | ±0.009 | 1.728 | ±0.076 | Cristallina 75x75 0.3T |
| 100 | 100 | 2.454 | ±0.024 | 2.152 | ±0.044 | -0.143 | ±0.011 | 2.296 | ±0.061 | 2.090 | ±0.038 | -0.205 | ±0.010 | 1.663 | ±0.085 | Cristallina 100x100 |
| Sept 2023 | | | | | | | | | | | | | | | | |
| 10 | 10 | 2.30 | ±0.040 | 2.139 | ±0.036 | -0.119 | ±0.011 | 1.945 | ±0.0493 | 2.103 | ±0.044 | -0.121 | ±0.010 | 2.039 | ±0.199 | Cristallina 10x10 small ds1 |
| 10 | 10 | 2.41 | ±0.055 | 2.151 | ±0.043 | -0.125 | ±0.011 | 1.978 | ±0.122 | 2.112 | ±0.040 | -0.112 | ±0.011 | 2.009 | ±0.189 | Cristallina 10x10 small ds2 |
| 10 | 20 | 2.294 | ±0.040 | 2.153 | ±0.035 | -0.115 | ±0.008 | 1.901 | ±0.038 | 2.136 | ±0.039 | -0.121 | ±0.009 | 2.026 | ±0.149 | Cristallina 20x10 small |
| 15 | 15 | 2.247 | ±0.020 | 2.115 | ±0.021 | -0.119 | ±0.005 | 1.864 | ±0.024 | 2.141 | ±0.023 | -0.098 | ±0.006 | 2.040 | ±0.132 | Cristallina 15x15 small |
| 15 | 20 | 2.263 | ±0.024 | 2.117 | ±0.025 | -0.115 | ±0.006 | 1.900 | ±0.029 | 2.120 | ±0.023 | -0.117 | ±0.006 | 1.981 | ±0.100 | Cristallina 20x15 small |
| 20 | 10 | 2.282 | ±0.021 | 2.102 | ±0.017 | -0.131 | ±0.005 | 1.950 | ±0.024 | 2.124 | ±0.018 | -0.141 | ±0.006 | 1.975 | 0.074 | Cristallina 10x20 small |
| 20 | 20 | 2.440 | ±0.052 | 2.082 | ±0.041 | -0.123 | ±0.012 | 1.877 | ±0.073 | 2.164 | ±0.053 | -0.070 | ±0.013 | 2.739 | ±0.105 | Cristallina 20x20 small ds1 |
| 20 | 20 | 2.277 | ±0.024 | 2.110 | ±0.021 | -0.130 | ±0.005 | 1.947 | ±0.026 | 2.120 | ±0.014 | -0.143 | ±0.005 | 2.007 | ±0.098 | Cristallina 20x20 small ds2 |
| 20 | 20 | 2.302 | ±0.022 | 2.149 | ±0.017 | -0.130 | ±0.006 | 2.030 | ±0.027 | 2.113 | ±0.017 | -0.177 | ±0.006 | 1.851 | ±0.154 | Cristallina 20x20 small ds3 |
| 25 | 25 | 2.326 | ±0.021 | 2.141 | ±0.018 | -0.136 | ±0.005 | 2.067 | ±0.034 | 2.113 | ±0.024 | -0.195 | ±0.006 | 1.786 | ±0.085 | Cristallina 25x25 small ds1 |

| | | | | | | | | | | | | | | | | |
|---|---|---|---|---|---|---|---|---|---|---|---|---|---|---|---|---|
| 50 | 50 | 2.391 | ±0.019 | 2.151 | ±0.018 | -0.149 | ±0.006 | 2.313 | ±0.046 | 2.123 | ±0.017 | -0.239 | ±0.005 | 1.569 | ±0.057 | Cristallina 50x50 small ds1 |

**Table S3** **c**) Interatomic distances, given in Å, with their standard deviations, as a function of x and y step sizes in µm for the A and B molecules: Cristallina-MX SFX data, September 2023, large unit cell

| Δy Fast | Δx Slow | Fe-W (A) | | Fe-His (A) | | FeOOP (A) | | Fe-W (B) | | Fe-His (B) | | FeOOP (B) | | W-W (B) | | Data set |
|---|---|---|---|---|---|---|---|---|---|---|---|---|---|---|---|---|
| 5 | 5 | n.a. | n.a. | 2.191 | ±0.073 | 0.0380 | ±0.023 | n.a | n.a. | 2.237 | ±0.090 | 0.040 | ±0.022 | n.a. | n.a | Cristallina 5x5 big |
| 10 | 10 | 1.823 | ±0.055 | 2.164 | ±0.029 | -0.0390 | ±0.010 | 1.911 | ±0.041 | 2.264 | ±0.041 | -0.012 | ±0.009 | 2.663 | ±0.053 | Cristallina 10x10 big ds1 |
| 10 | 10 | 1.813 | ±0.067 | 2.151 | ±0.062 | -0.0400 | ±0.013 | 1.759 | ±0.050 | 2.294 | ±0.058 | -0.033 | ±0.012 | 2.685 | ±0.062 | Cristallina 10x10 big ds2 |
| 10 | 10 | 1.762 | ±0.059 | 2.169 | ±0.052 | -0.0640 | ±0.017 | 1.589 | ±0.097 | 2.291 | ±0.066 | -0.029 | ±0.017 | 2.862 | ±0.088 | Cristallina 10x10 big ds3 |
| 10 | 20 | 1.829 | ±0.039 | 2.116 | ±0.033 | -0.0570 | ±0.010 | 1.876 | ±0.046 | 2.297 | ±0.051 | -0.016 | ±0.013 | 2.640 | ±0.072 | Cristallina 20x10 big |
| 15 | 15 | 1.828 | ±0.042 | 2.147 | ±0.030 | -0.0370 | ±0.010 | 1.827 | ±0.047 | 2.282 | ±0.046 | -0.014 | ±0.013 | 2.775 | ±0.066 | Cristallina 15x15 big ds1 |
| 15 | 15 | 1.864 | ±0.078 | 2.211 | ±0.041 | -0.0450 | ±0.016 | 1.750 | ±0.091 | 2.281 | ±0.062 | 0.000 | ±0.017 | 2.879 | ±0.095 | Cristallina 15x15 big ds2 |
| 15 | 20 | 1.964 | ±0.076 | 2.072 | ±0.032 | -0.0760 | ±0.012 | 1.827 | ±0.050 | 2.323 | ±0.052 | -0.030 | ±0.012 | 2.705 | ±0.070 | Cristallina 20x15 big |
| 20 | 10 | 2.045 | ±0.067 | 2.161 | ±0.043 | -0.0890 | ±0.013 | 1.686 | ±0.065 | 2.246 | ±0.079 | -0.045 | ±0.016 | 2.830 | ±0.088 | Cristallina 10x20 big |
| 20 | 20 | 1.883 | ±0.062 | 2.145 | ±0.035 | -0.0720 | ±0.011 | 1.807 | ±0.042 | 2.281 | ±0.055 | -0.017 | ±0.013 | 2.779 | ±0.074 | Cristallina 20x20 big ds1 |
| 20 | 20 | 1.978 | ±0.057 | 2.183 | ±0.046 | -0.0850 | ±0.015 | 1.780 | ±0.066 | 2.254 | ±0.072 | -0.031 | ±0.017 | 2.763 | ±0.090 | Cristallina 20x20 big ds2 |
| 20 | 20 | 1.906 | ±0.085 | 2.236 | ±0.053 | -0.1130 | ±0.017 | 3.792 | ±0.194 | 2.297 | ±0.067 | 0.036 | ±0.022 | n.a | n.a | Cristallina 20x20 big ds3 |

**Table S3** d) Interatomic distances, given in Å, with their standard deviations, as a function of x and y step sizes in µm for the A and B molecules

| Δx | Δy | Fe-W (A) | | Fe-His (A) | | FeOOP (A) | | Fe-W (B) | | Fe-His (B) | | FeOOP (B) | | W-W (B) | | Data set |
|---|---|---|---|---|---|---|---|---|---|---|---|---|---|---|---|---|
| n.a. | n.a. | 2.406 | n.a. | 2.155 | n.a. | -0.154 | n.a. | 2.381 | n.a. | 2.107 | n.a. | -0.205 | n.a. | 1.536 | n.a. | SACLA rerefined |
| n.a. | n.a. | 2.392 | ±0.031 | 2.151 | ±0.034 | -0.146 | ±0.008 | 2.412 | ±0.056 | 2.102 | ±0.041 | -0.221 | ±0.008 | 1.58 | ±0.051 | SACLA reprocessed |
| 100 | 100 | 2.455 | ±0.019 | 2.151 | ±0.032 | -0.146 | ±0.009 | 2.31 | ±0.083 | 2.089 | ±0.033 | -0.209 | ±0.008 | 1.594 | ±0.08 | SwissFEL 100×100 |
| 15 | 100 | 2.464 | ±0.028 | 2.189 | ±0.022 | -0.161 | ±0.007 | 2.4 | ±0.047 | 2.163 | ±0.024 | -0.232 | ±0.007 | 1.503 | ±0.049 | ID29 15×100 |
| 25 | 25 | 2.453 | ±0.018 | 2.158 | ±0.021 | -0.167 | ±0.005 | 2.392 | ±0.056 | 2.135 | ±0.019 | -0.253 | ±0.004 | 1.476 | ±0.043 | ID29 25×25 |
| 25 | 50 | 2.419 | ±0.02 | 2.163 | ±0.022 | -0.159 | ±0.006 | 2.441 | ±0.055 | 2.152 | ±0.019 | -0.259 | ±0.006 | 1.524 | ±0.045 | ID29 25×50 |
| 25 | 75 | 2.447 | ±0.029 | 2.202 | ±0.028 | -0.159 | ±0.007 | 2.425 | ±0.064 | 2.128 | ±0.026 | -0.236 | ±0.007 | 1.592 | ±0.073 | ID29 25×75 |
| 25 | 100 | 2.453 | ±0.029 | 2.18 | ±0.029 | -0.157 | ±0.008 | 2.468 | ±0.069 | 2.142 | ±0.025 | -0.247 | ±0.008 | 1.549 | ±0.076 | ID29 25×100 |
| 50 | 50 | 2.395 | ±0.019 | 2.152 | ±0.018 | -0.159 | ±0.005 | 2.38 | ±0.057 | 2.122 | ±0.02 | -0.264 | ±0.006 | 1.559 | ±0.048 | ID29 50×50 |
| 75 | 75 | 2.462 | ±0.026 | 2.195 | ±0.027 | -0.164 | ±0.008 | 2.425 | ±0.079 | 2.141 | ±0.028 | -0.244 | ±0.008 | 1.537 | ±0.071 | ID29 75×75 |
| 100 | 100 | 2.450 | ±0.028 | 2.188 | ±0.028 | -0.159 | ±0.008 | 2.457 | ±0.068 | 2.132 | ±0.024 | -0.25 | ±0.007 | 1.506 | ±0.068 | ID29 100×100 |

a)

b)

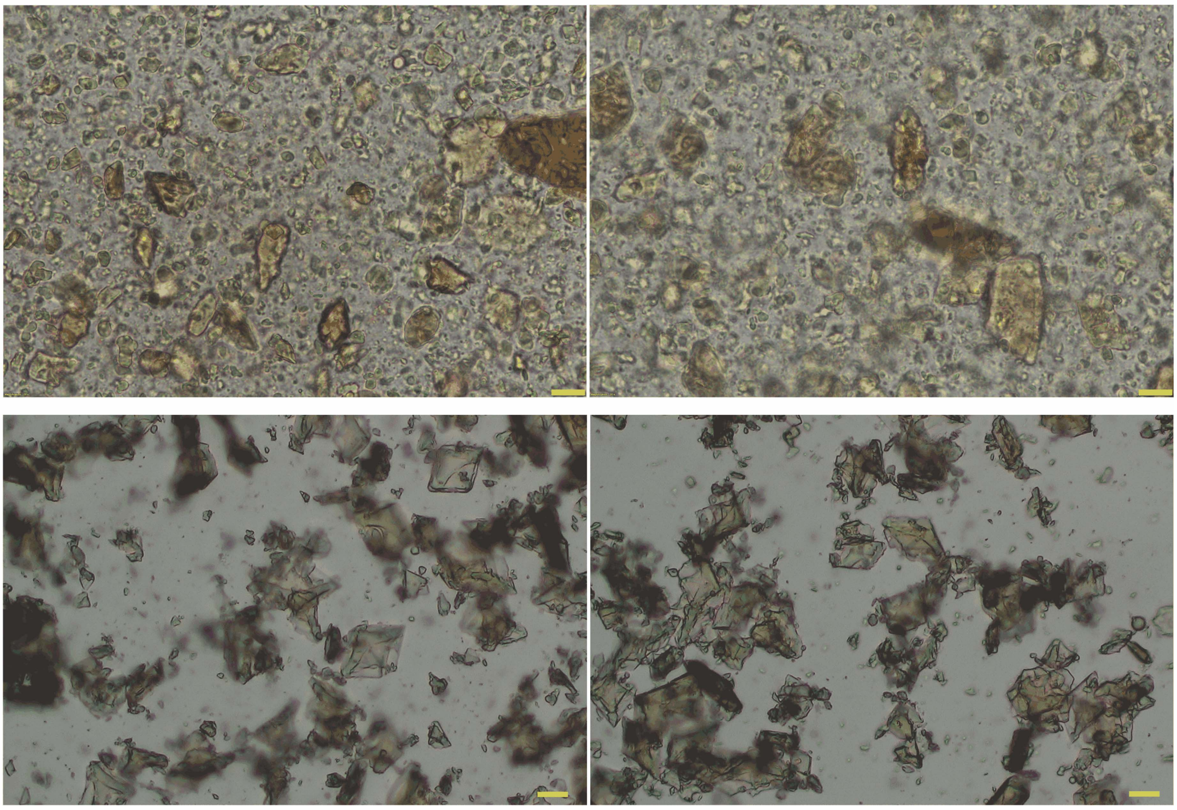

**Supplemental Fig. S1** DtpAa crystals used for serial data collection. a) Batch1 was used for data collection in June 2023 at Cristallina-MX. b) Batch2 was used for data collection in September 2023 at Cristallina-MX and in February 2024 at ID29. The yellow scale bars shown at the lower right corners are 20 µm. The crystals shown in a) are embedded in hydroxyethyl-cellulose, the crystals shown in b) are in mother liquor.

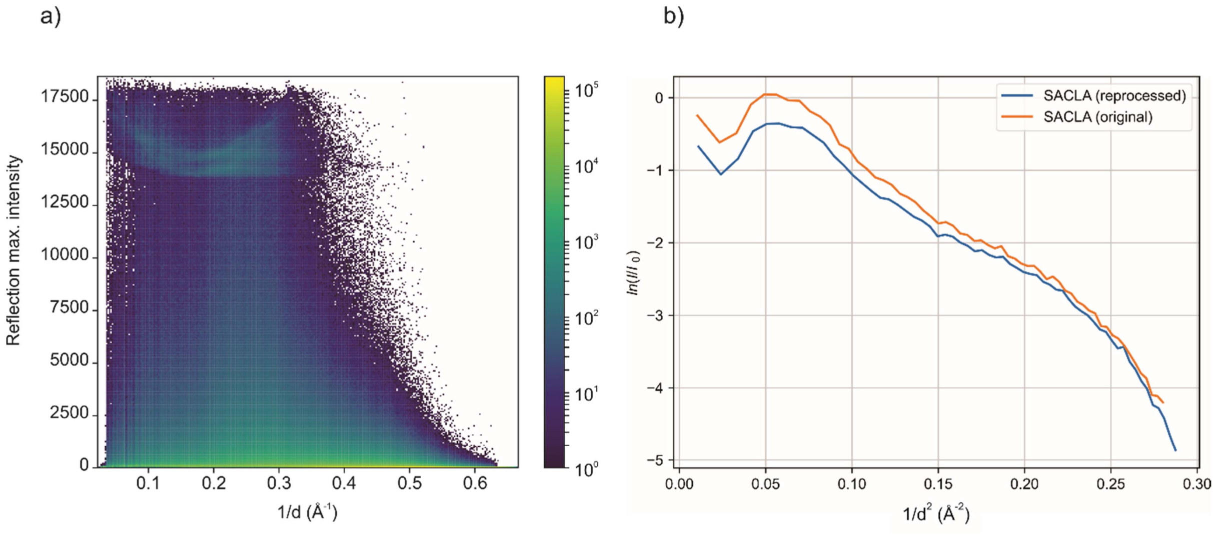


**Supplemental Fig. S2.** Analysis of the SACLA data deposited by (Ebrahim *et al.*, 2019). a) The data suffer from serious pixel saturation as shown by the "peakogram" calculated by CrystFEL. The peakogram shows a histogram of Bragg reflection pixel values as a function of resolution. The accumulation of large numbers of pixels with values above 13,000 ADC counts (green bands in the top left corner) is indicative of considerable saturation. b) Wilson plot. The strong curvature at high resolution is unusual.

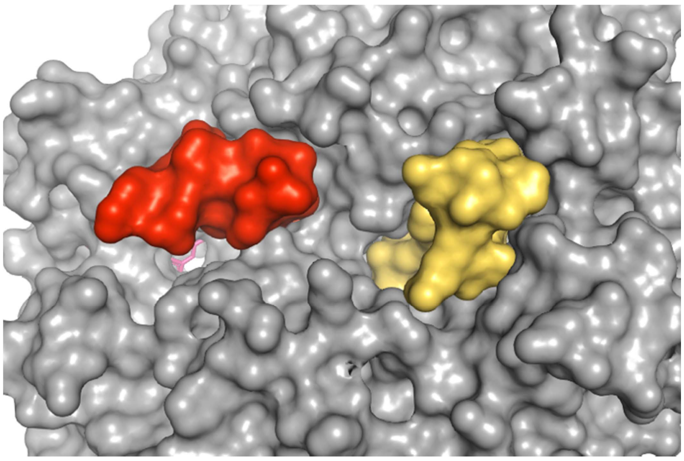

**Supplemental Fig. S3.** Surface view of the DtpAa dimer in the asymmetric unit. The orientation of the amino acid stretch (residues 217-230) in the A-molecule (yellow) and the B-molecule (red) differs. In the latter case a tunnel exists that leads towards the heme in the B molecule (pink sticks).

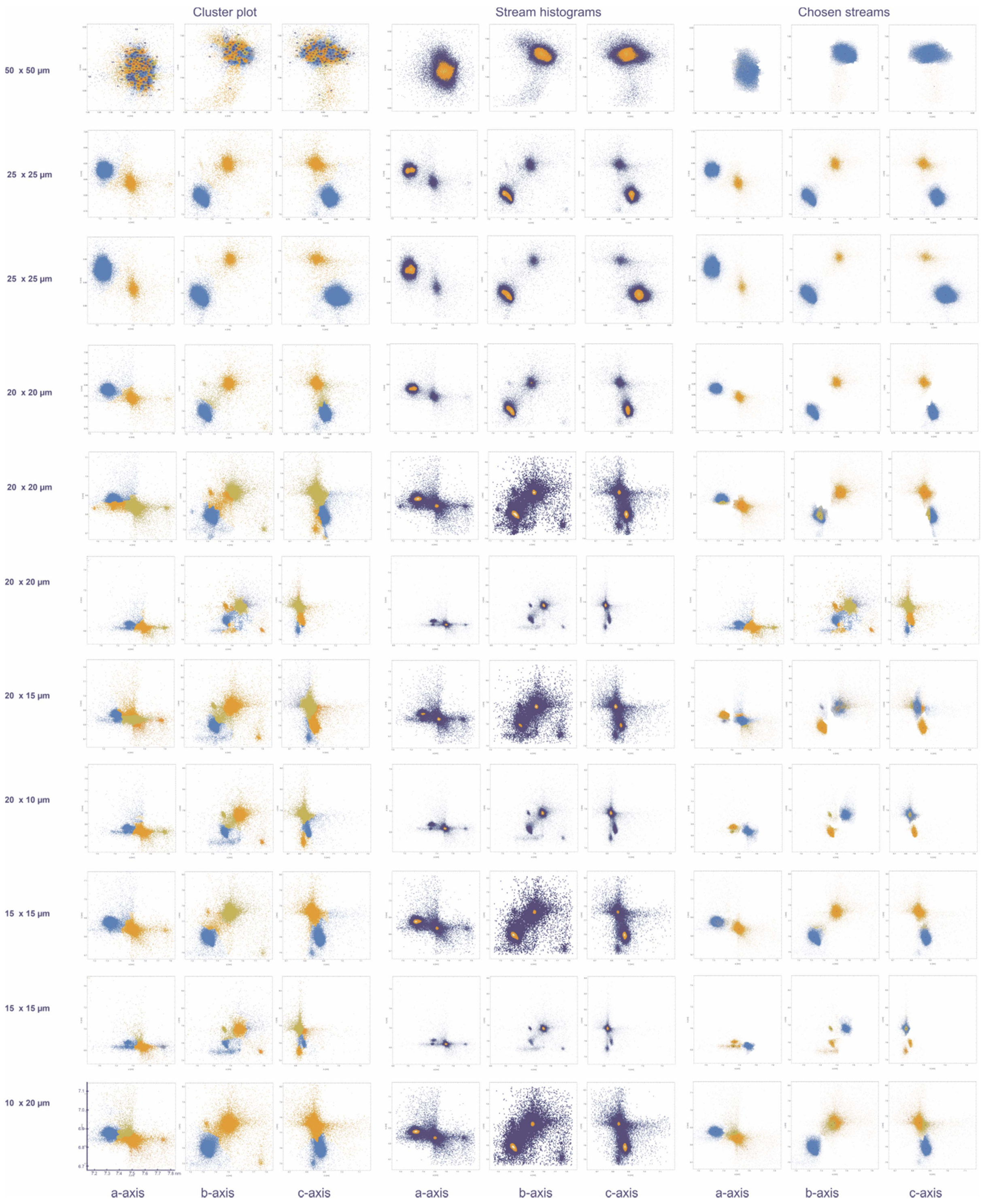
Cluster plot
Stream histograms
Chosen streams
50 x 50 µm
25 x 25 µm
25 x 25 µm
20 x 20 µm
20 x 20 µm
20 x 20 µm
20 x 15 µm
20 x 10 µm
15 x 15 µm
15 x 15 µm
10 x 20 µm
a-axis
b-axis
c-axis
a-axis
b-axis
c-axis
a-axis
b-axis
c-axis

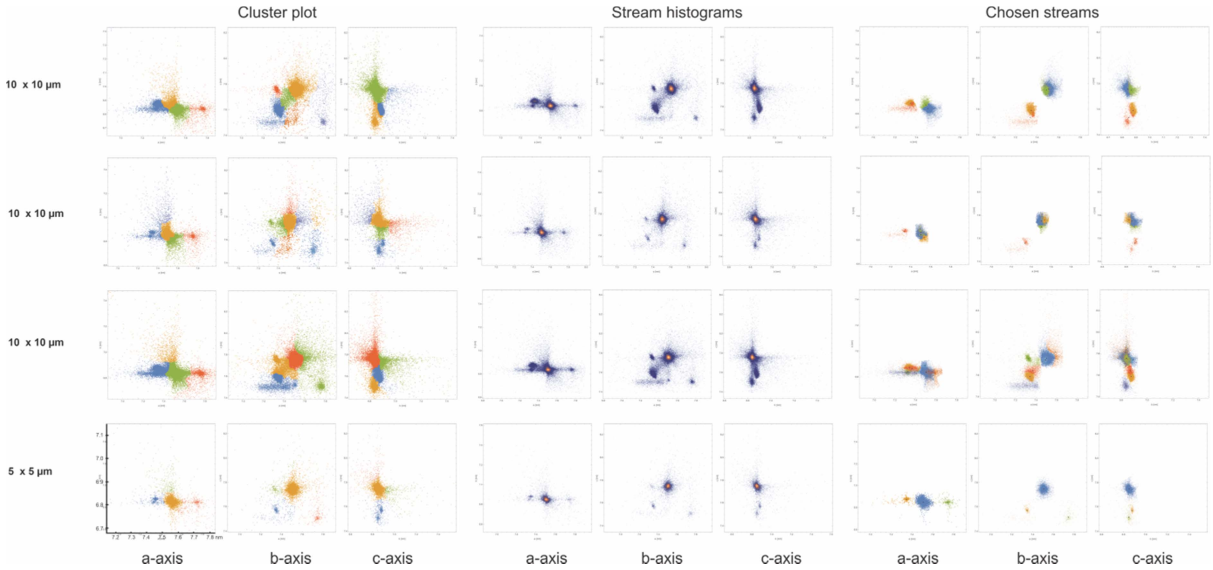


**Supplemental Fig. S4** Unit cell distributions of the various DtpAa SFX data sets collected in September 2023 at Cristallina-MX as a function of the X-ray step size. The unit cell *b* parameter is plotted over *a*, *c* over *b* and *a* over *c*. On the left is the data clustering result, in the center is the density plot of the unit cell parameters and on the right are the clusters that were identified from the data and used in downstream data processing. The Neighbourhood Contraction algorithm was used to obtain the clusters of unit cell parameters. It is designed to partition data into clusters based on density, allowing for arbitrary cluster shapes and sizes. The algorithm iteratively shifts data points towards regions of higher density. At each step a data point is updated to the mean of its neighboring points within a ball of radius R. The data points eventually collapse into fixed points, which represent the centers of the clusters. The algorithm repeats updates until all points stop moving (with respect to a tolerance value). The neighborhood radius R needs to be tuned to obtain optimal cluster separation. The clustering of this data reveals that two main populations of the unit cell appear, the small cell (*a*=73.4 Å, *b*=68.8 Å, *c*=75.9 Å, β=105.7°) and the big cell (*a*=75.0 Å, *b*=68.4 Å, *c*=77.6 Å, β=107.6°). With increasing X-ray step size from 5 × 5 µm to 50 × 50 µm we observe that the composition of the unit cell ensemble changes from mainly big unit cell to mainly small unit cell while a mixture can be observed in between. Clustering the 50 × 50 µm data was difficult due to the low density of the tail next to the main population. A small clustering radius of 0.03 Å was applied to overcluster the data and merge the many clusters from the main population (head) and separate from the low-density cluster (tail). Towards 5 × 5 µm X-ray step size smaller cluster appear in the data which were successfully separated by a clustering radius of 0.13 Å. It is conceivable that the additional cluster with the larger unit cells appear as more energy is dumped from previous X-ray shots into unit area at smaller X-ray steps and the crystals positioned next to the exposed area warm up and/or are exposed to hydrogen gas, resulting in crystal dehydration, manifested by different unit cells. This can be observed clearly for the *b*-axis of the 10 × 10 µm data as a stripe feature (blue cluster) in the unit cell parameter when the unit cell is randomly expanding in the *b*-axis.

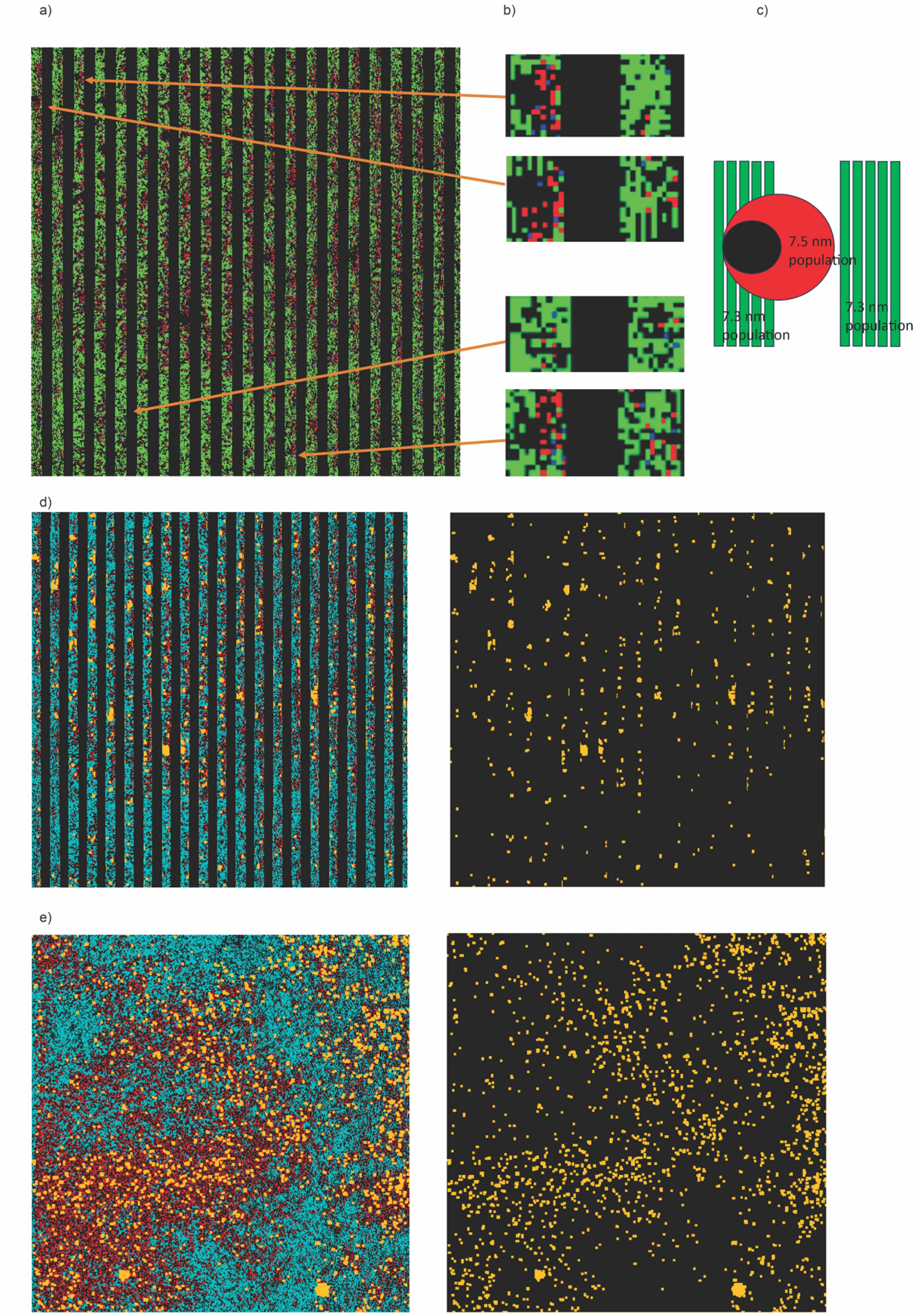


**Supplemental Fig. S5.** The diffraction information recorded during a SOS raster scan can be summarized as a function of the spatial (and thereby also temporal) coordinates of each X-ray

exposure recorded during the scan. To do so we employ two dimensional "dot plots," in which a dot is placed at the (x,y) coordinate of the corresponding X-ray exposure and color-coded according to diffraction information extracted from that exposure. Dot plots are shown for two different runs collected with 20 × ~20 µm X-ray spacing at Cristallina-MX in June 2023. One chip was collected with the X-ray shutter periodically closed (a-d), the other one with the X-ray shutter open during the entire data collection (e). During data collection the SOS chip was moved vertically down then up from left to right. The black vertical stripes indicate times when the chip moved but the X-ray shutter was closed, resulting in no indexed patterns. Patterns with the small / large unit cells are indicated by various colors in the different plots: (a) green (small) / red (large) unit cell, d,e) cyan (small) / red (large) unit cell). It is apparent that the large unit cell (red) populations are seen preferentially just before the left side of a black stripe is reached, namely after a significant number of adjacent X-ray scan lines had been completed. In contrast, predominantly small unit cell crystals are detected consistently just off to the right side of a black segment and so immediately after the previously closed X-ray shutter was reopened. b) When zooming in on some areas of the reconstruction shown in a) it appears as if the red dots representing large unit cell crystals cluster predominantly around empty zones that are likely gas bubbles as sketched in c). d) shows the same data as a) with empty areas/bubbles indicated in yellow. Again, it is striking that the red dots representing large unit cell crystals cluster predominantly around empty zones. e) This data was collected with the X-ray shutter open continuously. There are large clusters of red dots, corresponding to the large unit cell. Again, bubbles (yellow) and large unit cell crystals (red) colocalize. The average size of the bubbles is 80 µm. This is large enough to be easily detectable by optical microscopy. The fact that these bubbles were not observed after loading the chips and before data collection suggests they are a product of data collection, possibly of hydrogen gas generated upon radiolysis of water (Meents *et al.*, 2010). It is possible that hydrogen gas plays a major role in crystal dehydration, a process dubbed "radiodessication" by James Holton (https://bl831.als.lbl.gov/~jamesh/powerpoint/movies/radiodessication.avi). Very large bubbles, as seen in Fig. 11, apparently form slowly after X-ray exposure, likely by consolidation of smaller bubbles. Post-exposure bubble formation is more likely for SOS chips that were scanned with a tight raster. Clearly, these dot plots provide extensive and valuable insight that compensates significantly for the absence in SOS chips of the isolating wells of a structured solid chip. That having been said, however, a similar dot plot analysis might also prove equally informative for solid structured chips.

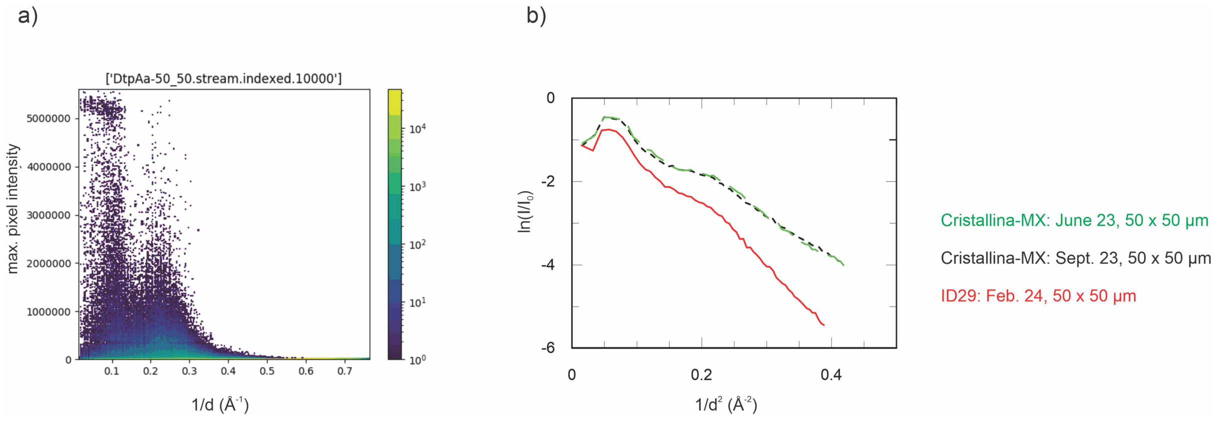


**Supplemental Fig. S6.** a)"Peakogram" calculated by CrystFEL of the ID29 50 × 50 µm data (February 2024). The peakogram shows a histogram of Bragg reflection pixel values as a function of resolution. The intensity distribution is normal. Thus, it is not the reason for the difference in Wilson plots shown in b), displaying a faster decreasing slope of the high-resolution ID29 data compared to the Cristallina-MX data.

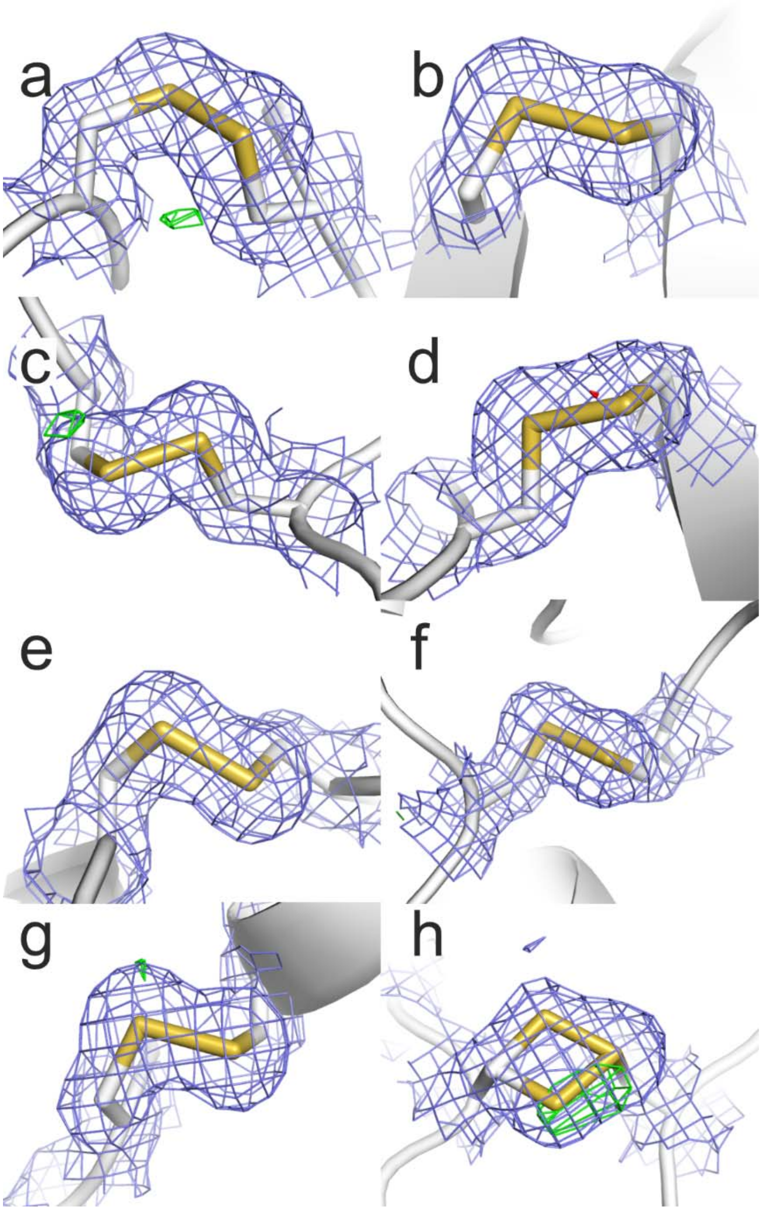


**Supplemental Figure S7.** Electron density maps for thaumatin data collected using sheet-on-sheet chips at ID29 described in (Doak *et al.*, 2024). The panels show the 2*m*Fo-*D*Fc density (blue, 1σ) and the *m*Fo-*D*Fc density (green/red, +3σ /-3σ) around the disulfide bonds in thaumatin. No evidence of S-S bond cleavage is apparent in either map. The bonds shown are a) Cys9-Cys204, b) Cys56-Cys66, c) Cys71-Cys77, d) Cys121-Cys193, e) Cys126-Cys177, f) Cys134-Cys145, g) Cys149-Cys158 and h) Cys149-Cys158, which was modelled in two conformations.

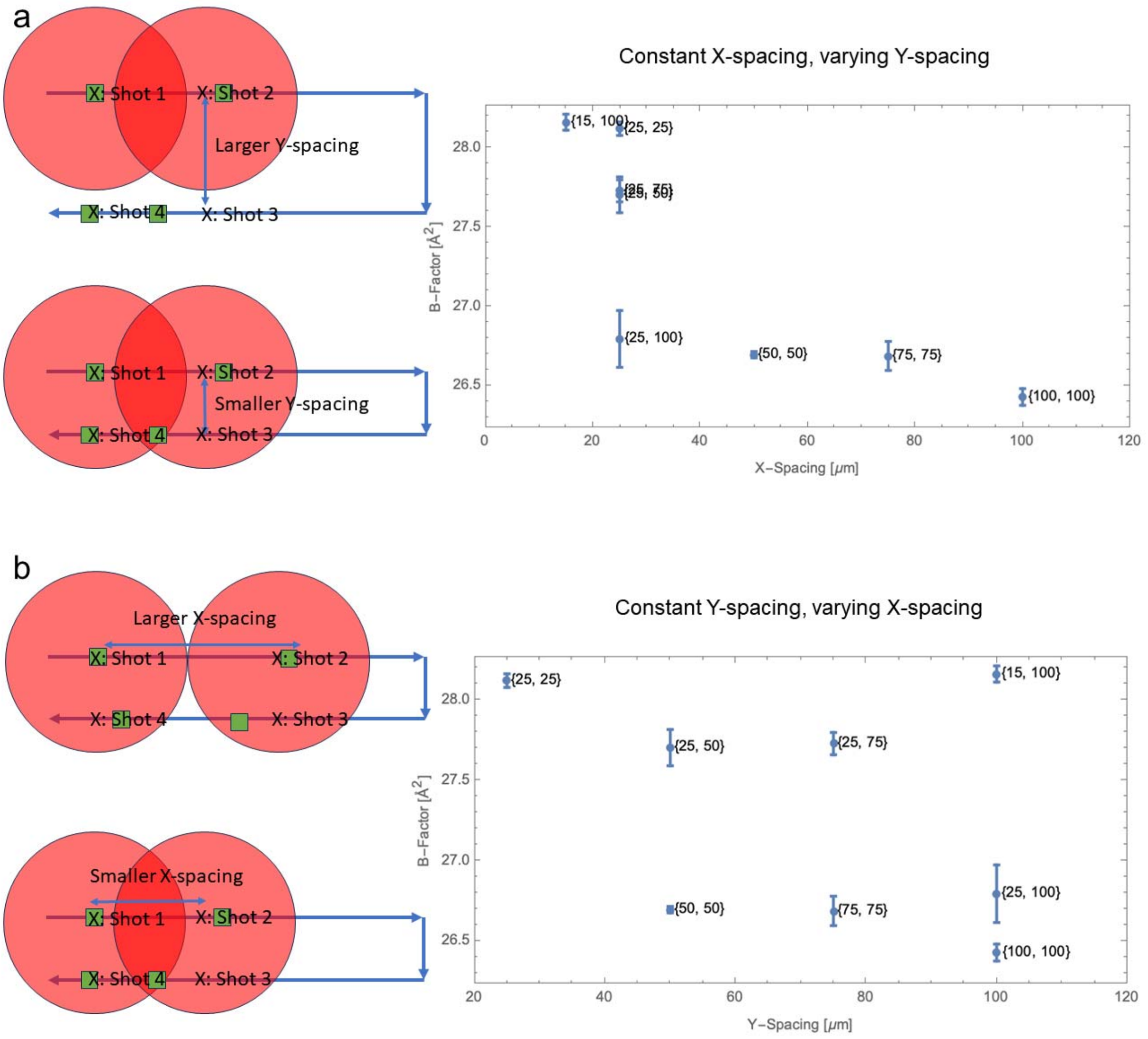


**Supplemental Fig. S8.** Plot of the Wilson B-factors of the ID29 data (February 2024) as a function of the step size between data collection lines (y-spacing, Δy, (a)) and within lines (x-spacing, Δx (b)). Data collected with shorter spacings between X-ray exposures show a slightly increased Wilson B-factor.

## S1. Supplementary Note: Refinement of the distal ligand in the B-molecule.

When assuming and refining a fully occupied heme ligand in the B-active site (either two water molecules or a diatomic molecule), its B-factors are much higher than that of the B-heme iron. This is unexpected since the B-factors of the ligand atom directly coordinated to the iron and of the iron should be similar. Computationally this can be achieved by lowering the occupancy of the ligand atoms (see for example (Lucic *et al.*, 2021)). However, from a chemical point of view an unligated heme iron is even more unexpected. The value of the occupancy of the ligand affects the refined distance between both ligand atoms; it is shorter for lower occupancy (1.8 Å for two fully occupied waters, 1.6 Å for two half occupied water molecules, and similar findings for a peroxide ligand). This issue prevents both an assignment of the chemical nature of the ligand based on bond length and a direct comparison of these values obtained from structures derived from the ID29 and Cristallina-MX data. For all refinements we assumed fully occupied heme ligands. Since it was not possible to identify the chemical nature of the ligand in the B-molecule (possibly a hydroperoxo) or for very short X-ray spacings (possibly hydroxide, compound I or II) without additional orthogonal information, we modelled two water molecules.

## S2. Heat and Particle Diffusion in SOS Films

Given the high photon flux and microscopic footprint of serial diffraction X-ray beams, the energy absorbed from each X-ray pulse is sufficiently concentrated to degrade or destroy the sample at the X-ray exposure site. By shifting to a new location for each X-ray pulse, this damage is avoided. The shift in location must be large enough to avoid not just the primary region of damage but also any secondary region, often much larger, over which damage diffuses in the interim. This is particularly relevant to SOS measurements, which are typically probed by rastering the SOS film through the X-ray beam such that the beam focus traces out a serpentine pattern of back-and-forth scan lines across the plane of the film. The spacing of measurement sites both within and between the scan lines must be chosen large enough to avoid diffusion-spread damage. Heat can certainly degrade biological samples. Might heat released through X-ray absorption, diffusing across the SOS chip, account for the sample damage observed in the current SOS experiments? This analysis was undertaken to answer that question.

Provided the X-ray absorption leads to a well-defined temperature rise, the governing equation is

$$k \nabla^2 T = \rho c_p \frac{\partial T}{\partial t} \qquad (1)$$

Here $k$ is the thermal conductivity (S.I. units of W $m^{-1}$ $K^{-1}$), $\rho$ is the mass density of the medium ($kg/m^3$), $c_p$ is the specific heat (heat capacity per unit mass, J $K^{-1}$ $kg^{-1}$). For diffusion within the two dimensional SOS sample film, the two-dimensional form of the Laplacian operator must be used. Eqn. (1) equates the time rate of change of energy density within a unit volume to the flow of energy into that volume (J $s^{-1}$ $m^{-3}$ for both). Eqn. (1) can also be written in the form

$$\frac{\partial T}{\partial t} = \left(\frac{k}{\rho\, c_p}\right) \nabla^2 T \ , \quad (1a)$$

where the quantity in brackets is thermal diffusivity $\kappa$ ($m^2$ $s^{-1}$), which characterizes the amount of heat stored relative to heat transferred. Eqn. (1a) has the same functional form as the particle diffusion equation. Hence diffusion of heat maps directly onto diffusion of particles by replacing temperature with particle number density and thermal diffusivity with particle diffusion coefficient.

In a constant pressure process, heat $Q|_p$ *(J)* added to a system increases the enthalpy by an amount $\Delta H$ *(J)* and provided the process is governed by a constant heat capacity $C_p$ *(J/K)*,

$$\Delta H = Q|_p = C_p\, \Delta T \quad (2)$$

$C_p$ is an extensive quantity, but is typically tabulated as an intensive quantity with respect to either mass, $c_p$ *(J kg$^{-1}$ K$^{-1}$)* or volume $c_p|_{vol}$ *(J m$^{-3}$ K$^{-1}$)*, whereby for a volume of mass $m$ and volume $V$,

$$Q|_p = m\, c_p\, \Delta T = V c_p|_{vol}\, \Delta T \qquad (3)$$

Provided the heat capacity is independent of temperature, time, and position, the temperature rise varies in direct proportion to the heat content per unit volume. Either can equally well be employed to characterize heat flow. This discussion will be cast in terms of temperature rise.

If the X-ray energy is absorbed uniformly across the beam footprint and equilibrates uniformly into heat with no energy loss out of the volume, the temperature rise across the beam spot is a uniform

$$\Delta T_0 = \frac{Q|_p}{m\, c_p\, \Delta T} = \frac{Q|_p}{V c_p|_{vol}} = \frac{Q|_p}{L_x L_y L_z c_p|_{vol}} \quad (4)$$

This assumes that the thermal behavior of the system can be characterized with a single, constant heat capacity $c_p$ and that the X-ray pulse duration is short relative to thermal diffusion times.

The pertinent question is how this absorbed energy will raise the temperature at other points in the system as the heat diffuses away from the deposition site. In an SOS chip this might, for example, be the next grid site to be probed, situated one grid spacing $\Delta x$ away along the current scan line. Were thermal energy to distribute itself uniformly across a cylindrical volume $\pi r^2 L_z$ as it spreads radially outwards, the temperature at the next scan point (at $r = \Delta$ x) would clearly jump abruptly by

$$\Delta T \;=\; \frac{L_x L_y}{\pi \Delta x^2}\,\Delta T_0 \qquad (5)$$

when the spreading heat reaches that point. If the grid spacing is much larger than the footprint dimensions of the X-ray beam, the resulting temperature rise will necessarily be quite small. Putting in dimensions (in $\mu m$) of the experimental X-ray footprint and the smallest scan point spacing investigated, it follows that

$$\frac{\Delta T}{\Delta T_0} \;=\; \frac{2 \bullet 4}{\pi\, 25^2} \;=\; 0.004 \qquad (6)$$

specifically, a 4 mC temperature rise at the nearest neighbor point per degree C of initial X-ray-induced temperature rise at the exposure point. For an initial temperature rise of 100 C this is a 0.4 C. Two grid spacings away (replacing the 25 µm with 50 µm), the corresponding prediction is 1 mC/C. These are very small numbers. Of course, the spatial profile of the diffusing heat is not actually uniform (other than very near $t = 0$). In fact, the Central Limit Theorem requires that any initial temperature profile must evolve with time into a Gaussian distribution of width $\sigma(t) \propto (\kappa t)^{1/2}$ where $\kappa$ is the thermal diffusivity and $t$ the time after deposition. In a topological sense, however, the Gaussian profile is simply a "smeared out" version of the uniform "top-hat" profile as seen in the plot to the right, showing the actual spatial profile $T(r;t)$ at $t$ = 4.32 ms, the interval between X-ray pulses in the current experiment (the maximum of the uniform profile has been set equal to that of the Gaussian). The seemingly oversimplistic example is therefore actually quite relevant.

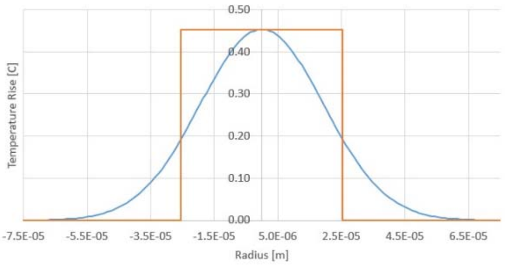


The critical point is that, in the current SOS experiments, the volume into which the heat must spread to reach an adjacent grid point is very large compared to the volume into which the X-ray energy is initially deposited. The 2D geometry dictates that the volume over which the heat is distributed therefore increases as the square of the distance from the deposition point. The thermal energy density and so the temperature change must correspondingly decrease as the inverse square of this distance. In addition, since the individual heat pulses are released at finite intervals set by the X-ray exposure rate, the energy deposition is spread over time as well as space. The rapid drop with radius is the dominant factor for nearby exposures, but the temporal spreading is germane to earlier, distant exposures (say, from the previous or earlier scan lines), resulting in these contributing very little temperature rise. These simple arguments indicate that X-ray induced heating of yet to be exposed crystals is likely not a major problem.

### S 2.1 Numerical calculations – Excel spreadsheet

To explore X-ray-induced heating of SOS samples more rigorously, we carried out numerical calculations in a *(r,t)* array on a spreadsheet. The top row of the array was loaded with an assigned initial temperature profile. For simplicity, the ambient temperature was taken to be 0 C, so that *T(r,t)* itself can be associated with the temperature rise *ΔT(r,t)*. Given that the heat diffusion equation is linear in *T*, the results of the spreadsheet calculation scale directly and linearly with the initial temperature rise. The spreadsheet results can consequently be simply re-scaled to correspond to any initial temperature rise. For convenience, we choose the initial X-ray-induced temperature rise to be 100 C. Higher initial temperature rises would anyway likely involve transfer of heat into other energy forms through vaporization, chemical reactions, ionization, etc., and Eqn. (4) would have to be modified accordingly.

The initial temperature profile *T(r,0)* in the first row of the spreadsheet array is propagated downward row to row through the array by means of standard difference equations. The radial spatial profile *T(r;t)* at a specific time *t* is contained in each row of the spreadsheet array. The time history *T(t;r)* at a specific radius *r* is contained in each columns. These can be plotted as desired and the plots will update as the input parameters are changed, which is a particularly useful feature of spreadsheet modeling.

To characterize the thermal heating at a chosen scan site, it is then only necessary to identify the distance to each contributing X-ray exposure, extract the corresponding *T(t;r)* values from the column for the radius equal to that distance, shift the time zero of column vector to correspond to the correct exposure time, tabulate the results, and sum them. The computations, numerical results, and conclusions are discussed below. The spreadsheet is included in this supplementary information.

Modeling assumptions:

(1) Cylindrical geometry in the SOS plane with only r-dependence only,

(2) No heat transferred into or out of the SOS layer to the polymer bounding films or environment,

(3) X-ray exposure is effectively a delta function at t=0 relative to other characteristic times,

(4) The initial temperature profile is due only to the X-ray exposure,

(5) Thermal conductivity and heat capacity of SOS sample are isotropic and constant.

It will very difficult to identify $k$ and $c_p$ for actual SOS sample media. Values for some possibly representative materials are listed below, given both in the units in which the parameters were published and, in red, as converted into S.I. standard units as employed throughout the spreadsheet.

Liquid decane, a hydrocarbon chain molecule, $C_{10}H_{22}$:

$k$ = 0.1339 W m$^{-1}$ K$^{-1}$ (25 C)

$c_p$ = ((315.46 J K$^{-1}$ mol$^{-1}$)/[(142.286 g·mol$^{-1}$))*(1000 g/kg)] = 2217 J K$^{-1}$ kg$^{-1}$ (25 C)

$\rho$ = (0.730 g mL$^{-1}$) (1 kg/1000g) (1E6 mL/m$^3$) = 730 kg/m$^3$

$c_p|_{vol} = \rho\, c_p$ = 1.618 MJ K$^{-1}$ m$^{-3}$

Vaseline Petroleum Jelly (Petrolatum) the values are similar:

$k$ = 0.804 BTU inch hr$^{-1}$ ft$^{-2}$ F$^{-1}$ (*0.1441 W m$^{-1}$ K$^{-1}$/ BTU inch hr$^{-1}$ ft$^{-2}$ F$^{-1}$)

= 0.1159 W m$^{-1}$ K$^{-1}$ (at 135 F/57.2 C)

$c_p$ = 0.748 BTU lb$^{-1}$ F$^{-1}$ (*4186 J kg$^{-1}$ K$^{-1}$/BTU lb$^{-1}$ F$^{-1}$) = 3131 J kg$^{-1}$ K$^{-1}$ (at 130 F/54.4 C)

$\rho$ = 53.060 lb ft$^{-3}$ (*16.018 kg m$^{-3}$/lb ft$^{-3}$) = 850 kg m$^{-3}$ (at 136 F/57.8 C)

$c_p|_{vol}$ = 2.661 MJ K$^{-1}$ m$^{-3}$

$H_2O$ (water has an unusually high thermal conductivity and high heat capacity)

$k$ = 0.6065 W m$^{-1}$ K$^{-1}$ (25 C)

$c_p$ = 4186 J·K$^{-1}$·kg (25 C)

$\rho$ = 997 kg m$^{-3}$ (25 C)

$c_p|_{vol}$ = 4.173 MJ K$^{-1}$ m$^{-3}$ (25 C)

Expressing the Laplacian of Eqn. (1) in cylindrical coordinates and with $r$-dependence only (no azimuthal ($\theta$) or axial ($z$) dependencies), Eqn. (1) becomes

$$\frac{\partial^2 T}{\partial r^2} + \frac{1}{r}\frac{\partial T}{\partial r} = \frac{\rho\, c_p}{k}\frac{\partial T}{\partial t} \qquad (7)$$

which is to be solved given an initial spatial temperature profile $T(r,0)$, as induced by the X-ray absorption, plus any relevant boundary conditions. The X-ray absorption is taken to be instantaneous at time $t = 0$ and there is no subsequent heat transfer into or out of the SOS sample layer.

Eqn. (7) can be transformed into a difference equation and solved numerically. A first order (O($\delta t$)) forward difference approximation is best for $t$ so that the $t$ formula at a given time refers back only to the previous time step. More accurate second order (O($\delta r^2$)) centered difference approximations can be used for $r$. Accordingly,

$$\left.\frac{\partial T}{\partial t}\right|_{r,t} \rightarrow \frac{T(r,\ t+\delta t) - T(r,t)}{\delta t} \quad (8)$$

$$\left.\frac{\partial T}{\partial r}\right|_{r,t} \rightarrow \frac{T(r+\delta r,\ t) - T(r, t-\delta r)}{2\delta r} \quad (9)$$

$$\left.\frac{\partial^2 T}{\partial r^2}\right|_{r,t} \rightarrow \left(\frac{T(r+\delta r,\ t) - 2\,T(r,t) + T(r-\delta r,\ t)}{(\delta r)^2}\right) \quad (10)$$

With these, the governing equation becomes

$$k\left(\frac{T(r+\delta r,\ t) - 2\,T(r,t) + T(r-\delta r,\ t)}{(\delta r)^2}\right) + \frac{k}{r}\left(\frac{T(r+\delta r,\ t) - T(r-\delta r, t)}{2\,\delta r}\right) = \rho\, c_p\left(\frac{T(r,\ t+\delta t) - T(r,t)}{\delta t}\right) \quad (11)$$

where $\delta r$ and $\delta t$ are steps in radius and time to be used in the numerical integration. This can be re-written as

$$\xi\left(T(r+\delta r,\ t) - 2\,T(r,\ t) + T(r-\delta r,\ t) + \frac{\delta r}{2\,r}\left(T(r+\delta r,\ t) - T(r-\delta r,\ t)\right)\right)$$

$$= \left(T(r,\ t+\delta t) - T(r,\ t)\right) \quad (12)$$

whereby the various parameters have been rolled into a single dimensionless parameter $\xi$,

$$\xi = \frac{k\,\delta t}{\rho\, c_p\,(\delta r)^2} \quad (13)$$

Since changes in $T(r, t)$ are proportional to $\xi$, this parameter should ideally be << 1. Since $k/(\rho\, c_p)$ alone is small ($O(10^{-7})$), it is necessary to choose $\delta t$ and $\delta r$ such that $\delta t << 10^7(\delta r)^2$. Of course, it is also necessary to that $\delta r << r$ and $\delta t << t$.

Solving for $T(r+\delta t, r)$ and collecting like terms

$$T(r,\ t+\delta t) = T(r,\ t) - 2\xi\, T(r,\ t)$$

$$+ \xi T(r+\delta r,\ t) + \xi\frac{\delta r}{2\,r}T(r+\delta r,\ t)$$

$$+ \xi T(r-\delta r,\ t) - \xi\frac{\delta r}{2\,r}T(r-\delta r,\ t) \quad (14)$$

or

$$T(r,\ t+\delta t) = (1-2\xi)T(r,\ t)$$

$$+ \xi \left(1 + \frac{\delta r}{2\,r}\right) T(r + \delta r,\ t)$$

$$+ \xi \left(1 - \frac{\delta r}{2\,r}\right) T(r - \delta r,\ t) \qquad (15)$$

Applied to a temperature profile at time $t$, this allows a subsequent temperature profile to be generated for time $t+\delta t$ using the temperatures at $r$, $r+\delta r$, and $r-\delta r$ at time $t$. This is easily coded into a *(r,t)* spreadsheet array with the spatial profiles $T(r;t)$ stored in the rows with increments of $\delta r$ from one cell to the next and with time increasing by increments $\delta t$ in stepping downwards from row to row.

This numerical approach will likely balk at discontinuities in either the $T$-profile or its slope (infinite first and second derivatives, respectively). It is therefore best to choose a continuous, smoothly varying initial profile. Possibly the simplest form which allows both the spot radius ($a$, HWHM) and the edge thickness to be chosen is an exponential form as given in an EXCEL formula by

=$B4$8+$B$47*IF(ABS(K$2)<=$B$45,0.5+ 0.5*(1-EXP((ABS(K$2)-$B$45)/$B$46)), 0.5*EXP(-(ABS(K$2)-$B$45)/$B$46))

Here $B$45 is the spot radius (spot HWHM), $B$46 sets the thickness of the spot edge, and $B$47 is the temperature rise at the spot center at time zero (here 10 K), and $B$48 is the ambient (here zero K),

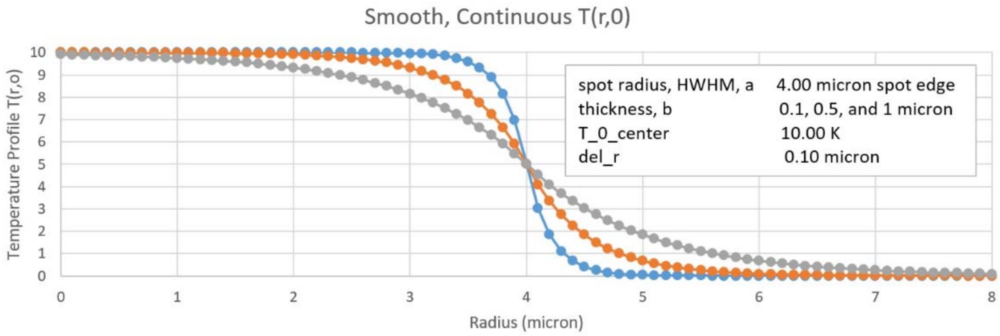


**Supplemental Fig. S9** Defining a discontinuity-free shape for the initial temperature distribution

Given the cylindrical symmetry of the problem, the slope of the $T(r;t)$ profile at $r=0$ must be zero. The simplest way to force this is to pick the range of $r$ and a profile $T(r,0)$ that is symmetric about $r = 0$.

The $r^{-1}$ dependence will cause problems if a grid point happens to land on $r = 0$. This is easily avoided by choosing the grid to symmetrically avoid $r = 0$, for example by setting the $r$-coordinate of the central grid points to straddle $r=0$ by $\delta r/2$.

To accommodate the $r - \delta r$ and $r + \delta r$ of the adjacent columns, it is useful to force the spatial profiles to the ambient temperature (generally 0 C, for reasons mentioned above) in the first and last columns of the spreadsheet. It will be seen below that this boundary restriction can later be circumvented. The number of columns in the array should then be large enough that the temperature profiles *T(r;t)* approach zero before reaching the boundary *r*- values. The spacing $\delta r$ of the *r*-points must be fine enough to delineate the narrow initial temperature profile, yet coarse enough to allow the numerical calculations to extend out to sufficiently large *r*. Smaller values of $\delta t$ deliver more accurate numerical results, but also limit the overall *t* range for a chosen array size. Note that $\delta r$ and $\delta t$ cannot be chosen independently since they are constrained by the requirement that stability parameter of Eqn. (3) be significantly less than unity. A balance must be struck among these conflicting objectives.

Eqn. (15) was encoded into an Excel spreadsheet *(r,t)* array of 250 columns (*r*-values) by 2050 rows (*t* values) with $\delta r$ = 1 µm and $\delta t$ = 10 µs (but entered into the spreadsheet in S.I. units!). Spatial temperature profiles *T(r;t)* were graphed by plotting the values in the row corresponding to a chosen *t* against the *r*-values provided in the row just above the array. Temporal temperature profiles *T(t;r)* at a chosen distance *r* were graphed by plotting values in the column corresponding to a chosen *r* against the *t*-values provided in the column just to the left of the array. Should the grid spacing in *r* be changed, each column then corresponds to a new *r*-value, which nonetheless is correctly labeled in the *T(t)* legend. The columns can easily be re-assigned in the Excel "Chart Design," "Select Data" menu just by changing the column letters. Analogous changes result if the grid spacing in *t* is altered. When re-assigning either the rows or columns, the header cell for the new row or column should also be reassigned in the "Select Data" menu so that the correct identifications are displayed in the chart legend.

### S2.1.1 Results

Plots of *T(r;t)* for various *t,* taken directly from the rows of the spreadsheet array, are plotted in Fig. S10. The input parameters for these computations are shown in the table to the right. The focal spot radius for the X-ray beam was set to 1.60 µm to give the same circular spot area as the 2 µm by 4 µm rectangular area of the ID29 X-ray spot. The X-ray-induced temperature rise in this focal spot was assigned to be 100 C relative to an assigned ambient of 0 C as discussed above. The density and thermal properties are those of Vaseline from above, but these could easily be changed to any desired values. The spreadsheet and plots will update automatically.

| | | |
|---|---|---|
| **SOS Sample Mass and Thermal Characteristics** | | |
| SOS sample | Vaseline | --- |
| SOS sample thermal conductivity, *k* | 0.1159 | W/(K m) |
| SOS sample mass density, ρ | 850.0 | kg/m^3 |
| SOS sample mass heat capacity, *c_p* | 3131 | J/(kg K) |
| SOS sample volumetric heat capacity, *c_p/vol* | 2.661E+06 | J/m^3 K) |
| *k/(* ρ *c_p )* = *(k/c_p/vol)* | 4.355E-08 | m^2/s |
| | | |
| **Parameters for Numerical Solution** | | |
| del_r | 1.00E-06 | m |
| del_t | 1.00E-05 | s |
| ξ (characteristic dimensionless parameter) | 4.35E-01 | --- |
| | | |
| **Smooth Continuous Initial Profile T(r,0)** | | |
| X-ray focal spot radius | 1.60E-06 | m |
| X-ray spot edge width | 1.00E-07 | m |
| Initial temperature rise in X-ray focal spot | 100 | C |
| Ambient Temperature, T_amb | 0 | C |

The ordinate range in the upper panel of Fig. S10 is 0 C to 5 C. In accordance with the Central Limit Theorem, the initial smoothed, flat *T(r;0)* profile of the initial temperature distribution (at 100 C, well above this ordinate range) is seen to rapidly evolve with time into a Gaussian-like radial profile *T(r;t)*.

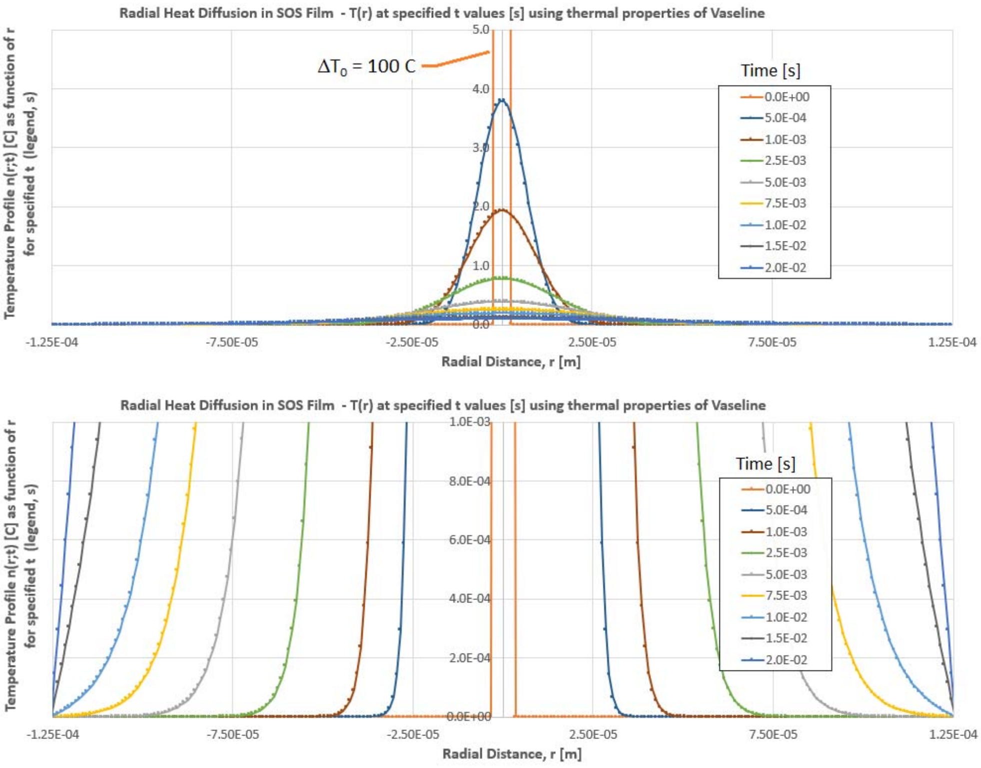


**Supplemental Fig. S10** *T(r;t)* spread sheet results on two scales to show peaks (top frame) and bases (bottom)

In the lower panel the vertical scale has been magnified by a factor of 5000 to yield an ordinate range of 0 to 1mC to display the bases of these *T(r;t)* distributions. Consequences of pinning the temperature rise to zero at the boundaries of the spreadsheet array (at ±126 µm) are evident in the outermost two profiles (times of 15 and 20 ms). The distortions appear (on this very expanded ordinate scale) for profiles sufficiently broad that would extend out of the array. For the current array size, the results are therefore strictly speaking valid only up to about $t$ = 10 ms and $r$ = 100 µm, although the distortions seen here are too small to introduce significant problems. They can be eliminated either by increasing the size of the spreadsheet array or by replacing the profiles at these times with analytic functions based on the Central Limit Theorem, as discussed and illustrated below.

Plotting spreadsheet columns rather than rows gives the results that are the actual interest of this spreadsheet calculation, namely *T(t;r)* plots of temperature as a function of time at specified radii. Examples of *T(t;r)* plots are plotted in Fig. S11 at radii equal to 1, 2, 3, and 4 multiples of the 25.5 µm scan steps along a scan line. Following an X-ray absorption event at $t$ = 0 at the current grid point, these curves are the temperature time-histories observers would measure if stationed at the nearest neighbor scan point, the next-nearest neighbor point, the next-next-neighbor point, etc. Alternatively, they also correspond to the temperature time-histories an observer at one specified scan point would measure for an X-rate absorption event one scan step away, two scan steps away, three scan steps away, etc., corresponding to exposures occurring one scan interval earlier, two scan intervals earlier, three scan intervals earlier, etc. To put these time histories on the same time scale, each *T(t;r)* must shifted in time to place its starting point on the time of the corresponding exposure. Once this has been done, the curves can be summed to obtain the overall temperature rise induce by the contributing exposures.

The short vertical bars mark the X-ray pulses times at a pulse rate of 231.25 Hz (a scan interval of 4.32 ms), as in the experiment. The time shifts needed to synchronize the curves with their corresponding exposure times can clearly be substantial relative to the temporal features of the curves, and the more so the further removed in time the relevant exposure happens to be.

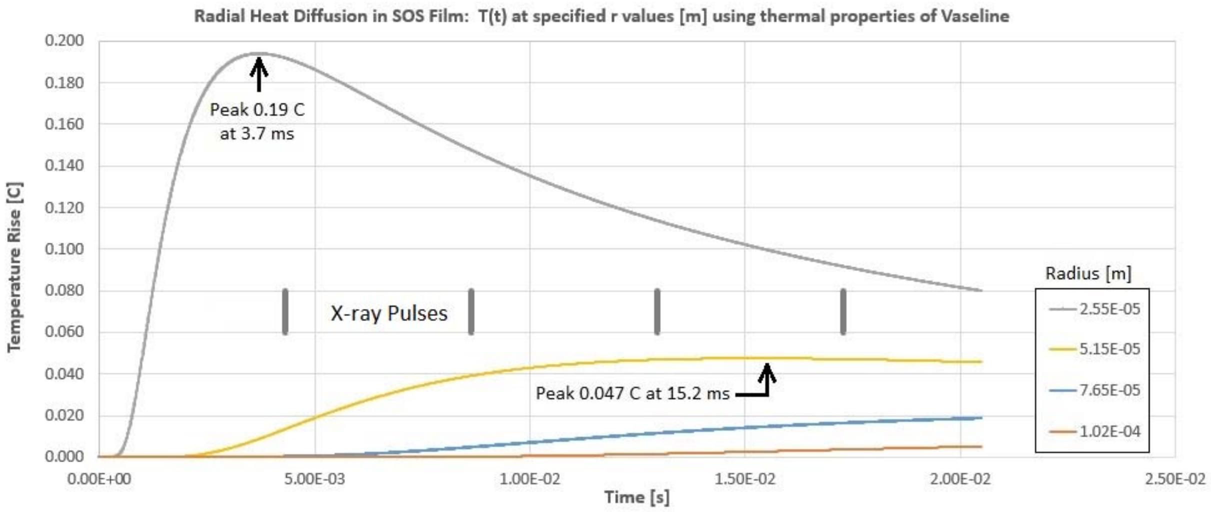


**Supplemental Fig. S11** *T(t;r)* spreadsheet plots corresponding to exposures $r = 1$, 2, 3, and 4 grid spacings away

The plots of Fig. S11 predict that an X-ray exposure will raise the temperature at a scan point one grid spacing away by 0.19 C. This peak temperature is attained at about 3.7 ms after the exposure, which happens to be roughly equal to the 4.32 ms time interval between X-ray pulses at ID29. Hence the scan fails to “outrun” heat transfer to the nearest neighbor grid point. Two grid spacings away the picture is quite different. The corresponding peak temperature rise is only 0.05 C and comes at a much longer 15.2 ms, meaning that the scan significantly outruns the heat transfer to this more distant point. At larger radii the temperature rise is still smaller and even more delayed. Given the assumed initial X-ray-induced temperature rise of 100 C, the temperature peaks corresponding to exposures at the nearest and next-nearest scan line sites correspond to 2 mC and 0.05 mC per 1 C of initial temperature rise respectively. This agrees well with the 4 mC/C predicted by the simplistic model presented in the introduction.

The plots of Fig. S11 already offer some relevant insights. Given the rapid decrease of the temperature rise with distance from the X-ray exposure point, it is likely that only the very nearest X-ray exposures need be considered when integrating over the complete exposure history. For a transit along a specified scan line, the *T(t;r)* curves of Fig. S11 are exactly those needed to determine the temperature rise at the next-to-be-probed on the scan line due to the immediately preceding four X-ray exposures. The curves need only be time-shifted, as discussed above, and summed.

However, a complication immediately becomes apparent. The lowermost curve of Fig. S11 must be shifted to the left by four of the 4.32 ms scan intervals or 17.28 ms, the next lowest by 12.96 ms, etc.

These are significant fractions of the overall 20.5 ms overall length of the curves. It would be very useful to extend the *t*-range of Fig. S11 by at least a factor of ten, with a commensurate extension of the *r*-range. To do so by increasing the size of the spreadsheet would be cumbersome if even possible. Luckily the Central Limit Theorem offers an easy way out of this quandary, as described below.

### S 2.1.2 Extending the spreadsheet results with analytic formulas based on the Central Limit Theorem

The Central Limit Theorem states that, for any initial temperature distribution, *T(r;t)* should evolve with time into a Gaussian spatial profile. This can easily be investigated by fitting *T(r;t)* profiles from spreadsheet to Gaussian profiles for a range of *t*-values, extracting the Gaussian widths *σ(t)* for each *t*, and plotting the *σ(t)* values as a function of *t*. Provided the *t*-range of the spreadsheet is large enough that the Gaussian can evolve to its limiting form, a general power law expression with an exponent of ½ should result. This, in turn, can be inserted into the Gaussian expression to yield a general analytic formula for *T(r,t)* that allows the numerical results of the spreadsheet to be extended to larger values of *r* and *t*.

The width parameter σ enters not just into the exponent of Gaussian expression but also into its prefactor. In the latter, its power depends on the dimensionality of the system under consideration and is expected to be two for 2D diffusion. Nonetheless it is prudent to explicitly verify the power for the current situation of cylindrical symmetry having only an *r*-dependence. Inserting a trial functional form

$$T(r,t) \;=\; \frac{A}{\sigma^n}\, e^{\left(\frac{-r^2}{2\,\sigma^2}\right)} \;, \qquad (16)$$

into Eqn. (1a) yields the expression

$$-2 \;+\; \frac{r^2}{\sigma^2} \;=\; \left(-n\,\kappa\,\sigma \;+\; \kappa\frac{r^2}{\sigma}\right) \qquad \text{with} \qquad \kappa \;=\; \frac{\rho\, c_p}{k}\text{, the thermal diffusivity} \qquad (17)$$

Equating coefficients of the $r^2$ terms yields

$$\sigma\,\frac{\partial\sigma}{\partial t} \;=\; \kappa \qquad \text{which integrates immediately to} \qquad \sigma^2 \;=\; \frac{2t}{\kappa} \;+\; const, (18)$$

so $n = 2$. Likewise, equating coefficients of the *t*-independent terms yields

$$\sigma^2 \;=\; \frac{4t}{n\kappa} \;+\; const, \quad (19)$$

making $n = 2$ indeed the consistent choice. The constant $A$ can be set by use of Eqn. (4).

The $\sigma(t)$ fitting was carried out for $T(r;t)$ profiles in the $t$-range from 0.5 ms up to 4.5 ms. The $\sigma(t)$ values extracted from the Gaussian fits to the spreadsheet results are plotted as a function of $t$ in Fig. S12. An Excel power law "trendline" gave an exponent of 0.4938, in good agreement with expected value of ½. Setting the exponent to exactly ½ and performing a least-squares-fit by adjusting the amplitude delivered the expression

$$\sigma(t) = 2.960x10\ t^{1/2}, \qquad (20)$$

This was then inserted into the Eqn. (16) to obtain the desired expression,

$$\frac{\Delta T}{\Delta T_0} = \frac{1.953x10^{-3}}{t}\ exp\left(\frac{-5.707x10^{6}\ r^2}{t}\right) \quad (21)$$

At low end of the chosen $t$-range, superimposing curves of this form on Fig. S10 does not yield overly impressive agreement with the numerically calculated $T(r;t)$ curves from the spreadsheet. At long $t$ values the agreement is much better. The inferior fits at low $t$ may indicate that the calculated $T(r;t)$ curves at those shorter times have not yet fully evolved to their limiting Gaussian form.

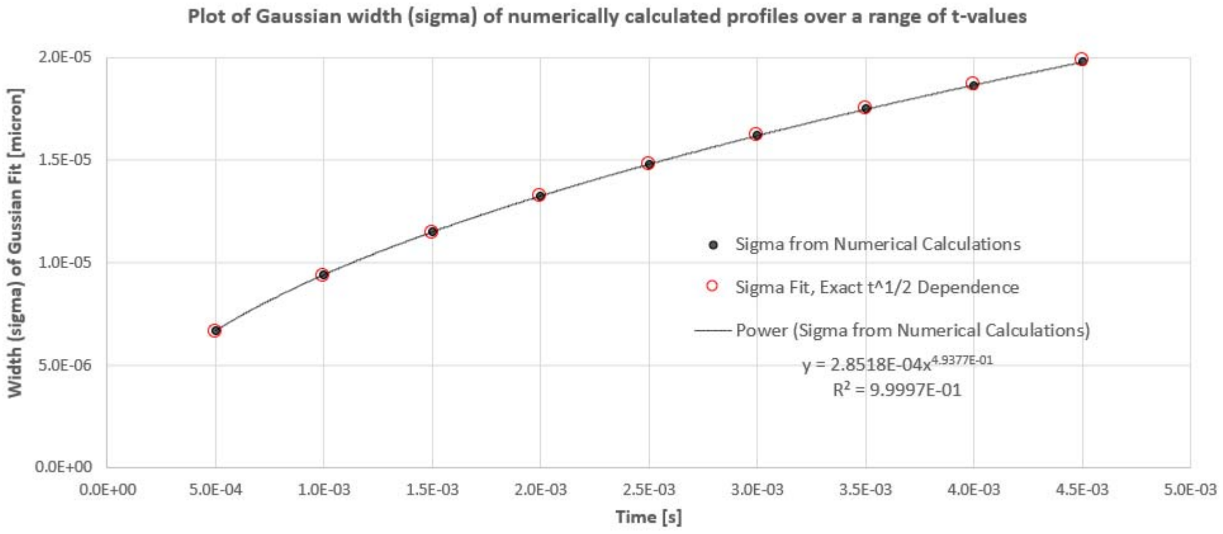


**Supplemental Fig. S12** $\sigma(t)$ values extracted by fitting spreadsheet $T(r;t)$ results to Eqn. (16)

The goodness of fit of Eqn. (21) at large $r$ and $t$ was verified by superimposing $T(r;t)$ curves of this form for various $t$-values onto the extreme left-hand side of the lower panel of Fig. S10, as presented in the figure to the right. The lightweight solid line

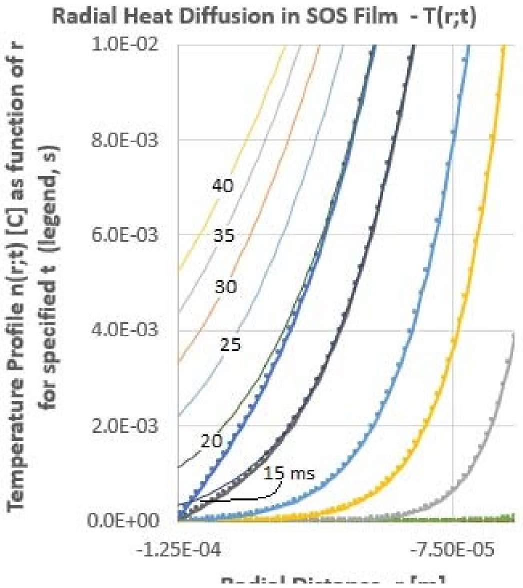

curves are those from Eqn. (21), each labeled with its corresponding time. The two lowest curves, for 15 and 20 ms, correspond to the "boundary-pinned" *T(r;t)* curves mentioned above in conjunction with Fig. S10. The analytic curves, of course, do not display the pinning distortion. They moreover taper in smoothly to perfectly match the spreadsheet curves at smaller *r*-values. The analytic expression therefore seems to function extremely well.

Using the analytic expression Eqn. (21), the plots of Figure S11 were extended to much longer times as follows: Columns of *T(r;t)* were copied from the spreadsheet array and shifted in time origin by the requisite integer number of the 4.32 ms pulse intervals to synchronize them with their X-ray exposure time. Eqn. (21) was then employed to add additional points to the tail off each curve.

The curves were then summed to obtain the integrated temperature rise due to the sequence of X-ray pulses. The results are plotted in Fig. S13. The thick red line curves are exactly those of Fig. S11, just time-shifted. The thin sections of the red curves show where the curves have been extended using Eqn. (21). The sum of these four curves is shown as the thick blue curve. This is then the computed temperature rise at the next-to-be-probed site of a scan line due to the four immediately preceding X-ray exposures along that line, under the conditions of Fig. S10 (25.5 µm spacing of X-ray exposures, X-ray pulse rate of 231.25 Hz, assumed initial temperature rise of 100 C per X-ray exposure). An enlarged view of the region near t=0 is provided in the inset.

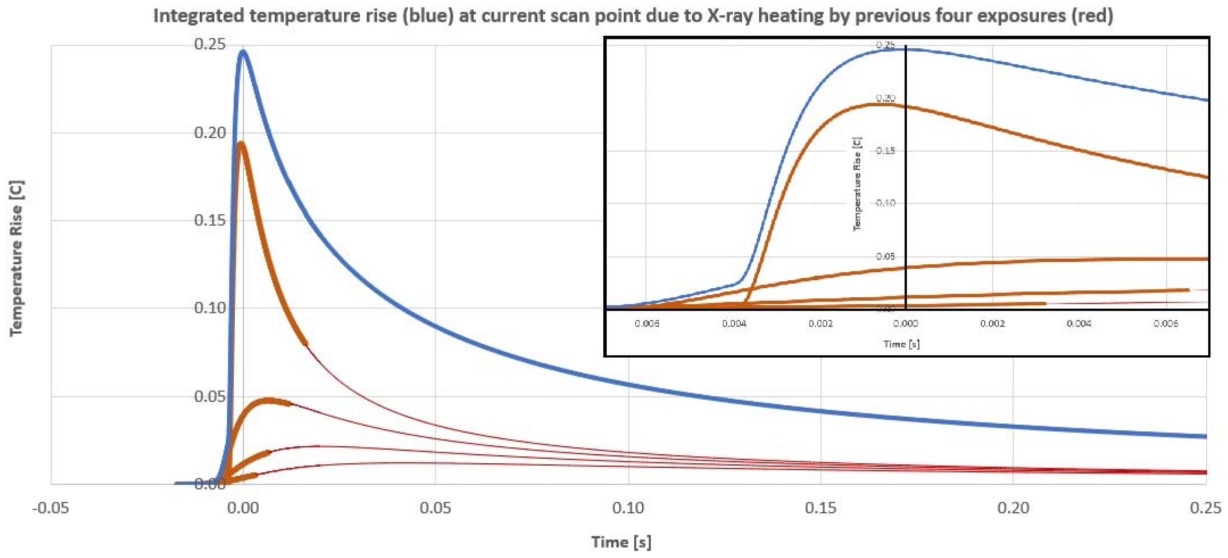

**Supplemental Fig. S13** Summing over four immediately preceding intra-line exposures along a scan line to get an overall temperature rise.

**S 2.2 Conclusions**

Reading from the summation (blue) curve of Fig. S13, the maximum temperature rise is predicted to be just under 250 mC, coming at about 10 µs before the next-to-be-probed scan point receives an X-ray pulse. That temperature rise is dominated (78%) by heat diffusing from the immediately adjacent previous exposure along the scan line, with a smaller contribution (16%) from the next-nearest X-ray pulse on the scan line, and still less (5%, and 1%) from the previous two pulses. Contributions from more distant exposures along the scan line will clearly be in the single mC range and below. The largest maximum temperature rise will occur at scan points just beyond the direction reversal at the end of a long scan line, that being where the next-to-be-probed scan site is in closest spatial and temporal proximity to the largest number of previous X-ray exposures. Yet even at those points the temperature rise will be only marginally larger than in Fig. S13. Adding in estimated contributions from more distant exposures, say 100 exposures contributing perhaps 0.5 mC each on average, brings the overall temperature rise up to roughly 1 C or, in scalable terms, 10 mC/C relative to the assumed initial 100 C temperature rise. This represents the global maximum temperature rise experienced at a representative scan point. The calculations of Fig. S13 correspond to the smallest spacing of exposures employed in the actual experiments and, at larger separations, the temperature rise will be even less. The slow decay of the temperature rise at a given scan point (long tail of the blue curve in Fig. 13) might be a major concern at high pulse rates and closer separations. At the current rate (231.25 Hz) and spacing (25.5 µm), the very rapid decay in peak temperature rise with distance from previous exposures seems to preclude this as a concern. Moreover, the current modeling neglects entirely the flow of heat out through the SOS film and into the surrounding environment, which should markedly increase the rate of heat dissipation. Consequently, these calculations indicate that thermal heating due to energy absorption from X-ray pulses is unlikely to be a concern when scanning SOS films at synchrotron sources. Other experimental concerns, such as ionization, photo-generation of chemically reactive species, or (at XFELs) puncture of the SOS films leading to sample desiccation would appear to be more relevant damage issues.

This information contained in Fig. S13 can also be presented as in Fig. S14 to add “genuine” flesh to the schematic bones of Fig. 1 in the main text. Red circles indicate heat diffusing from the previous four intra-line X-ray exposures along a long scan line, each labeled with its calculated contribution to

the temperature rise at the next-to-be-scanned point at the instant that point is probed. This information is also keyed into the line thickness of each circle.

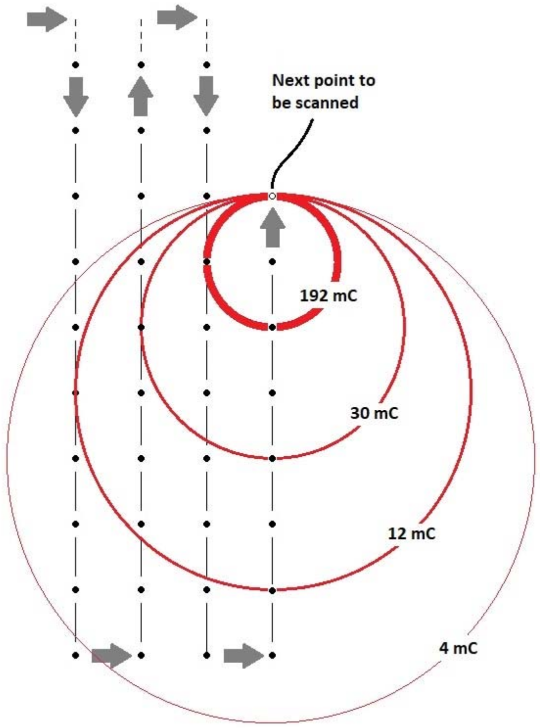


**Supplemental Fig. S14** Diagrammatic depiction of the numerical results.

**S 2.3 Extension to a dilute concentration of particles diffusing through an isotropic SOS medium**

The X-ray beam pulse not only heats the sample but also forms free elections, radicals, and excited species that can diffuse outward from the region illuminated by the X-ray beam. In addition, these species can react chemically and/or relax electronically, both with each other and with the medium, to form new species to diffuse away from the scattering volume. If the highly excited and/or reactive species transmute rapidly into stable species on a time scale that is short relative to the relevant diffusion time scales, the concentration of these stable species will develop in a manner analogous to the temperature profiles developed above.

Given a dilute concentration *n(r,t)* of neutral atoms or molecules in a medium for which the diffusion constant *D* *($m^2/s$)* is isotropic and independent of time, position, and *n* itself, the governing equation for diffusion of the dilute species is

$$D\,\nabla^2\, n(r,t) \;\; = \;\; \frac{\partial\, n(r,t)}{\partial\, t} \quad (22)$$

As noted, this has the exact same mathematical form as Eqn. (1) as for thermal diffusion by making the simple substitution

$$\frac{k}{\rho\, c_p} \rightarrow D$$

Accordingly, the dimensionless parameter introduced in Eqn. (8) for thermal diffusion becomes

$$\xi \; = \; D\frac{\delta\, t}{(\delta\, r)^2} \qquad (23)$$

By simply changing $\xi$ to this value, the exact same spreadsheet modeling may immediately be applied.

Similar assumptions apply as in the thermal diffusion case:

(1) The geometry is purely cylindrical, with no $\phi$ or $\theta$ dependence (including in initial/boundary conditions),

(2) No particles are lost at the polymer films that bound the SOS sample,

(3) The diffusing species is generated very rapidly relative to subsequent diffusion times,

(4) There is no transformation of the diffusing species once formed,

(5) The diffusion constant *D* is isotropic and independent of *t, r*, and *n(r,t)*,

(6) The parameter $\xi$ is sufficiently small (less than ~0.25 according to the $T$ calculations),

(7) $\delta r << r$ and $\delta t << t$.

A sheet has been added to the current spreadsheet to calculate *n(r,t)* in this fashion. The initial profile *n(r,0)* must be input “manually.” Results are shown in Fig. S15 for a 5 micron X-ray spot diameter with a starting concentration *n(r,0)* = 0.02 nM within the spot region.

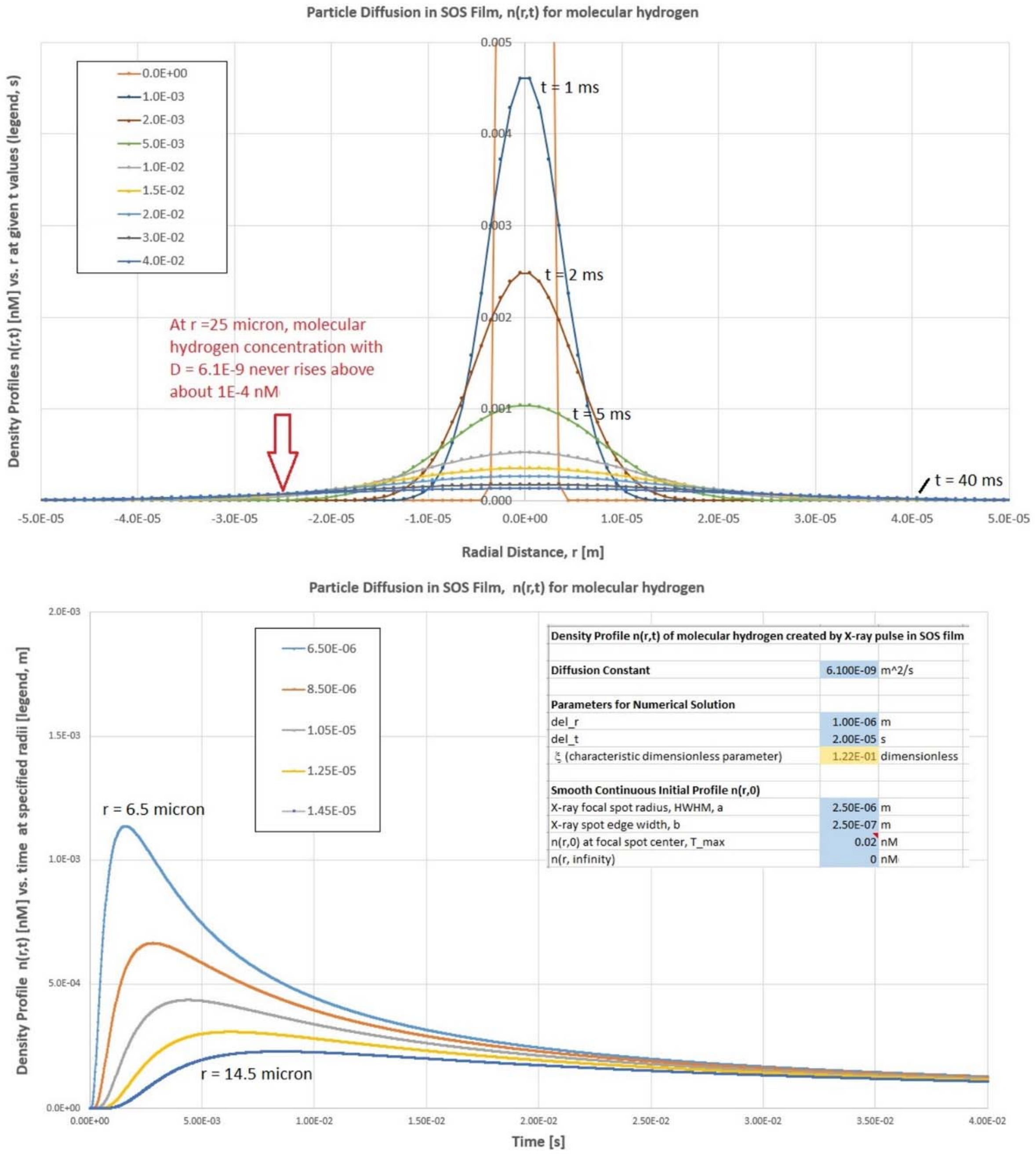


**Supplemental Fig. S15** Example of spreadsheet calculations for $H_2$ diffusing through an isotropic SOS medium. The diffusion constant for $H_2$ in water was obtained from (Wang *et al.*, 2023).

## References

Doak, R. B., Shoeman, R. L., Gorel, A., Nizinski, S., Barends, T. R. M. & Schlichting, I. (2024). *J Appl Crystallogr* **57**, 1725-1732.
Ebrahim, A., Moreno-Chicano, T., Appleby, M. V., Chaplin, A. K., Beale, J. H., Sherrell, D. A., Duyvesteyn, H. M. E., Owada, S., Tono, K., Sugimoto, H., Strange, R. W., Worrall, J. A. R., Axford, D., Owen, R. L. & Hough, M. A. (2019). *IUCrJ* **6**, 543-551.
Lucic, M., Wilson, M. T., Svistunenko, D. A., Owen, R. L., Hough, M. A. & Worrall, J. A. R. (2021). *J Biol Inorg Chem* **26**, 743-761.
Meents, A., Gutmann, S., Wagner, A. & Schulze-Briese, C. (2010). *Proc Natl Acad Sci U S A* **107**, 1094-1099.
Wang, S. J., Zhou, T., Pan, Z. Q. & Trusler, J. P. M. (2023). *J Chem Eng Data* **68**, 1313-1319.
